# Progressive Insights into 3D Bioprinting for Corneal Tissue Restoration

**DOI:** 10.1002/adhm.202503372

**Published:** 2025-09-25

**Authors:** Ilayda Namli, Deepak Gupta, Yogendra Pratap Singh, Pallab Datta, Muhammad Rizwan, Mehmet Baykara, Ibrahim T. Ozbolat

**Affiliations:** ^1^ The Huck Institutes of the Life Sciences Penn State University University Park PA 16802 USA; ^2^ Engineering Science and Mechanics Department Penn State University University Park PA 16802 USA; ^3^ School of Healthcare Science and Engineering Vellore Institute of Technology Vellore Tamil Nadu 632014 India; ^4^ Department of Pharmaceutics National Institute of Pharmaceutical Education and Research Kolkata West Bengal 700054 India; ^5^ Department of Biomedical Engineering Michigan Technological University Houghton MI 49931 USA; ^6^ Department of Ophthalmology Faculty of Medicine Bursa Uludag University Bursa 16059 Türkiye; ^7^ Department of Biomedical Engineering Penn State University University Park PA 16802 USA; ^8^ Materials Research Institute Penn State University University Park PA 16802 USA; ^9^ Department of Neurosurgery Penn State University Hershey PA 16802 USA; ^10^ Penn State Cancer Institute Penn State University Hershey PA 17033 USA; ^11^ Department of Medical Oncology Cukurova University Adana 01130 Türkiye; ^12^ Biotechnology Research and Application Center Cukurova University Adana 01130 Türkiye

**Keywords:** cornea, collagen fibrils, 3D bioprinting, bioinks, corneal tissue engineering

## Abstract

The complex architecture of the cornea, characterized by specifically organized collagen fibrils and distinct cellular layers, poses significant challenges for traditional tissue engineering strategies to replicate its native function. 3D Bioprinting offers a promising solution by enabling the precise, layer‐by‐layer fabrication of corneal tissues, closely mimicking the essential characteristics needed for vision restoration and long‐term graft success. This Review critically examines the key biomechanical, optical, and structural attributes of the cornea necessary for its effective engineering and accurate 3D bioprinting. It provides a comprehensive overview of different 3D bioprinting modalities utilized for corneal tissue engineering and offers insights into potential improvements. Additionally, it details the requirements for a corneal bioink suitable for 3D bioprinting, ensuring it meets the necessary corneal functions. The Review also delves into the current challenges in 3D bioprinting of corneal tissue and proposes potential solutions to successfully replicate the complex architecture and function of the cornea. Furthermore, it explores innovative approaches such as the use of induced pluripotent stem cells, gene therapy, and cornea‐on‐a‐chip technologies, which hold promise for advancing corneal regeneration. The Review aims to visualize the future of corneal 3D bioprinting and the potential of integrating it with other techniques. Lastly, the review discusses clinical implications, emphasizing the potential of bioprinted corneal implants to address the global donor cornea shortage and significantly improve patient outcomes.

## Graphical abstract

This review explores the potential of 3D bioprinting to replicate the complex structure and function of the human cornea. It highlights key advances in bioink development, printing modalities, and in vivo performance, while addressing current challenges and emerging strategies. The review emphasizes bioprinting's promise to overcome donor shortages and improve outcomes in corneal regeneration.

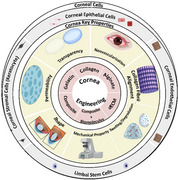

## 1 Introduction

The cornea is an avascular, transparent dome‐shaped uppermost ocular tissue protecting the inner eye from the external environment. Corneal tissue can heal only from minor injuries, but in cases such as severe trauma, deep stromal damage, corneal infections, or chemical burns, it cannot recover completely on its own and requires interventions or treatments such as corneal transplantation, keratoprosthesis, or regenerative therapies like stem cell‐based approaches or bioengineered tissue grafts.^[^ ^1^, ^2^ ^]^ As reported by the World Health Organization, 2.2 million people suffer from visual impairment.^[^ ^3^ ^]^ Corneal blindness contributes to 5% of all patients suffering from blindness worldwide.^[^ ^4^ ^]^ Donor corneas are in short supply to meet the needs of ten million untreated patients in developing nations, so there is a crucial requirement for artificial corneas.^[^ ^5^, ^6^, ^7^, ^8^ ^]^ This severe donor shortage highlights the urgent need for alternative solutions, such as engineering a cornea, to address the growing global demand.

The human cornea is structurally arranged into three main cellular layers, including epithelial, stromal, and endothelial. The cornea is a tissue capable of acquiring atmospheric oxygen due to the deprivation of blood vessels.^[^ ^9^ ^]^ The aqueous‐soluble structural protein found in vertebrate eye lenses, called crystalline, is formed by keratocytes and repairs the cornea during injury and maintains its molecular organization.^[^ ^10^ ^]^ The human corneal endothelial cells (hCEnCs) play a pivotal role in maintaining corneal hydration by regulating the transport of nutrients and fluids across the stromal and epithelial layers. This process is essential for preserving the cornea's transparency and overall health. Limbal stem cells located in the peripheral cornea play a crucial role in the regeneration of the corneal epithelium. Damage to these cells can lead to limbal stem cell deficiency, resulting in keratopathy. In addition to physical injury, infections, and medications, such as corticosteroids, can induce corneal keratosis, further compromising corneal integrity. The presence of metalloprotenases and inflammatory cytokines in tears results in chronic inflammation of the corneal membrane, thereby causing its gradual thinning and bulging outward, known as keratoconus. Human corneal keratoplasty and corneal tissue grafting remain the most preferred surgical interventions for corneal pathologies.^[^ ^11^ ^]^ However, tissue engineering strategies alone are not effective due to the challenges associated with long‐term stability and the difficulty of mimicking the complex layers and structures of the cornea.

Various strategies have been utilized in corneal tissue engineering (CTE), including the use of decellularized corneal scaffolds that preserve the native ECM structure, and the development of biomimetic hydrogels designed to replicate corneal properties.^[^ ^12^, ^13^, ^14^, ^15^ ^]^ Studies have employed different techniques to arrange the stromal microenvironment, such as electrospinning, electrocompaction, and mechanical stretching.^[^ ^16^, ^17^, ^18^ ^]^ Additionally, to create corneal constructs with specific shapes, techniques like molding are used to provide the curved structure of the dome‐shaped cornea, its size, and patient‐specific dimensions.^[^ ^19^, ^20^, ^21^ ^]^ Many studies primarily focus on mimicking the stromal layer, which accounts for 90% of the corneal thickness and provides both transparency and mechanical strength, with emphasis on replicating its organized collagen structure. Although the endothelial and epithelial layers are crucial for hydration and protection, they are often preferred to be reconstructed after developing the stromal scaffold. While these advancements are promising, challenges in achieving highly organized, transparent stromal lamellae and a cohesive multi‐layered structure remain, highlighting the need for further innovation in mimicking the cornea's native architecture. Furthermore, precisely shaping a cornea, including its curved dome structure and individualized dimensions, remains a complex and challenging task.

3D bioprinting has significantly advanced the field of CTE. 3D Bioprinting facilitates the creation of complex structures by sequentially adding layers according to digital designs. This process has been used to create corneal substitutes in a precise and adaptable manner.^[^ ^22^ ^]^ It has introduced novel opportunities, allowing the creation of personalized constructs and accurately mimicking anatomical structures.^[^ ^23^, ^24^ ^]^ Conventional methodologies have encountered difficulties in effectively mimicking the intricate architecture of the cornea, which exhibits a well‐structured collagen framework and distinct cellular arrangement that play a crucial role in maintaining its optical transparency and mechanical characteristics.^[^ ^5^ ^]^ These characteristics are of paramount significance in ensuring optimal visual function and thus require a methodical approach. 3D Bioprinting has the potential to deposit bioinks sequentially, which consist of viable cells and materials that are compatible with biological systems, in a manner that builds up layers and leads to the development of corneal tissue constructs possessing structural and functional characteristics like the native cornea. In addition, the capacity to incorporate various cell types, growth factors, and biochemical signals within these constructs significantly augments their capability to facilitate the regeneration of the cornea. Researchers can accurately position different cell types and replicate the hierarchical structure of the cornea by utilizing bioink formulations that are specifically designed for the corneal tissue. The utilization of bioinks sourced from decellularized corneal tissue or synthetic materials is reported to improve the viability, differentiation, and maturation of cells within constructs.^[^ ^25^, ^26^ ^]^ 3D Bioprinting technology provides the benefits of customization and scalability, which allows for the efficient manufacturing of corneal constructs on a large scale, effectively meeting the increasing global need for corneal transplants.

In this Review, we provide comprehensive insights into the utilization of 3D bioprinting in CTE, highlighting its current advancements, challenges, and future potential. First, we examine the anatomy and key physiological properties of the cornea. This is followed by an exploration of 3D bioprinting modalities and selection of suitable bioinks for CTE, and in vivo assessment of bioprinted corneas. Subsequently, we provide a detailed analysis of current challenges associated with 3D bioprinting for the corneal tissue. Additionally, we review alternative emerging techniques other than bioprinting that contribute to the advancement of CTE. Finally, we address current and future frontiers, along with the clinical significance of artificial corneas.

## 2 Anatomy and Function of Human Cornea

**Figure 1:**
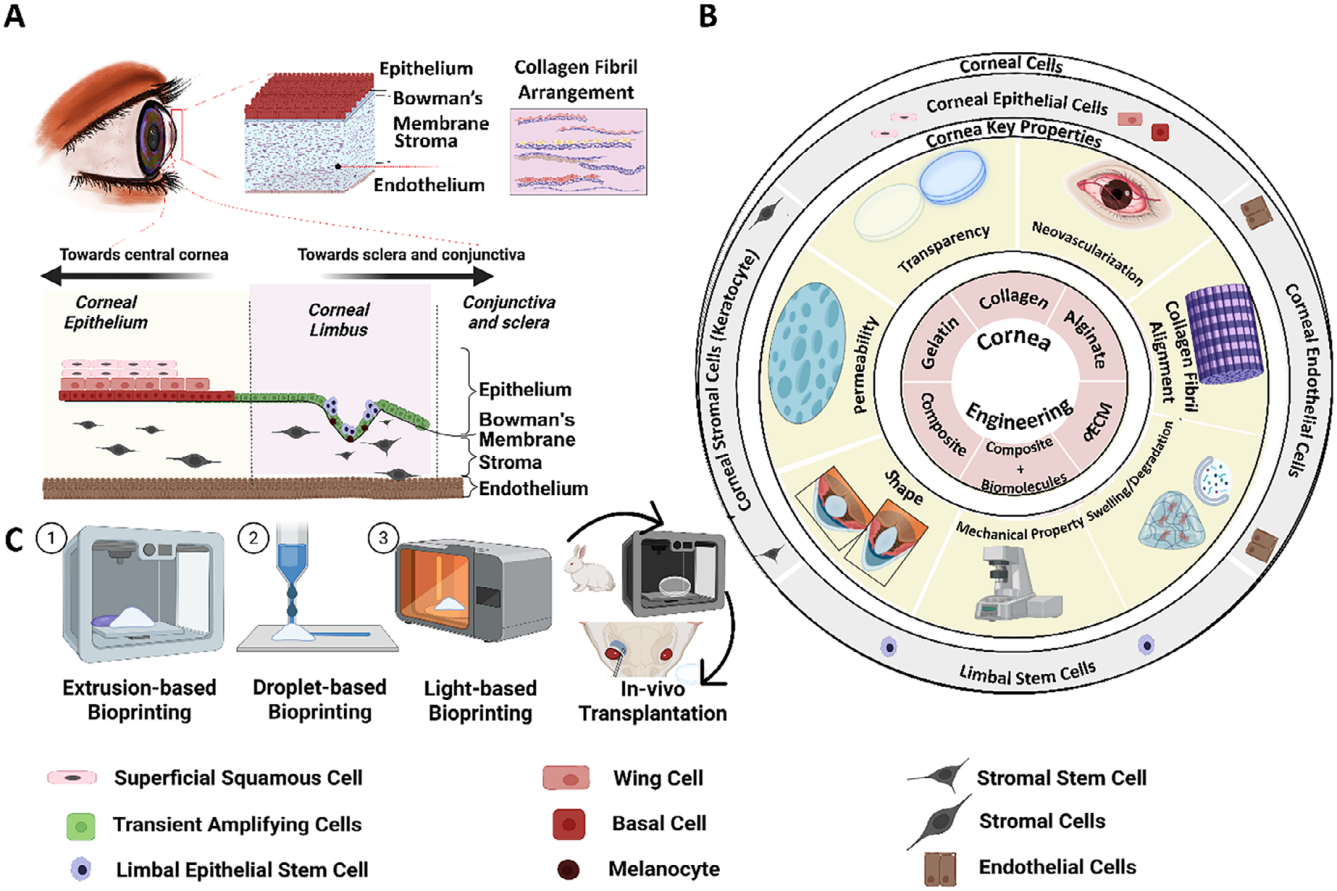
A) Schematics illustrating corneal cellular layers, including epithelium, stroma, endothelium, and collagen fibril arrangement in the stromal layer. Detailed representation of the corneal microenvironment near its periphery. Toward the middle of the cornea lies the corneal epithelium, while toward the sclera and conjunctiva are the corneal limbus, conjunctiva, and sclera. The corneal epithelium consists of superficial squamous, wing, and basal cells. Beneath the epithelial layer, the stromal layer contains stromal cells and stromal stem cells, with endothelial cells sequentially arranged next to each other to form a very thin layer known as the endothelium. B) Schematic representation of key factors in corneal engineering. The illustration highlights essential components of the cornea, including its cell types and its key physiological properties. Additionally, it outlines suitable bioinks for 3D bioprinting of cornea tissue. C) Schematic highlights bioprinting modalities and in vivo implantation of bioprinted corneal tissues. *Created with BioRender.com*.

The human cornea is a transparent, avascular, highly refractive anterior portion of the eye that encompasses the pupil, iris, and anterior chamber.^[^ ^27^ ^]^ As light enters the eye, it first encounters the cornea, which acts as the eye's primary refractive surface. The cornea is slightly thinner at the center (550 µm) than at the periphery (650 µm). Its curvature, thickness, and refractive index determine its refractivity and converging capacity at the retina by refraction of light and focusing.^[^ ^5^, ^28^, ^29^ ^]^ The central corneal thickness is 0.5 mm, which gradually increases toward the sclera.^[^ ^30^ ^]^ The biomechanical organization and external surroundings regulate corneal shape and curvature. The maintenance of corneal curvature primarily depends on the stiffness of the anterior corneal stroma.^[^ ^31^ ^]^ Additionally, the cornea protects the inner ocular structures from pathogens and particulate matter with the help of the sclera.^[^ ^32^ ^]^ The cornea also filters out ultraviolet (UV) radiation from the sun. It consists of three cellular layers: the corneal epithelium, stroma, and endothelium^[^ ^28^, ^33^, ^34^ ^]^ (**Figure** **1**A). Bowman's layer delineates epithelium and stroma, while Descemet's membrane provides a collagenous interface between stroma and endothelium.^[^ ^32^ ^]^ These layers are discussed in detail in the following sub‐sections.

### 2.1 Corneal Epithelium

The epithelial surface of the cornea, its outermost layer, serves as the primary barrier against external environmental factors. It plays a significant role in focusing light by forming an essential interface with the tear film.^[^ ^28^ ^]^ This layer consists of a stratified, non‐keratinizing squamous epithelium, extending uniformly from one edge of the limbus to the other, ensuring structural consistency across the entire corneal surface.^[^ ^34^ ^]^ Moreover, the layer plays a crucial role in maintaining transparency and stromal deturgescence by regulating the exchange of water and soluble substances into and out of the stroma.^[^ ^32^ ^]^ The corneal epithelium and tear film function synergistically to create a smooth anterior surface, essential for the cornea's refractive function. Upon blinking, the tear film interacts with the glycocalyx of human corneal epithelial cells (hCECs), which allows the tear film to spread uniformly throughout the cornea's surface.^[^ ^35^ ^]^

The epithelial layer is approximately 50 µm thick and consists of 5–6 layers of cells, including superficial, wing, and basal cells. The connection of epithelium to the basement membrane is vital for maintaining corneal integrity, and abnormalities in this relationship result in medical complications.

### 2.2 Corneal Stroma

The corneal stroma serves as the primary structural component, making up ≈80% of the cornea's thickness.^[^ ^34^, ^36^, ^37^ ^]^ The ECM, along with meticulously arranged collagen fibers, facilitates remarkable transparency. Collagen fibrils consist of organized bundles of fibers and are arranged in parallel layers, called lamellae. The collagen bundles within the lamellae are essential for providing necessary structural reinforcement against both tensile loads and shear forces.^[^ ^37^ ^]^ This well‐organized arrangement minimizes forward light scattering and enhances corneal transparency and mechanical stability.^[^ ^34^, ^38^ ^]^ The cellular component of corneal stroma is primarily composed of keratocytes, which are responsible for maintaining its structural integrity through the synthesis of collagen, glycosaminoglycans (GAGs), and matrix metalloproteinases (MMPs).^[^ ^34^ ^]^

### 2.3 Corneal Endothelium

The corneal endothelium consists of a monolayer of polygonal cells situated on the posterior side of the cornea. These cells play a crucial role in maintaining the dehydrated state of the stroma by utilizing ionic pumps found in their basolateral plasma membranes.^[^ ^33^ ^]^ Endothelial cell activity has been associated with maintaining the stroma's 78% water content.^[^ ^39^ ^]^ Dehydration involves a pump‐leak method, where the fluid is expelled from the corneal stroma into the aqueous humor. The corneal endothelium involves actin filaments, which are densely concentrated in peripheral bands at the apical region, enabling cell migration and shape maintenance.^[^ ^40^ ^]^

Adjacent to this layer is the Descemet's membrane, an acellular structure formed by the ECM secreted by corneal endothelial cells (CEnCs). While not entirely contiguous, this membrane lies next to the flattened endothelial cell layer, contributing to the structural and functional integrity of the cornea.^[^ ^41^ ^]^ CEnCs are identifiable in vivo by a lack of mitotic activity. However, it should be noted that individuals inherit an adequate number of such cells at birth. An individual's initial cellular density at birth is generally ≈3,500 cells mm^−2^, and it gradually declines throughout every year by ≈0.6% in normal conditions.^[^ ^42^, ^43^ ^]^ However, in pathological conditions, such as in the case of Fuchs Endothelial Dystrophy, there is an accelerated loss of CEnCs, leading to corneal blindness over time.

## 3 3D Bioprinting of the Cornea

The development of 3D printing began in the 1980s with the introduction of stereolithography (SLA), which involves the incremental formation of solid objects using a liquid photocurable resin.^[^ ^44^, ^45^, ^46^ ^]^ Bioprinting technologies have rapidly evolved since then. Extrusion‐based bioprinting (EBB) emerged in the early 2000s, utilizing bioinks composed of cells and hydrogels to fabricate intricate tissue‐like structures.^[^ ^47^ ^]^ Laser‐based bioprinting methods, such as laser‐assisted bioprinting, have also been developed, achieving high‐resolution bioprinting and cell encapsulation within 3D structures.^[^ ^48^ ^]^ A significant advancement in bioprinting is the development of multi‐material bioprinters, which facilitate the creation of heterocellular structures with precise spatial manipulation. Recent advancements in 3D bioprinting have integrated additional elements, such as vascularization and sensors, into bioprinted tissues to replicate physiological environments and enable sensing capabilities.^[^ ^49^ ^]^ To bioprint corneal tissue, it is imperative to comprehend the biological and physiological properties of the cornea. These properties guide the formulation of bioinks, the selection of bioprinting modalities, and the optimization of bioprinting parameters (Figure 1B). The following sections discuss key corneal properties for bioprinting, different bioprinting modalities, biomaterials for developing relevant bioinks for CTE, assessment of bioprinted corneal tissues, and challenges in bioprinting of the cornea.

### 3.1 Key Physiological Properties for Corneal Tissue Engineering

The cornea's stress response is influenced by the intricate organization of its constituent layers and structural subcomponents, which collectively define the physiological and biological properties of the cornea, such as transparency, biomechanical properties, neovascularization, swelling, degradation, collagen fibril alignment, and permeability (Figure 1B). Corneal transparency depends on the precise alignment and spacing of collagen fibrils, as well as endothelial regulation of hydration.^[^ ^50^ ^]^ Corneal biomechanics considers ocular pressure, corneal rigidity, and viscoelasticity.^[^ ^51^ ^]^ Neovascularization disrupts the cornea's avascular nature, compromising clarity.^[^ ^52^ ^]^ Swelling, driven by permeability changes or enzymatic activity, weakens structural integrity and impairs transparency.^[^ ^53^, ^54^ ^]^ The corneal shape, critical for light refraction, is maintained by its biomechanical properties and ECM organization.^[^ ^55^ ^]^

#### 3.1.1 Transparency

**Figure 2:**
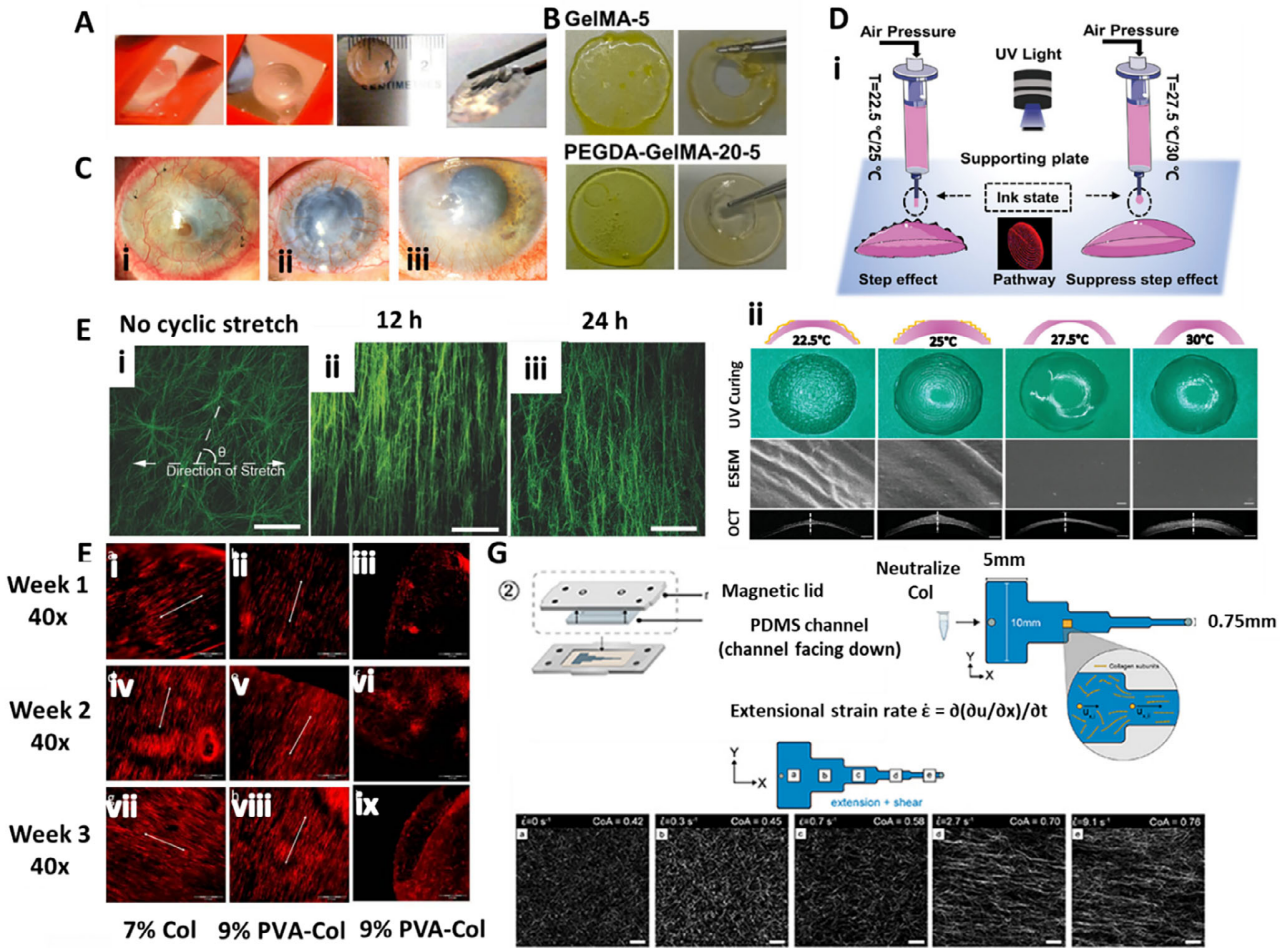
A) Images showed transparency of a 3D bioprinted GelMA construct matching the dimensions of the human corneal stroma (adapted and reproduced with permission from ref. [59] 2020, Biomedical Engineering Society). B) Photographs displayed transparency of printed hydrogel discs: GelMA‐5 and PEGDA‐GelMA‐20‐5 (adapted and reproduced with permission from ref. [61] 2022, Elsevier B.V. on behalf of KeAi Communications Co. Ltd.). C) Corneal neovascularization was observed in different conditions: i) autoimmune disease, ii) after penetrating keratoplasty, and iii) following post‐inflammatory responses (adapted and reproduced with permission from ref. [102] 2024, MDPI, Basel, Switzerland). D) i) A schematic illustration depicted the temperature‐controlled printing process designed to minimize the step effect. ii) Images captured ink deposition at varying extrusion temperatures and detailed surface features of printed corneal implants. Bright‐field imaging during UV curing revealed the structural integrity of the printed cornea (scale bar = 200 µm). Environmental scanning electron microscopy (ESEM) provided micro surface visualization (scale bar = 200 nm). Optical coherence tomography (OCT) presented corneal structures printed at different temperatures (scale bar = 1 mm), (adapted and reproduced with permission from ref. [23] 2023, Wiley‐VCH GmbH). E) Alignment of collagen nanofibers was analyzed in different conditions: i) without cyclic stretch and ii, iii) under cyclic stretching at one cycle per minute (adapted and reproduced with permission from ref. [18] 2016, WILEY‐VCH Verlag GmbH & Co. KGaA, Weinheim). F) Cell orientation changed depending on fibril orientation, red fluorescent protein‐labeled human keratocytes were cultured on various electrospun scaffolds: (i, iv, vii) 7% aligned collagen; (ii, v, viii) 9% PVA‐COL aligned; and (iii, vi, ix) non‐aligned scaffolds. Cultures were maintained for one week (a–c), two weeks (d–f), and three weeks (g–i). Scale bar = 500 µm, (adapted and reproduced with permission from ref. [106] 2018, MDPI, Basel, Switzerland). G) A schematic representation illustrated the two‐piece channel fabrication and its modular base for collagen fibril alignment. A top‐view schematic depicted a 25 mm‐long segmented microfluidic channel designed to induce extensional flow. As the cross‐sectional area decreased, COL1 subunits experienced extensional flow, with a strain rate determined by the velocity change along the flow direction (left to right) at each constriction's entrance (adapted and reproduced with permission from ref. [107] 2022, IOP Publishing Ltd).

Corneal transparency can be defined as the cornea's capability for light transmission. Transparency is governed by stromal thickness, collagen fibril arrangement, the integrity of the epithelial barrier, and the proper function of the endothelium, which maintains the hydraulic balance in the native cornea.^[^ ^54^ ^]^ Maintaining corneal transparency contributes to eye health and optimal visual function. The quantification of corneal transparency is achieved by evaluating the transmittance of light through the cornea. According to a subjective grading scale (1–4), a score of 0 indicates no opacity, while a score of four signifies nearly the entire corneal surface being clouded, obstructing visualization due to opacity.^[^ ^56^ ^]^ Light transmission in the native cornea is quantified as a percentage, with the cornea exhibiting 80–94% light transmission at wavelengths of 450–600 nm and 95–98% light transmission at wavelengths of 600–1,000 nm.^[^ ^57^ ^]^ The absorption of UVB and UVC radiation by proteins and vitamins within the corneal epithelium and stroma plays a protective role for the eyes. This absorption significantly reduces the transmission of radiation within these wavelength ranges, thereby minimizing potential cellular damage. However, the transmission increases to 80% at 380 nm and surpasses 90% within the range of 500–1,300 nm.^[^ ^58^ ^]^ Mahdavi et al. recorded the transmittance of gelatin methacrylate (GelMA) for corneal constructs at 400–1,000 nm and found that 12.5% GelMA achieved an 80–95% transmission at the edge and 78–90% transmittance at the center, which was closer to that of the native cornea (**Figure** **2**A).^[^ ^59^ ^]^ Although 12.5% GelMA scaffolds initially exhibited optical properties comparable to the native cornea, transparency declined over time after cell encapsulation due to proliferation and light scattering. In addition, the absorption of eosin Y in the blue spectrum may further limit long‐term visual performance in vivo, highlighting the need for optimized photoinitiator systems and extended validation. Similarly, Bektas et al. obtained higher than 80% transmittance for 15% GelMA at (250–700 nm) except cell‐loaded slabs on Day 1.^[^ ^60^ ^]^ While the scaffolds demonstrated favorable transparency, mechanical stability, in vitro cytocompatibility, and comprehensive in vivo studies are still required to confirm their long‐term functionality and integration within the corneal environment. Zhang et al. demonstrated that a combination of decellularized extracellular matrix (dECM)/GelMA (10%) had superior transmittance with ≈90% with 10% GelMA at 780 nm.^[^ ^25^ ^]^ Interestingly, the transmittance was increased with the presence of human corneal fibroblasts by ≈14%. Although dECM/GelMA hydrogels showed promising short‐term transparency, their light transmittance was lower than pure GelMA (≤≈86% above 500 nm, reduced in the blue‐violet range), and long‐term optical clarity under physiological conditions remains untested. Moreover, the printed corneas were thicker than native tissue (≈900 vs. 500–550 µm), which may alter refractive performance, yet no in vivo wavefront or refractive outcomes were evaluated. Furthermore, He et al. reported that a lower concentration of 10% poly (ethylene glycol) diacrylate (PEGDA) in PEGDA/GelMA had higher transparency (≈91%) at 600 nm compared to higher concentrations.^[^ ^61^ ^]^ This transparency level surpassed that of the native cornea, highlighting the potential of PEGDA/GelMA hydrogels for CTE applications (Figure 2B). However, PEGDA–GelMA hydrogels achieved higher light transmittance than native cornea, yet transparency declined at higher PEGDA content due to increased light scattering, highlighting the need to optimize PEGDA levels for optical performance. In another study, Kutlehria et al. formulated a bioink consisting of gelatin (4%), sodium alginate (3.25%), and collagen (5%), and the bioprinted constructs achieved 75–90% transmittance in the range of 400–700 nm.^[^ ^21^ ^]^ Although in vitro transmittance reached >75%, the optical clarity under physiological conditions remains untested. Thus, in vivo studies evaluating long‐term transparency, tissue integration, and refractive outcomes are required for translation. In yet another study, Zhang et al. used a combination of sodium alginate (0.02 g mL^−1^) and gelatin (0.10 g mL^−1^), which achieved 85–94% transmittance in the visible spectrum.^[^ ^62^ ^]^ This study also demonstrated that bioprinted constructs had the potential to shield tissues from radiation due to their lower UV band transmittance.

Clinically, maintaining corneal transparency is critical for corneal substitutes. UVA/riboflavin cornea cross‐linking, despite creating bonds within the stroma, largely preserves optical clarity in the long term because it does not significantly alter collagen interfibrillar spacing.^[^ ^63^ ^]^ Clinically, transient stromal haze may occur in the first weeks of the postoperative period, typically attributed to keratocyte apoptosis and repair processes, but the cornea typically recovers its full transparency over ensuing months as the stroma remodels and repopulates.^[^ ^64^ ^]^ In edematous corneas, such as those affected by bullous keratopathy, corneal cross‐linking may induce a transient improvement in transparency by compacting the swollen stromal lamellae. However, this effect is temporary unless the underlying endothelial pump dysfunction is addressed.^[^ ^64^ ^]^ Bioengineered cross‐linked corneal implants can also achieve high optical performance when designed appropriately. Enzymatically cross‐linked protein hydrogels (using HRP or transglutaminase) form relatively uniform polymer networks that minimize light scattering and can achieve transparency comparable to native corneal tissue.^[^ ^65^ ^]^ For instance, collagen or silk fibroin hydrogels cross‐linked via HRP/H_2_O_2_ retain optical transparency in vitro comparable to the normal cornea.^[^ ^65^ ^]^ On the other hand, certain chemical cross‐linkers, such as glutaraldehyde, can introduce transient coloring or haze to collagen scaffolds due to polymerized aldehyde residues, resulting in a yellowish hue.^[^ ^66^ ^]^ Similarly, photosensitizers like riboflavin or rose Bengal can initially cause stromal light scatter. These optical side‐effects tend to diminish over time with proper post‐processing techniques, such as washing or quenching, which remove residual cross‐linking agents and eliminate haze or chromophores without compromising mechanical integrity.^[^ ^66^ ^]^ For example, scaffolds double‐cross‐linked with EDC and glutaraldehyde, when treated with quenching agents to neutralize free aldehydes, remained as clear as controls.^[^ ^66^ ^]^ In vivo, early reductions in transparency (from edema, cell loss, or inflammation) typically resolve as the graft integrates. Ultimately, successful cross‐linking protocols yield corneal tissues that are both strong and transparent, fulfilling the cornea's role as the eye's main refractive window.^[^ ^66^ ^]^

Most bioprinting studies on corneal stromal transparency have achieved levels comparable to, or even exceeding, those of the native cornea. However, creating a fully functional cornea requires incorporating additional layers, including acellular and cellular layers, to replicate the entire structure. However, the inclusion of these layers, primarily cell‐based, can significantly influence transparency. Specifically, poorly differentiated cells and abnormal multilayering can increase light scattering and reduce transparency. Conversely, a smooth, confluent, and well‐differentiated layer of epithelial cells can maintain or even enhance transparency by minimizing light scattering at the surface. Similarly, controlled ECM deposition by cells plays a critical role in maintaining transparency. While organized ECM deposition supports transparency, excessive or misaligned ECM, cellular debris, or disorganized structures can lead to increased light scattering and diminished optical clarity. Moreover, higher cell density in certain regions of the cornea can impact light transmission. In this context, 3D bioprinting offers a powerful tool for controlling cell deposition, including cell density and spatial arrangement. This precise organization not only supports optimal light transmission but can also positively influence cell differentiation and tissue regeneration, contributing to the overall functionality of the artificial cornea.

#### 3.1.2 Mechanical Characteristics

Mechanical properties play a pivotal role in determining the cornea's shape and functions. Understanding how the mechanical properties influence the cornea's shape has gained significance, due to a growing number of refractive surgeries since 90s.^[^ ^67^ ^]^ Vision can be significantly affected by alterations in mechanical characteristics resulting from disease, trauma, or surgery.^[^ ^23^ ^]^ The primary factors affecting corneal biomechanics include thickness, curvature, and the organization of collagen fibers. Among these, corneal thickness plays a pivotal role (≈50 µm for the epithelium, 500 µm for the stroma, and 5–10 µm for the endothelium) in defining its biomechanical characteristics. Thinner corneas tend to exhibit reduced elasticity and are susceptible to deformation when exposed to external forces, while thicker corneas have generally higher rigidity and greater resistance to deformation.^[^ ^55^ ^]^ Furthermore, a thinner central cornea can increase glaucoma risk by affecting intraocular pressure (IOP) readings, potentially delaying diagnosis, and leading to more advanced disease stages.^[^ ^68^, ^69^ ^]^ According to a study, a difference of 10 µm in central corneal thickness can result in a change of 0.18 to 0.49 mmHg in IOP.^[^ ^70^ ^]^ Moreover, studies have demonstrated that changes in the curvature of the cornea can affect its biomechanical reaction. For example, compared to a steeper cornea, a flatter cornea might be prone to deformation.^[^ ^71^, ^72^, ^73^ ^]^

The viscoelastic properties of the cornea are crucial in defining its mechanical behavior, resulting from the interplay of various structural components, including collagen fibers and a gel‐like matrix composed of polyanionic, hydrophilic ground substance. As a viscoelastic material, the cornea exhibits both elastic (recoverable) and viscous (time‐dependent) responses to mechanical stresses. This dual behavior allows the cornea to deform under applied forces, such as IOP and external mechanical loads, and to gradually return to its original shape, thereby ensuring structural resilience.^[^ ^55^ ^]^ Collagen within the corneal ECM provides elasticity to the cornea. The diameter, spacing, and orientation of collagen fibrils significantly influence corneal biomechanics.^[^ ^36^ ^]^ Simultaneously, viscoelastic behavior, attributed to the tensile properties of collagen, enables the cornea to absorb and dissipate mechanical energy effectively. This interplay ensures that the cornea maintains its shape and optical clarity while adapting to dynamic forces, highlighting the importance of collagen microstructure in both its mechanical strength and viscoelasticity.^[^ ^69^, ^74^, ^75^, ^76^ ^]^

The average tensile strength of the human cornea is 3.81±0.40 MPa.^[^ ^77^ ^]^ Ulag et al. reported that 13% poly (vinyl alcohol) (PVA) bioprinted construct showed a tensile stress of ≈40 MPa, while 13% PVA/5% chitosan constructs showed ≈9 MPa.^[^ ^78^ ^]^ Even though there is no comparison with the native cornea as a control group, the tensile stress of the constructs was higher than that of the native cornea. Although these values suggest enhanced mechanical robustness, no direct comparison with the mechanical properties of native corneal tissue was provided in the study. This missing comparison makes it difficult to assess the physiological relevance of such high‐strength corneal substitutes. This is because constructs stiffer than the native cornea may compromise biological integration, which is equally critical for corneal function. Corneal cells, keratocytes, are highly sensitive to substrate stiffness. In their native, compliant ECM, keratocytes remain quiescent. If the environment becomes abnormally stiff, these cells can transform into a repair phenotype (fibroblasts/myofibroblasts) that produces scar tissue. This phenomenon is well‐documented in corneal fibrosis, where post‐injury to stroma often stiffens due to disordered ECM deposition, and promotes transforming growth factor‐beta 1 (TGF‐β1)‐driven keratocyte‐to‐myofibroblast transformation.^[^ ^79^ ^]^ Additionally, some studies report bulk tensile or compressive properties, which are often performed under static and unidirectional loading conditions. The significance of these test results falls short against the native cornea's depth‐dependent, anisotropic, and viscoelastic mechanical behavior.^[^ ^80^ ^]^ Although high mechanical strength corneal constructs have been reported for CTE, insights from commercial artificial corneas (keratoprostheses) illustrate the risks associated with mechanical mismatch. Traditional KPro devices fabricated with rigid materials, like polymethyl methacrylate, possess tensile stiffness far exceeding compared of the native cornea. While PMMA itself is biologically inert, its lack of flexibility and poor tissue adhesion often lead to integration failure at the junction between the device and host tissue.^[^ ^81^ ^]^ The high stiffness of the cross‐linked anterior stroma causes initial keratocyte apoptosis (cell death) in that region and stimulates an inflammatory cascade. Clinically, this manifests as transient stromal haze in the months after corneal collagen cross‐linking.^[^ ^82^ ^]^ These observations underscore the importance of designing corneal substitutes with biomechanical properties that closely mimic the native tissue to support both structural function and biological integration.

Moreover, hydration also has a pronounced effect on the mechanical behavior of hydrogel‐based materials. For example, studies demonstrated that hydrogels exhibit significantly reduced stiffness when fully hydrated compared to their dry or partially hydrated states, emphasizing the need to evaluate mechanical performance under physiologically relevant moisture conditions.^[^ ^83^, ^84^ ^]^ Recognizing that hydration affects mechanical properties, several studies pre‐hydrate their samples before conducting mechanical tests. For example, in one study, hydrogels soaked in phosphate‐buffered saline (PBS) at 37 °C for 2 h still exhibited high tensile, compressive, and suture‐retention strength.^[^ ^85^ ^]^ Another study evaluated constructs after longer PBS incubations (up to 21 days), with tensile strength reaching 3.42 ± 0.22 MPa and maintaining over 2.1 MPa after 3 weeks.^[^ ^17^ ^]^ In another study, Campos et al. determined the compressive modulus of a bioink with 0.5% agarose and 0.2% type I collagen, before cross‐linking as ≈18 kPa.^[^ ^86^ ^]^ However, it remains lower than the compressive modulus of the native human corneal stroma, which is reported ≈38 kPa as an average compressive modulus for full thickness, but it also differs depending on the anatomical depth.^[^ ^87^ ^]^ Zhang et al. reported that an increased concentration of dECM (derived from porcine cornea) to 1% (w/v) increased the compressive strength of constructs up to ≈74 kPa.^[^ ^25^ ^]^ This could primarily be attributed to enhanced intermolecular interactions and cross‐linking within the composite bioink. ECM components, including collagen, GAGs, and other matrix proteins, may provide additional mechanical reinforcement by facilitating denser network formation, improved structural integrity, and greater resistance to compressive forces. As per the literature, the Young's modulus of the cornea is 0.27 MPa at age 27 and increases to 0.52 MPa by age 100.^[^ ^88^ ^]^ Additionally, the in vitro determination of the corneal Young's modulus varies significantly, ranging from 0.1 to 57 MPa, likely due to differing experimental conditions.^[^ ^76^ ^]^ Moreover, Young's modulus of constructs with ECM was ≈27 kPa, representing an increase of ≈33% compared to constructs without ECM. This enhancement brings the Young's modulus of the constructs closer to the lower end of the native cornea's range of values.

Despite these improvements, there is still a need for materials that enhance mechanical properties alongside other key characteristics. Several approaches can improve these properties. For instance, granular microgels can help in improving the mechanical properties of the constructs whilst maintaining transparency. One promising example involves the use of small uniform granular microgels, which showed improved bioprintability while preserving optical clarity.^[^ ^89^ ^]^ In the study, granular polyrotaxane (GPR) microgels enhanced the mechanical properties of constructs by forming a densely packed and mechanically interlocked network through microgel jamming and α‐cyclodextrin (αCD)‐mediated supramolecular cross‐linking. Additionally, microgels' granular nature offers self‐healing and rapid shape recovery post‐injection, resulting in stronger, more resilient, and easily injectable material as compared to traditional corneal biomaterials.^[^ ^89^ ^]^

Cross‐linking methods, including covalent, ionic, and dynamic cross‐linking, can boost strength, stiffness, self‐healing, and resilience. Clinical corneal collagen cross‐linking with UVA/riboflavin has been extensively validated to significantly increase stromal stiffness, halting the progression of ectatic diseases by reinforcing the cornea.^[^ ^90^ ^]^ Mechanical testing showed that corneas treated with the full Dresden corneal cross‐linking protocol (riboflavin + dextran + UVA) exhibited a ≈28 ± 17% increase in tangent modulus compared to corneas treated with dextran only, indicating that the stiffening effect arises specifically from UVA‐activated cross‐linking rather than osmotic dehydration alone.^[^ ^63^ ^]^ Analogous bioengineered cross‐linking methods used in CTE similarly demonstrated enhancements in mechanical strength. Enzymatic cross‐linkers like transglutaminase catalyze the formation of covalent bonds between collagen molecules (mimicking the natural lysyl oxidase‐mediated cross‐linking in vivo) and have been shown to stiffen collagen constructs.^[^ ^91^ ^]^ For example, transglutaminase‐induced cross‐linking in collagen hydrogels and corneas can increase mechanical rigidity without the need for UV light, an approach tested in rabbit models.^[^ ^92^ ^]^ At 1.5 MPa stress, transglutaminase cross‐linking increased corneal stiffness ≈1.5‐fold compared to UVA/riboflavin (29.8 vs. 20.5 MPa).^[^ ^92^ ^]^ Likewise, horseradish peroxidase (HRP) with H_2_O_2_ can induce dityrosine cross‐linking in protein‐based scaffolds, yielding hydrogels that are both mechanically tunable and elastic.^[^ ^65^ ^]^ These cross‐linking strategies provide immediate improvement in graft mechanical stability and suturability, and their effects tend to persist long‐term due to the permanence of the induced collagen cross‐links.^[^ ^66^ ^]^ In fact, long‐term studies of UVA/riboflavin cornea cross‐linking in vivo confirm that the cornea remains biomechanically strengthened years after treatment, with slight flattening of curvature as the tissue adapts.^[^ ^93^ ^]^ Importantly, while increased cross‐linking density is generally beneficial for strength and enzymatic resistance, excessive cross‐linking may lead to matrix compaction or depletion of lysine residues critical for cell adhesion.^[^ ^91^ ^]^ Thus, an optimal cross‐linking degree is sought to ensure strong yet biologically accommodating grafts. Further, recent studies highlighted how strategically engineered cross‐linking methods, including covalent, ionic, and dynamic cross‐linking, can boost the strength, stiffness, self‐healing, and resilience of corneal biomaterials. Rizwan et al. demonstrated that sequential physical and UV covalent cross‐linking of GelMA produced an eightfold increase in mechanical strength, allowing constructs to withstand surgical forces without breaking.^[^ ^94^ ^]^ However, such hybrid systems aimed to increase stiffness may compromise their flexibility. In contrast, Wei et al. employed reversible Schiff‐base bonds for dynamic self‐healing hydrogels, achieving tunable strength and rapid mechanical recovery.^[^ ^95^ ^]^ These gels also exhibited strong adhesion properties to native cornea as well as therapeutic benefits in vivo. Oxidized gellan gum (2.0 wt.%)/carboxymethyl chitosan (1.5 wt.%) formulation exhibited the highest tissue adhesion (33.5 kPa) and shear strength (7.1 kPa), representing 60–70% improvement compared to the 1% formulation. This enhancement is due to a higher oxidized gellan gum concentration, which provided more aldehyde groups, forming more Schiff‐base bonds with tissue amino groups, thereby strengthening adhesion. Similarly, Li et al. engineered a mechanically robust and optically transparent patch using a multi‐modal cross‐linking strategy comprising UV‐induced polymerization, dynamic Schiff‐base chemistry, and UVA/riboflavin‐mediated clinical corneal cross‐linking.^[^ ^96^ ^]^ This dual‐network architecture significantly outperformed single‐cross‐linked (UV‐only) hydrogels in terms of burst pressure and tensile strain. Tensile strain reached 500–600% for the GelMA + Pluronic F127 diacrylate (F127DA) + aldehyde‐functionalized Pluronic F127 (AF127) + 3% collagen type I formulation (G8F4C3), compared to <50% for GelMA‐only (G12) and ≈120% for GelMA + F127DA (G8F4). These improvements arose from the synergistic contributions of covalent cross‐links for structural integrity, dynamic Schiff‐base bonds for energy dissipation and self‐healing (tensile elongation increased from ≈120% in G8F4 to ≈500–600% in G8F4C3) and UVA/riboflavin cross‐linking for enhanced tissue adhesion (burst pressure >400 mmHg ≈ 53 kPa for G8F4C3 with UVA/riboflavin and ≈20 kPa for typical UV‐only GelMA hydrogels, lap shear strength was also highest in G8F4C3 with UVA/riboflavin) and biomechanical compatibility (tensile modulus increased to ≈60 kPa in G8F4C3 with UVA/riboflavin and ≈40 kPa in G8F4, compressive modulus improved with no damage even at high strain, unlike UV‐only GelMA which failed before 50% strain). These complementary mechanisms collectively result in significantly superior mechanical strength, elasticity, toughness, and resilience, highlighting the cross‐linking design as a powerful lever for engineering advanced corneal constructs. Adding chemical groups like catechols can enhance adhesion and toughness.^[^ ^97^ ^]^ These strategies provide effective ways to strengthen materials without compromising essential properties. Bioprinting can enhance the mechanical properties of artificial corneas by enabling precise control over material composition and structure.^[^ ^98^ ^]^ Through bioprinting, materials can be precisely deposited to mimic the natural layered architecture of the native cornea. This technique allows for meticulous control over the characteristics of each layer within the constructs. The precision of bioprinting also enables the integration of micro or macro fibers into the hydrogel matrix, improving load‐bearing capacity and mechanical anisotropy.^[^ ^99^, ^100^, ^101^ ^]^ Furthermore, bioprinting can enhance the alignment of cellular structures within the material, optimizing the mechanical properties needed for long‐term corneal function. Therefore, bioprinting offers a powerful tool to design and fabricate artificial corneas with superior mechanical properties tailored to mimic natural corneal tissue.

#### 3.1.3 Neovascularization

Corneal neovascularization is characterized by the infiltration of new blood vessels into the cornea from the limbus. This phenomenon arises due to a disturbance in the balance among pro‐angiogenic factors, such as vascular endothelial growth factor (VEGF), fibroblast growth factor (FGF), and angiopoietins, and anti‐angiogenic factors like pigment epithelium‐derived factor (PEDF), thrombospondins, and endostatin^[^ ^102^ ^]^ (Figure 2C). This balance is crucial for maintaining corneal transparency by preventing unwanted blood vessel growth into the normally avascular cornea. Immature neovascularization can result in loss of lipids, chronic inflammation, and formation of scar tissue, which creates a risk to the transparency of the cornea and visual acuity.^[^ ^103^ ^]^ For 3D bioprinted corneal grafts, neovascularization is one of the important characterizations for the construct's success. For instance, an implanted PEGDA/GelMA hydrogel did not cause any inflammation and neovascularization in adult male rabbits.^[^ ^61^ ^]^ However, Bektas et al. observed deep vascularization on the first week post‐implantation of GelMA into a mid‐stromal pocket in the cornea.^[^ ^104^ ^]^ After treating with a single dose of sub‐conjunctival anti‐vascular endothelial growth factor (VEGF) drug, they observed that neovascularization decreased remarkably, and the cornea recovered its transparency. In another study, researchers did not observe any VEGF secretion in scaffolds composed of 80% nanocellulose and 20% alginate, which were supplemented with mesenchymal stem cell‐secreted PEDF. This finding suggests that PEDF may act as a potential down regulator of VEGF.^[^ ^105^ ^]^

#### 3.1.4 Swelling and Degradation

Corneal swelling, or edema, can occur due to many reasons, such as injury, inflammation, or infection. This swelling causes an increase in corneal thickness,^[^ ^108^ ^]^ which can adversely affect the tensile and compressive properties of the tissue, thereby impairing the ocular functions and potentially leading to blurry vision.^[^ ^109^, ^110^, ^111^ ^]^ Edema can also result from fluid accumulation, which can indicate inflammation. However, it can also occur without inflammation, such as in cases of endothelial dysfunction or increased IOP. The endothelial layer of the cornea is responsible for pumping liquid out of the cornea. Endothelium malfunction can lead to edema, resulting in blurry vision due to many reasons, such as injury, inflammation, or infection.

From a corneal regeneration perspective, hydrogels are commonly used biomaterials in implants due to their similarity to the cornea, particularly their high‐water content, with an equilibrium water content of ≈80%.^[^ ^112^ ^]^ However, hydrogels have water‐holding capacity; therefore, the issue of swelling arises in artificial hydrogel‐based corneal constructs. Hence, the swelling property of artificial corneas made from hydrogels is a critical parameter to evaluate before transplantation. Hydrogels reach equilibrium by either absorbing or releasing their solvent in response to various stimuli. The equilibrium swelling of a gel is affected by the interactions between the polymer network and solvent.^[^ ^113^ ^]^ Zhang et al. compared the swelling properties of corneal dECM with a dECM/GelMA composite.^[^ ^25^ ^]^ The swelling rate of dECM was high within the first 2 h, showing a further decrease from 3 to 4 h before reaching a stable equilibrium state. However, the dECM/GelMA composite exhibited a significantly higher swelling rate. Moreover, GelMA alone achieved an equilibrium water content of ≈85% while dECM/GelMA reached 82%, comparable to ≈78% of the native cornea. Another study reported that saline absorption was rapid in chitosan compared to PVA due to the porous structure created by chitosan. Moreover, the degree of swelling increased by increasing chitosan content in the chitosan/PVA composite,^[^ ^113^ ^]^ and the maximum swelling was achieved by 13% PVA/5% chitosan composition. Mörö et al. observed water absorption by HA‐based printed constructs within 3 h, followed by a decrease in weight, resulting from leaching of free HA, until Day 4, with no further weight loss observed until Day 14.^[^ ^114^ ^]^

Degradation of corneal implants in the human body occurs because of the breakdown of the constituent biomaterials. Degradation is essential as it enables the newly formed tissue to replace the implanted graft, ensuring graft's integration with the surrounding biological environment and facilitating in vivo tissue regeneration and repair. Importantly, for efficient healing, the rate of new tissue formation and degradation of the implant should be synchronous. The epithelial layer heals within 7–14 days, requiring materials that degrade within the same period to support migration and barrier formation.^[^ ^115^ ^]^ The stromal layer takes months for remodeling, necessitating scaffolds that degrade over a similar timeframe for structural stability.^[^ ^116^ ^]^ The endothelial layer recovers from weeks to months, so materials should degrade accordingly to aid cell migration without disrupting aqueous humor balance.^[^ ^117^ ^]^ Researchers analyzed the degradation of GelMA constructs during 3 weeks of incubation in PBS and observed only 8% loss of the initial weight.^[^ ^60^ ^]^ High stability of constructs was achieved by dual cross‐linking of the gel, which was obtained by incubating the gel at 4 °C before UV cross‐linking, resulting in less degradation. In another study, Zhang et al. found that a hydrogel made of corneal ECM and GelMA retained ≈83% of its weight after 4 h of air exposure, whereas pure GelMA decreased to 69%.^[^ ^25^ ^]^ According to Ulag et al., the degradation rate of all samples, including those composed of chitosan and the chitosan/PVA composite, decreased after the initial stage, when the constructs absorbed the water highly.^[^ ^113^ ^]^ This reduction in degradation rate was attributed to the constructs initially absorbing water and subsequently losing their water content during the 3‐day incubation period. Additionally, the presence of chitosan increased the degradation rate, likely due to its porous structure, which enhanced water absorption.

#### 3.1.5 Shape

The cornea possesses a non‐spherical, dome shape, exhibiting a curved structure. The corneal shape is commonly characterized by its central steepness and gradual flattening toward the periphery. The mean corneal curvature is ≈43.00 diopters,^[^ ^118^ ^]^ but there can be variations among individuals, and the curvature may differ in the horizontal and vertical meridians. Anomalies in the shape of the cornea, such as those seen in astigmatism or keratoconus, can result in blurred vision. Having an ideal corneal shape in engineered constructs is important for accurate focusing of light onto the retina, resulting in clear and precise vision.^[^ ^119^ ^]^ Ectatic disease, progressive thinning and bulging of the cornea, is correlated with the corneal shape, which is anisotropic and has varying cohesive strength in different peripheries. Cell behavior is affected by environmental signaling, which includes biochemical cues, such as curvature.^[^ ^23^ ^]^ A study using polydimethylsiloxane (PDMS) demonstrated that the curvature affects cell morphology, orientation, and differentiation.^[^ ^120^, ^121^ ^]^ Nevertheless, a comprehensive understanding of the effects of corneal implant curvature on epithelial layer behavior and subsequent corneal regeneration remains elusive. Further research is required to elucidate the intricate interactions between implant‐induced curvature changes and epithelial cell dynamics, which are critical for successful corneal regeneration.

3D Bioprinting plays a role in precisely shaping artificial corneas by enabling controlled deposition of bioinks to replicate the natural curvature and anisotropic structure of the cornea. Unlike traditional methods, which struggle to achieve the cornea's gradual flattening from the center to the periphery,^[^ ^17^ ^]^ bioprinting allows for precise geometric control by layering biomaterials in a spatially defined manner.^[^ ^122^ ^]^ Advanced imaging techniques, such as optical coherence tomography (OCT), can be integrated into the design process to create patient‐specific constructs that match individual corneal curvatures.^[^ ^123^, ^124^ ^]^ Additionally, bioprinting can generate region‐specific thickness gradients, mimicking the mechanical properties that contribute to the cornea's shape stability. Using bioinks with tunable mechanical properties and cross‐linking strategies, bioprinted constructs can maintain their dome‐like shapes. Furthermore, enhancing the bioprinting process to enable the alignment of collagen fibers during bioprinting significantly increases structural integrity. This improvement ensures that the artificial cornea maintains its curvature while effectively supporting epithelial cell attachment and proliferation. This precise control over shape and biomechanics makes 3D bioprinting a promising approach for developing artificial corneas that closely replicate the natural structure, optimizing light refraction, and visual function (Figure 2D). In a study, Xu et al. demonstrated epithelial cell behavior on four different regions of the convex‐shaped bioprinted cornea.^[^ ^23^ ^]^ Steeper slope gradient surfaces accelerated cell attachment, chromatin condensation, and epithelization compared to flat regions. Hence, consideration of the curvature of a cornea with varying thickness is crucial while designing artificial corneas.

#### 3.1.6 Alignment of Collagen Fibrils in Corneal Stroma

The microstructure of the cornea is essential for its proper function. In the posterior part of the cornea, fibrils are oriented toward the rectus muscle, whereas the anterior part has an isotropic distribution. Collagen fiber (lamella) orientation in the limbus part enhances the stiffness of the cornea and provides curvature, which is essential for focusing.^[^ ^125^ ^]^ Hence, corneal optical and biomechanical characteristics are affected by the network of collagen fibrils in the stroma. Therefore, a well‐structured implant, which has collagen fibrils aligned within the stroma, is needed for corneal tissue regeneration. The cornea remains transparent due to the highly organized, lattice‐like arrangement of collagen fibrils in the stroma. This uniform spacing prevents light scattering, allowing clear vision. Disruptions in fibril organization, such as corneal scarring or keratoconus, can reduce transparency.^[^ ^126^ ^]^ Furthermore, the arrangement of collagen fibrils is crucial for imparting the mechanical strength necessary for the cornea to maintain its curved shape.^[^ ^127^ ^]^ The precise alignment and spacing of these fibrils within the corneal stroma contribute to the cornea's ability to withstand intraocular pressure and external forces, thereby preserving its structural integrity and curvature. Various approaches have been employed to address collagen arrangement, fibrillogenesis, and denaturation, including electrocompaction,^[^ ^16^, ^128^ ^]^ freeze‐drying,^[^ ^129^ ^]^ electrospinning,^[^ ^17^ ^]^ extrusion, cyclic stretching,^[^ ^18^ ^]^ magnetic alignment,^[^ ^130^ ^]^ and microfluidics.^[^ ^107^, ^131^, ^132^ ^]^ The electrocompaction technique relies on the movement of particles toward anode or cathode, depending on the protein charge, driven by electrophoretic forces. For instance, Gurkan et al. demonstrated the orientation and migration of bone marrow stromal cells and tendon‐derived fibroblasts on a 3D construct composed of electrochemically aligned collagen bundles for musculoskeletal regeneration.^[^ ^133^ ^]^ As a CTE application, Kishore et al. reported that a pH gradient induced by an electric field facilitated the formation of a densely assembled collagen matrix.^[^ ^128^ ^]^ This matrix mimicked the corneal niche, thereby contributing to enhanced mechanical stability.

Electrospinning offers advantages such as a high area‐to‐volume ratio, high porosity, and ease of parameter control to create aligned collagen fibers. Human keratocytes and epithelial cells cultured on a PVA/collagen electrospun composite showed increased proliferation due to the aligned electrospun fibers^[^ ^106^ ^]^ (Figure 2F). However, electrospinning may involve toxic solvents that pose risks to cells and present challenges in the controlled collection and alignment of nanofibers.^[^ ^134^ ^]^ Hence, a more cell‐friendly method, such as cyclic stretching, can be employed to align the collagen nanofibers. For instance, Nam et al. reported that the precisely controlled continuous cyclic stretching of hydrogel sheets results in the formation of highly aligned collagen nanofibers. This alignment is achieved through meticulous regulation of the stretch frequency, magnitude, and duration, ensuring optimal structural organization^[^ ^18^ ^]^ (Figure 2E). Perpendicular elongation of fibers was observed after 12 and 24 h of stretching, with 40% porcine corneal stromal cells reorganized in the identical direction.

Shear forces induced during bioink extrusion can also be utilized to align the internal macromolecules in the direction of flow, resulting in alignment and shear‐thinning behavior of collagen fibrils. A study revealed that collagen bioprinting resulted in controlled orientation and morphology of fibroblasts.^[^ ^135^ ^]^ Nerger et al. demonstrated that collagen could be bioprinted by manipulating the geometry and alignment of the fibrous network.^[^ ^101^ ^]^ Matrigel was integrated into the bioink to extrude lower concentrations of collagen, resulting in spatially controlled fiber geometry and alignment. Another study used a stroma‐derived dECM bioink for extrusion‐based bioprinting (to be discussed in more detail in Section 5), where collagen fibril alignment was achieved along the direction of shear forces four weeks after transplantation, resulting in the formation of a lattice pattern that mimicked the structural characteristics of the native human cornea.^[^ ^136^ ^]^ It is important to recognize that shear stress plays a significant role in determining cell behavior during the printing process. The level of shear stress can affect critical cellular functions, including apoptosis and changes in the cytoskeleton.^[^ ^137^ ^]^

#### 3.1.7 Permeability

Permeability refers to the ability of CEnCs to facilitate the transport of nutrients. Given the avascular nature of the cornea, essential nutrients, particularly glucose, are transported from the aqueous humor through CEnCs to sustain corneal metabolism and function. This highlights the significance of a scaffold that demonstrates high permeability to allow the passage of nutrients and biomolecules, like glucose and albumin. The permeability of a scaffold can be evaluated by quantifying the diffusion coefficient of glucose across the structure. The diffusion coefficients for glucose and albumin through the cornea in humans are (2.6 ± 0.3) ×10^−6^ cm^2^ s^−1^ and (1.0 ± 0.2) ×10^−7^ cm^2^ s^−1^, respectively.^[^ ^15^, ^138^ ^]^

### 3.2 Bioprinting Modalities

Engineering a functional corneal construct requires careful consideration of multiple design parameters, including transparency, collagen fibril alignment, mechanical integrity, permeability, swelling, and degradation behavior, and the ability to prevent neovascularization. Each of these features plays an essential role in mimicking the structure and function of the native cornea and achieving clinical success. Reproducing these characteristics in a controlled and reproducible manner necessitates the use of advanced fabrication techniques.

To address these challenges, a variety of bioprinting modalities have been explored in CTE. The following section discusses different bioprinting modalities utilized in CTE, including light‐based, droplet‐based, extrusion‐based, and laser‐based bioprinting.

#### 3.2.1 Light‐Based Bioprinting (LBB)

LBB uses controlled exposure of light to selectively solidify bioinks at high resolution, which allows the construction of accurate, detailed 3D structures. LBB encompasses stereolithography (SLA), digital light processing (DLP), and volumetric bioprinting (VBP), which use UV or alternative light sources to facilitate the polymerization of bioinks to develop 3D constructs based on computer‐generated designs.^[^ ^139^, ^140^, ^141^ ^]^

**Figure 3:**
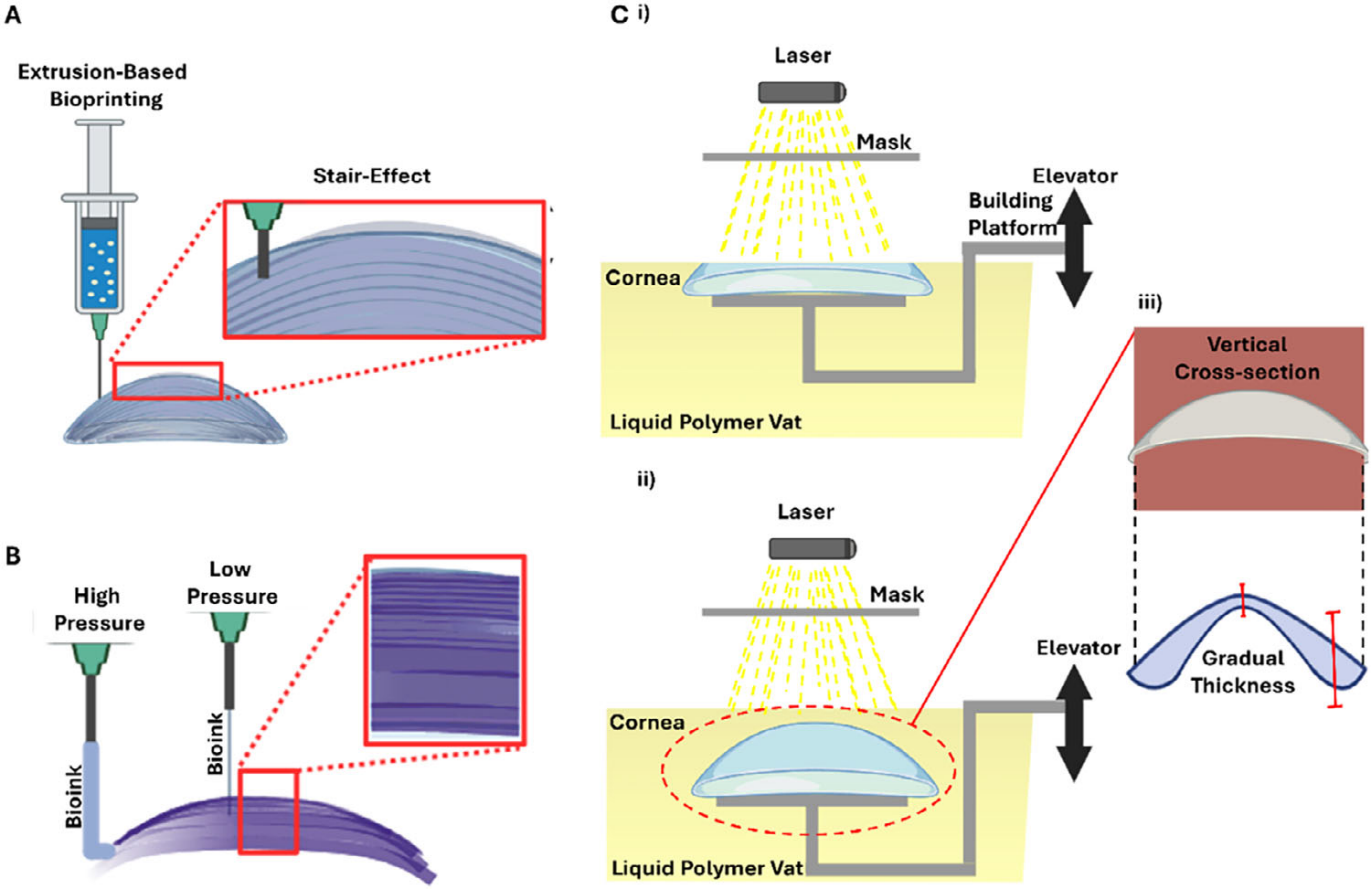
A) Corneal constructs are generated layer by layer using EBB, which can result in visible stair‐step artifacts. B) Gradual thickness variation can still be achieved by modulating extrusion pressure. C) i‐ii) SLA utilizes layer‐by‐layer photopolymerization within a liquid resin vat, allowing for smoother vertical profiles and curvature‐controlled fabrication. iii) Cross‐sectional views highlight the SLA's ability to achieve continuous gradients in thickness, mimicking the native dome geometry. *Created with BioRender.com*.

SLA utilizes light to solidify a photosensitive resin, producing complex patterns at high resolution.^[^ ^142^ ^]^ It involves spatiotemporal solidification of the polymeric substrate via light‐induced cross‐linking of its molecular chains, avoiding the requirement for cytotoxic acid/base catalysts.^[^ ^143^ ^]^ However, SLA bioprinting techniques typically require UV or near‐UV light wavelengths to provide sufficient energy for cross‐linking reactions, potentially damaging cellular DNA, thereby increasing the risk of cell death.^[^ ^144^ ^]^ One of its primary advantages is its capacity to produce fine details and smooth surfaces,^[^ ^145^ ^]^ thus enabling the fabrication of complex geometries and internal structures, which is essential for mimicking the architecture of the native cornea. Hence, SLA has the potential to address the challenge of replicating the cornea's gradual thickness variation from the center to the periphery, as well as its curved, dome‐shaped geometry through precise, voxel‐based photopolymerization. In SLA, photocurable hydrogels (PEGDA, HAMA) are patterned to ≈20 µm in‐plane resolution.^[^ ^146^, ^147^ ^]^ This allows fine control over curing depth and lateral dimensions at each location, enabling thickness to be gradually varied across a construct. To mimic the native cornea's central‐to‐peripheral thickening (≈550 µm centrally to ≈700 µm peripherally), spatial modulation of exposure dose controls the local cure depth of photocurable hydrogels, producing thicker or thinner regions as needed. Because SLA does not rely on discrete physical layers but rather on optically defined cross‐sections, it can produce continuous vertical gradients without stepwise transitions. Similarly, the dome‐shaped curvature is achieved based on the cross‐sectional slices of the 3D model, allowing SLA to generate smooth, curved surfaces that closely replicate corneal topography (**Figure** **3**). This level of resolution and geometric fidelity makes SLA particularly suited for fabricating anatomically accurate corneal constructs. Additionally, it does not subject the bioink to shear stress, thereby preserving cell viability during bioprinting.^[^ ^148^ ^]^ However, a significant challenge lies in the limited compatibility with available materials, as the selection of photosensitive biomaterials is restricted, and the development of new biocompatible resins remains complex.^[^ ^149^ ^]^ Further, post‐processing steps, such as washing and additional curing, are often required to achieve the desired mechanical properties and stability in bioprinted constructs.^[^ ^150^ ^]^ An artificial cornea must replicate the native cornea's characteristics, such as strength, elasticity, and transparency, while ensuring biocompatibility. Despite SLA's precision in corneal bioprinting, challenges related to limited material and UV‐induced cellular damage must be addressed to fully realize its potential for developing functional, biocompatible corneal tissues. Kutlehria et al. used SLA to develop molds for bioprinting corneal stromal equivalents since SLA allowed for high‐throughput manufacturing by enabling the simultaneous casting of several molds.^[^ ^21^ ^]^ In another study, a bioink was formulated using eosin Y and triethanolamine (TEA) as photoinitiators, 1‐vinyl‐2‐pyrrolidinone (NVP) as a cross‐linking agent, and a combination of GelMA and stromal cells. SLA facilitated the layer‐by‐layer fabrication of a dome‐shaped corneal stroma, enabling cell deposition mimicking the human cornea's structure.^[^ ^59^ ^]^

DLP employs a digital micromirror device to project patterned light of optimal wavelength and exposure time onto a photopolymer resin, triggering its curing.^[^ ^143^, ^151^ ^]^ DLP is valued for its high resolution and fast printing speed, making it effective for fabricating detailed 3D structures. However, it has limitations in terms of material compatibility and may struggle with feature precision at very small scales compared to other techniques. In contrast, two‐photon polymerization (2PP) is an advanced approach that utilizes a focused laser to cure photopolymer resins through the simultaneous absorption of two photons. This process enables the creation of highly complex 3D structures with sub‐micrometer resolution, surpassing DLP in precision. However, 2PP operates at a slower speed, making it less efficient for large‐scale production. Both DLP and 2PP offer significant potential in tissue engineering, particularly for CTE. DLP's speed and resolution make it suitable for customized scaffolds, while 2PP's precision enables the fabrication of highly detailed microstructures, supporting the development of functional tissue constructs.^[^ ^141^, ^152^ ^]^ A study reports a fast‐curing, cytocompatible (hyaluronic acid) HyA‐based bioink for DLP bioprinting, using novel water‐soluble photoinitiators (lithium benzoyl (phenyl) phosphinate), offering better photocuring efficiency and lower cytotoxicity than the traditional photoinitiator lithium phenyl(2,4,6‐trimethylbenzoyl) phosphinate (LAP).^[^ ^153^ ^]^ This bioink, blended with low and high‐molecular‐weight HyA macromers, produced a dome‐shaped corneal scaffold with >80% cell viability and >90% transparency in the visible range. Despite the challenges posed by traditional photoinitiators, the study highlights that using advanced photoinitiators in DLP is essential for preserving high cell viability and enabling the development of functional bioprinted tissues.

Volumetric bioprinting offers rapid fabrication of high‐resolution objects within seconds by utilizing tomographic light projection to simultaneously polymerize an entire 3D structure, rather than building it layer by layer like in traditional DLP or SLA bioprinting.^[^ ^141^, ^154^ ^]^ In DLP and SLA bioprinting, the construct is fabricated one layer at a time, with each layer requiring separate exposure to light for cross‐linking. This layer‐by‐layer approach can lead to slow fabrication times, stair‐stepping artifacts, and potential mechanical weaknesses between layers. In contrast, volumetric bioprinting projects light patterns from multiple angles into a rotating, cell‐laden hydrogel or bioresin. The intersecting light beams trigger simultaneous polymerization throughout the entire volume, forming the desired shape almost instantly. This technique eliminates layering artifacts, reduces mechanical weaknesses, and significantly accelerates bioprinting, making it advantageous for fabricating delicate, cell‐laden tissues like the cornea.^[^ ^153^ ^]^ However, volumetric bioprinting has limitations, including cytotoxic effects due to the presence of free radicals from resin.^[^ ^155^ ^]^ This approach limits printable cell densities, as the heterogeneous optical properties of cells cause light scattering, reducing printing quality above 2.5–5 × 10⁶ cells/ml^−1^.^[^ ^156^ ^]^ Moreover, achieving homogeneous cell distribution within bioprinted constructs could be challenging, as cells could settle or aggregate during bioprinting.^[^ ^157^ ^]^ Furthermore, the need for complex optical setups and precise adjustment of printing parameters makes the technique more costly.^[^ ^157^ ^]^ LBB techniques hold significant potential for application in CTE due to their ability to precisely replicate the cornea's shape and curvature. These techniques enable high‐resolution bioprinting, which is challenging to achieve with other bioprinting modalities.

#### 3.2.2 Droplet‐Based Bioprinting (DBB)

DBB is a sophisticated technique for constructing 3D structures using discrete droplets of bioink. This method encompasses various droplet formation principles such as electrohydrodynamic jetting, inkjetting, acoustics, and micro‐valve mechanism.^[^ ^158^, ^159^, ^160^ ^]^ Each of these mechanisms leverages unique physical forces to precisely control the creation of bioink droplets and their deposition, enabling the development of complex biological constructs with high spatial resolution. Through the process of inkjet bioprinting, bioink droplets with various physical characteristics, including density, surface tension, elasticity, and viscosity, are deposited onto a substrate. There are two classifications for inkjet bioprinting: continuous inkjet (CIJ) and drop‐on‐demand (DOD) inkjet bioprinting. Due to its precise biomaterial control and cost‐effectiveness, DOD inkjet bioprinting, which includes electrostatic, thermal, and piezoelectric techniques, is preferred for certain applications. However, this approach is limited by issues such as nozzle clogging and cell damage during bioprinting, especially when using high‐concentration bioinks.^[^ ^161^ ^]^ EHDJ uses high voltage to release smaller droplets and is ideal for bioprinting bioinks with high cell concentrations. It offers precision and minimal cell damage but faces challenges like electric field accumulation and process control difficulties.^[^ ^161^, ^162^, ^163^, ^164^ ^]^

In CTE, DBB could be a promising technique in terms of achieving similar transparency to the native cornea.^[^ ^86^ ^]^ Its ability to maintain cell viability and print with multiple cell types makes it well‐suited for fabricating layered corneal structures, including epithelial, stromal, and endothelial layers. Additionally, bioink droplet depositions in a spatiotemporally manner could offer a thinner layer at a precise location for epithelium and endothelium compared to EBB. It could potentially offer intraoperative corneal bioprinting of a photocross‐linkable construct, enabling real‐time tissue fabrication during surgery by allowing for high‐resolution patterning essential for corneal tissue. Its non‐contact bioprinting mechanism minimizes contamination risks, which is crucial in a surgical environment. Additionally, DBB's high‐throughput capability facilitates rapid fabrication of tissue constructs with multilayers, aligning with the time‐sensitive nature of surgical procedures. However, challenges such as limited bioink viscosity range and potential nozzle clogging need to be addressed to fully realize its intraoperative potential for corneal tissue.

#### 3.2.3 Laser‐Assisted Bioprinting (LAB)

LAB is a nozzle‐free method that uses a laser beam to precisely deposit bioink droplets. LAB uses techniques such as laser‐guided direct writing (LGDW) and laser‐induced forward transfer (LIFT). The LGDW technique utilizes optical traps to facilitate the guidance of cells onto a substrate, whereas the LIFT technique employs laser‐induced jet formation for moving bioinks toward a receiving layer. Although LAB offers high resolution and avoids nozzle clogging issues, it requires a complex and costly setup. Additionally, a coating layer is necessary to facilitate proper interaction between the laser and the bioink for effective curing and precise patterning. However, the manual application of this coating is often inefficient and labor‐intensive. Achieving and maintaining a uniform coating layer presents significant challenges, potentially resulting in variability and inconsistencies in the quality of the final structure. This inconsistency can adversely affect the structural integrity and functional performance of bioprinted constructs. These issues reduce the overall efficiency of the process.^[^ ^161^ ^]^ A study utilizing LAB for corneal engineering demonstrated its ability to print high‐viscosity, high‐cell‐density bioinks with high resolution (<10 pL droplets) while maintaining cell viability.^[^ ^165^ ^]^ Unlike nozzle‐based techniques, LAB enables precise spatial organization of multiple cell types without subjecting them to shear stress. This approach is crucial for fabricating clinically relevant corneal structures, as it supports the development of multilayered, shape‐retaining constructs, enhancing the potential for corneal regeneration and transplantation. Despite the high precision and nozzle‐free advantages of LAB, its broader application in corneal bioprinting remains limited by the need for bioinks specifically tailored to its laser‐based droplet formation mechanism, as well as the development of xeno‐free formulations compatible with laser energy and cell viability.

#### 3.2.4 Extrusion‐Based Bioprinting (EBB)

EBB is an additive manufacturing technique that involves extrusion of a bioink through a nozzle, which is then deposited along a predetermined path to develop a 3D structure. EBB allows for the layer‐by‐layer deposition of bioinks, facilitating the construction of complex tissue constructs for various applications. EBB uses bioinks with a wide range of viscosities, offering shear shear‐thinning feature.^[^ ^166^ ^]^ Highly viscous polysaccharide materials can be used with improved adhesivity, which allows cell aggregates and proteins to be suspended in the bioink's hydrogel composition.^[^ ^167^, ^168^ ^]^ The printability of a bioink depends on extrusion pressure, nozzle diameter, bioprinting speed, and the process temperature. EBB can generate scalable constructs with a wide range of bioinks to be discussed in Section 3.3, but it offers lower resolution as compared to other techniques.^[^ ^169^, ^170^, ^171^ ^]^ The bioink must possess specific properties, including transparency, biocompatibility, and mechanical strength, to closely resemble the native cornea, in addition to shear‐thinning behavior to ensure smooth extrusion and accurate layer formation during bioprinting. A range of hydrogels, including collagen, gelatin, and synthetic polymers, has been tested to formulate appropriate bioinks for CTE.^[^ ^172^, ^173^, ^174^, ^175^, ^176^ ^]^ Moreover, the inclusion of growth factors, such as TGF‐β and epithelial growth factor (EGF), has the potential to enhance the process of corneal tissue regeneration by triggering fibroblasts to accelerate the healing process and promoting epithelial cell proliferation and migration for re‐epithelialization.^[^ ^177^, ^178^ ^]^

Although EBB has demonstrated initial success in CTE, several limitations must be overcome. One significant challenge is the shear stress induced during extrusion. This shear stress can damage cells, reducing their viability and impairing their function, which ultimately affects the quality of the regenerated tissue. Ensuring optimal cell viability and preserving cell function throughout the bioprinting procedure is vital for the effective regeneration of tissues.^[^ ^179^ ^]^ Additionally, EBB has other disadvantages that can impact cell viability and function. For example, the process is prone to nozzle clogging,^[^ ^93^ ^]^ which not only disrupts the bioprinting process but can also cause additional damage to cells.^[^ ^180^ ^]^ Furthermore, EBB often requires the use of viscous bioinks to maintain the shape of bioprinted constructs. In some cases, support baths are necessary to stabilize these bioinks during bioprinting. Support baths are typically yield‐stress materials that provide physical confinement during bioprinting, enhancing resolution and shape fidelity.^[^ ^181^ ^]^ Also, EBB fails most obviously when its tool‐path logic collides with the cornea's pseudo‐orthogonal lamellae. In native stroma, every 2‐ to 3‐µm collagen lamella rotates ≈90° relative to the one below, and the fibrils themselves follow gently curved geodesics across the corneal dome.^[^ ^182^ ^]^ However, standard slicers flatten this curvature into planar, parallel rasters. Dozens of layers are bioprinted with identical filament direction simply because it is faster. Instead of using planar slicing, curved/non‐planar slicing gave better fidelity.^[^ ^183^ ^]^ Lamellar misorientation could be observed in EBB‐built constructs. Repeating one raster direction produces a bulk composite that is strong in one axis but weak and optically irregular in the orthogonal plane, contradicting the cornea's cross‐ply reinforcement. Also, converting a dome into stacked flats forces each filament to terminate at abrupt z‐jumps. The resulting step edges scatter light and create shear planes vulnerable to delamination, mentioned as a step effect. Shear‐alignable bioinks orient their internal fibrils along the flow through the nozzle. If the print‐head lays every layer in the same direction, all collagen fibrils align along a single axis. This over‐reinforces that axis while leaving the perpendicular direction weak, depriving keratocytes of the cross‐hatched guidance cues they need for proper orientation. Moreover, linear deposition paths fail to conform to the cornea's natural curvature; instead, they cut across the dome at awkward angles, creating localized stress points and optical phase shifts that reduce transparency. To more accurately replicate the native corneal architecture, path planning should transition from planar slicing to curvature‐conformal tool paths generated directly from patient optical coherence tomography data. Raster orientations should be systematically rotated between successive layers, by 90° in bilayer constructs or 60° in tri‐ply configurations, while maintaining sub 10‐µm layer heights to approximate natural lamellar spacing. To enable continuous, curvature‐following filament trajectories without discontinuities or start‐stop artifacts, hardware platforms must support either multi‐axis (5‐DOF) deposition or embedded bioprinting within optically transparent support matrices.

#### 3.2.5 Other 3D Printing Methods Used in CTE

Material extrusion is an additive manufacturing technique that thermally fuses filaments after deposition according to a computer‐assisted design (CAD). Generally, thermoplastics are used as inks to manufacture constructs, as they soften in the melting chamber of the nozzle that facilitates the continuous flow of inks through an extruder. The temperature regulated by a fan attached beside the nozzle assists in the solidification of molten inks to form a stable solid construct. Polymers with low melting points are used, like polycaprolactone (PCL). However, PCL, as a synthetic biomaterial, may require the incorporation of additional biomaterials such as natural polymers or bio‐ceramics to enhance biocompatibility and surface properties to improve cell adhesion and growth.^[^ ^184^, ^185^ ^]^ Fused deposition modeling (FDM) offers high precision and control over the shape and structure of printed scaffolds, as well as to incorporate multiple materials into inks.^[^ ^169^, ^170^, ^171^, ^186^ ^]^ Molding techniques are also utilized to fabricate negative replicas of a particular form or configuration. FDM is frequently used in the content of CTE to fabricate corneal scaffolds or molds (that support the corneal structure before a cast hydrogel cross‐links for the subsequent architecture).^[^ ^19^, ^20^, ^21^ ^]^ For instance, Velázquez et al. developed personalized cornea models to understand an asymmetric disease condition, which aim to enhance doctor‐patient communication and improve patient understanding of keratoconus.^[^ ^24^ ^]^ The utilization of suitable biomaterials and molds enables researchers to fabricate corneal constructs that exhibit a high degree of similarity to the architecture of the native cornea, thereby facilitating the process of regeneration.

#### 3.2.6 Comparison among 3D Printing Modalities for Corneal Tissue Engineering

Shape fidelity of corneal substitutes is a critical attribute responsible for their performance, and 3D printing has emerged to be a promising tool to achieve the native corneal dome‐shaped structure. Different 3D printing modalities offer their respective advantages to achieve a 3D dome‐shaped structure of required dimensions and physical properties. SLA can replicate a smooth, continuous curvature close to anatomic geometry owing to its high precision.^[^ ^59^ ^]^ The advantages of SLA lie in its ability to produce seamless, complex curves; even thin‐layered corneal architectures (stromal lamellae) can be produced with minimal stair‐stepping.^[^ ^187^ ^]^ However, standard DLP printers have limited z‐resolution, which when coupled with the inherent mechanical softness of the hydrogel, can cause subtle stepped layer artifacts on curved surfaces, reducing shape fidelity for the smooth corneal dome.^[^ ^61^ ^]^ While some modern DLP printers achieve ≈10 µm resolution with non‐biocompatible resins, and even sub‐10 µm (≈1 µm) features in specialized setups using conventional acrylates,^[^ ^188^ ^]^ DLP bioprinters with hydrogels typically produce features in the 10–20 µm range.^[^ ^189^ ^]^ One study found that a DLP‐printed curved cornea model showed visible terracing and fragility, to the point that the team opted to print flatter layers for implantation.^[^ ^61^ ^]^ Thus, while SLA‐based bioprinting offers excellent architectural control (even enabling intricate microstructures and cell placement within the dome), it is constrained by biomaterial requirements (only certain polymers like methacrylates are usable) and by the need to balance curing for shape fidelity against cell health. However, DLP enables the cornea's natural thickness profile. One study developed a dome‐shaped construct whose thickness gradually increased from the central apex to the peripheral edge.^[^ ^190^ ^]^

EBB, in contrast, builds the corneal shape by depositing continuous filaments in a layer‐by‐layer fashion. A dome shape can be achieved by printing concentric rings while changing the z‐height or 3D spirals that gradually narrow in diameter.^[^ ^122^ ^]^ The researchers extruded collagen/alginate hydrogel containing human corneal stromal cells in concentric circles to produce a cornea‐shaped construct in under 10 min. The bioink's rheology is the most critical attribute for successful EBB of a corneal construct. A bioink must be fluid enough to extrude through a nozzle, but it should solidify immediately after extrusion on a build plate to maintain the dome geometry. In a study, an alginate/collagen bioink was formulated with optimized rheological properties, which ensured sufficiently low viscosity to extrude, whilst maintaining gel stiffness to hold its shape, preserving the printed curvature without collapse.^[^ ^122^ ^]^ Advantages of EBB include its versatility with bioinks, as it can process bioinks with higher viscosities and particle/cell concentrations than inkjet bioprinting. Moreover, multi‐nozzle EBB allows the incorporation of multi‐material deposition, required for layered constructs. Notably, EBB can recreate key stromal corneal ultrastructure by spatially organizing filaments by tuning print paths and using alignment guides, and printed collagen fibers can mimic the orthogonal lamellae of the native stroma. The shape fidelity of EBB, however, is typically lower than that of SLA, as the resolution is governed by the nozzle diameter and the filament's tendency to flatten, which can introduce microscale ridges or pores. While SLA can achieve resolutions as fine as ≈10 µm with conventional resins,^[^ ^191^ ^]^ in hydrogel‐based systems, it typically produces features around ≈20 µm.^[^ ^146^ ^]^ Maintaining a smooth dome with EBB without gravity‐induced sagging is a technical challenge. Strategies like rapid in situ cross‐linking (ionic cross‐linkers for alginate or photocuring for GelMA immediately after extrusion layer by layer) and printing into support baths have been used to counter this challenge. For example, researchers have printed corneal hydrogels within a curved support frame made by DLP, effectively using a mold to help the soft bioink retain a dome shape.^[^ ^62^ ^]^ Another approach utilizes Freeform Reversible Embedding of Suspended Hydrogels (FRESH), where the bioink is extruded into a temporary gel bath that holds the layers in place. This method improved the fidelity of complex organ shapes in other soft tissues^[^ ^192^ ^]^ and could similarly support a delicate corneal dome. Limitations of EBB include its layer‐by‐layer nature, which can yield a slight stair‐step on curved surfaces and require post‐print smoothing. A current technical constraint is that smooth optical surfaces are hard to print directly by extrusion. A degree of surface polishing or hydrogel flow can smooth out striations, but the optical clarity might still be slightly less than a photopolymerized equivalent. Even so, corneal grafts fabricated via EBB have shown promising results in vivo. For example, Kim et al. bioprinted collagen‐rich corneal constructs with controlled fibril orientation and implanted them into rabbit eyes, where after 4 weeks, the engineered collagen had begun to remodel and integrated with the native tissue.^[^ ^136^ ^]^

Inkjet bioprinting follows the principle of depositing tiny droplets of bioink, ejected from a nozzle, to build up tissue in a pixelated manner. In principle, inkjet bioprinting offers high XY resolution, as it can generate droplets of the order of 50–100 µm and the ability to pattern different cell types or biomaterials using multiple print‐heads. A corneal model fabricated with drop‐on‐demand electromagnetic microvalve‐based inkjet bioprinting to print a 3D human cornea‐mimetic structure.^[^ ^86^ ^]^ Achieving a dome shape requires extremely precise control over droplet placement and gelation timing. Typically, inkjet bioprinters process low viscosity bioinks to form and eject uniform droplets without creating satellite droplets. Inkjet bioprinting suffers from low shape fidelity because each droplet spreads on impact. As you build up a thick, curved structure, droplets merge and blur together, causing loss of fine features, specifically around sharp edges.^[^ ^193^ ^]^ Inkjet bioprinting is also restricted to low‐viscosity fluids (<10 mPa·s), as it faces frequent nozzle clogging issues and generates non‐uniform droplets with high viscosity materials.^[^ ^194^ ^]^ Typically, a liquid precursor (collagen or alginate solution) is printed and subsequently cross‐linked via thermal or chemical triggers, introducing additional complexity and limiting construct thickness due to gravitational spreading prior to gelation. Furthermore, the use of low viscosity inks may lead to post‐gelation shrinkage, distorting the intended geometry. Inkjet bioprinting is thus excellent for creating thin, planar cell sheets or adding cells to a preformed construct, but a single‐step print of a thick corneal dome is difficult. Although inkjet bioprinting can rapidly cover large areas through multi‐nozzle arrays, its application in corneal tissue engineering has thus far been largely restricted to in vitro demonstrations, such as the printing of viable, phenotypically stable human corneal keratocytes in hydrogel sheets, and translation to a full‐thickness corneal implant is still in early stages.^[^ ^86^ ^]^ Nonetheless, inkjet bioprinting's precision makes it a promising adjunct technique, perhaps for bioprinting the epithelial or endothelial cell layers onto a stromal construct with high accuracy.

LAB is another cutting‐edge method relevant to cornea fabrication. This nozzle‐free technique can achieve extremely high resolution, of the order of single cells per droplet or microscale patterning, which is advantageous for recreating the organized micro‐architecture of the corneal tissue. A high‐throughput laser printing system has been reported with ≈10 µm resolution, enabling fabrication of engineered tissues with high cell density and microscale organization.^[^ ^48^ ^]^ Sorkio et al. utilized LAB to construct cornea‐mimetic tissues comprising separate bioprinted layers for epithelium and stroma, and a combined multi‐layered cornea structure.^[^ ^165^ ^]^ The principle here is that each laser pulse causes a tiny amount of bioink to propel toward the substrate, where it forms a spot and prints patterns of droplets that build up a construct. Because there is no physical nozzle, bioinks with high viscosity or those with high cell densities can be printed, making it suitable to print collagen‐rich or cell‐laden corneal stroma. Shape fidelity with LAB is promising by incorporating fine details, e.g., placing epithelial cells in a specific pattern, but bioprinting a large dome structure is laborious since the process is done dot by dot. Every voxel of the tissue must be individually deposited, which is much slower than layer‐wise methods. Consequently, building a full 3D dome by LAB might involve bioprinting many thousands of droplets in each layer and carefully stacking layers; slight misalignments can accumulate, affecting smoothness as DBB. Generally, LAB excels at micro‐scale fidelity, as it can precisely position cells or micro‐droplets, but may struggle with macro‐scale structures due to cost and process time. If the droplets do not merge perfectly, the bioprinted tissue might have microscopic discontinuities. However, researchers mitigate this issue using highly overlapping droplet patterns and bioinks that coalesce readily. It also allows bioprinting on irregular or soft substrates, which could be useful for layering cells onto a curved corneal construct. Notably, LAB can produce multi‐layered corneal constructs with different cell types in different layers in one process, which is challenging to achieve with single‐nozzle extrusion. LAB could show unique success in replicating the corneal thickness and dome shape.^[^ ^195^ ^]^ In that approach, acellular collagen layers were interwoven with cell‐containing layers, resulting in a tissue that begins to approximate native stromal organization.

Indeed, combining multiple techniques is a recurring theme in corneal bioprinting. Given that each method has inherent strengths and weaknesses, hybrid fabrication strategies are emerging to achieve the optimal corneal substitute. Researchers have tried combining DLP and EBB in one platform, where DLP was used to print a thin, concave support frame corresponding to the corneal curvature, and EBB was used to fill that frame with cell‐laden hydrogel, yielding a composite that maintains precise geometry (thickness and curvature) with high cell viability.^[^ ^62^ ^]^ This approach combined the shape precision of photoprinting with the biofunctionality of bioink.

### 3.3 Bioinks for 3D Bioprinting of Cornea

Bioprinting involves the synchronized deposition of cells and biomaterials, commonly known as bioinks.^[^ ^196^ ^]^ Bioinks facilitate the manipulation of biological and biochemical environments, enabling the creation of intricate biological structures. Bioprinting eliminates the need for cell seeding, which is a challenging additional step in traditional scaffold‐based tissue engineering.^[^ ^196^ ^]^ Additionally, it enables the precise placement of various cell types within bioprinted constructs and facilitates the reconstruction of tissues with high cell density. To attain the intended tissue constructs, it is essential to comprehend the biological and physical characteristics of bioinks and recognize the significant factors that affect tissue growth and remodeling. The selection of biomaterials and preparation and processing methods of bioinks is crucial to develop an optically, mechanically, and biochemically suitable cornea‐mimicking microenvironment. In this section, we review bioinks used in CTE.

#### 3.3.1 Collagen

Collagen has been widely utilized in the bioprinting of corneal constructs, demonstrating significant promise in the development of artificial corneas.^[^ ^14^, ^15^, ^197^, ^198^, ^199^, ^200^, ^201^ ^]^ It is a natural biocompatible material that does not trigger inflammation or cytotoxic effects, allowing for the growth and proliferation of corneal cells.^[^ ^199^, ^202^ ^]^ Collagen fibrils form the stromal structure of the cornea as the main constituent, with the native cornea containing 90% Col I in its ECM. Therefore, collagen is required in the bioink to emulate the function and structure of artificial corneas to mimic the native structure of the cornea. Its printability is mainly modulated by its physical characteristics, such as viscosity, gelation kinetics, and mechanical properties. The majority of collagen used in biofabrication of the corneal tissue is Col I, which constitutes roughly 90% of the protein mass in mammalian connective tissues.^[^ ^203^ ^]^ Collagen is obtained as collagen molecules that initiate self‐organization into fibrils at 37 °C and neutral pH.^[^ ^86^, ^173^, ^174^ ^]^ Its printability is directly affected by the kinetics of this process, with rapid cross‐linking resulting in enhanced printing accuracy. However, the main issue with collagen bioink is its poor mechanical characteristics.^[^ ^174^, ^204^, ^205^, ^206^ ^]^ In bioprinting, the thickness and structural properties of bioprinted constructs can be controlled by tuning bioprinting parameters.^[^ ^174^ ^]^ However, substantial research is essential to further evaluate the durability and refractive qualities of corneas constructed from collagen.

Collagen is widely used in corneal tissue engineering because its fibrils closely match those of the native stroma or providing a more authentic microenvironment.^[^ ^23^, ^86^, ^122^, ^165^, ^174^, ^175^, ^207^, ^208^, ^209^ ^]^ As an example, a transparent cornea was developed by bioprinting of photocross‐linkable Col I.^[^ ^26^ ^]^ The collagen bioink was prepared with 1.2% (w/v) bovine Col I solution neutralized with 0.1M acetic acid, followed by phosphate buffer. Due to precipitation issues indicated in another study, a high collagen concentration (20 mg/mL) at pH of 7.4 can lead to its precipitation, resulting in a loss of transparency.^[^ ^174^ ^]^ To address this, the use of Ca^2^⁺, Mg^2^⁺, and Sr^2^⁺ ions has been found to prevent Col I precipitation at pH of 7.4. The study also showed that Col I clarity was maintained for at least 58 days at neutralized pH. Col I was 90% and 96% transparent for 6 and 12 mg mL^−1^ concentrations, respectively, which is higher than the transparency of the native cornea in the visible light range. In another study, bioprinting of recombinant human collagen III (RhCol III) was reported by Gibney et al. with tunable optical and mechanical properties.^[^ ^175^ ^]^ Collagen constructs were bioprinted with 3.4 mg mL^−1^ RhCol III and cross‐linked with 40 µg mL^−1^ of N‐(3‐Dimethylaminopropyl)‐N′‐ethylcarbodiimide hydrochloride and 24 µg mL^−1^ N‐hydroxysuccinimide at 4 ^°^C for 18 h. As a result, the refractive index and swelling ratio were found to be 1.35 ± 0.02 and 86.7 ± 2.6%, respectively, while elastic modulus was measured as 506 ± 173 kPa. However, a potential drawback of the formation of thick fibrils during the neutralization or cross‐linking process was also noted. Despite this concern, the study confirmed that the transparency of RhCol III remained ≥87%, which was within an acceptable range for corneal substitutes.

##### Collagen Combined with Biomolecules

Optimization of a bioink formulation is necessary to obtain adequate printability during bioprinting and to prevent undesirable characteristics of bioprinted constructs, such as swelling and shrinkage.^[^ ^210^ ^]^ Although collagen emulates the natural environment of the cornea, it does not provide superior printability and has low mechanical strength at lower concentrations. Various approaches have been followed to bioprint collagen with the desired shape and mechanical stability.^[^ ^101^, ^173^, ^211^ ^]^ One of the approaches is to combine collagen with biomolecules. For instance, Sorkio et al. combined Col I and recombinant laminin‐511 (LN511) and LN521 to demonstrate that this mixture provided enhanced mechanical stability, proliferation of human limbal epithelial cells in vitro, and printability of corneal epithelium.^[^ ^165^ ^]^ LAB was used for depositing human embryonic stem cell‐derived limbal epithelial stem cells to print epithelium‐mimicking structures. Human adipose tissue‐derived stem cells (hASCs), collagen, and recombinant laminin were used as a bioink to create a layered stroma‐like structure. Cell viability analysis demonstrated that all models exhibited increased cell proliferation up to Day 7, with hASCs combined with collagen showing superior printability. hASCs bioprinted in line patterns were more uniformly distributed than point patterns. Though the tissue‐engineered cornea mimicked the native cornea, it showed degradation after 7 days and even faster in the presence of collagenase. Though it was not stable enough for long‐term studies, it has promising results that explored a path toward the construction of the cornea only using human‐derived raw materials and without further need for cross‐linking, which was otherwise required in alginate‐ and gelatin‐based biomaterials.^[^ ^165^ ^]^

##### Collagen Combined with other Biomaterials

**Figure 4:**
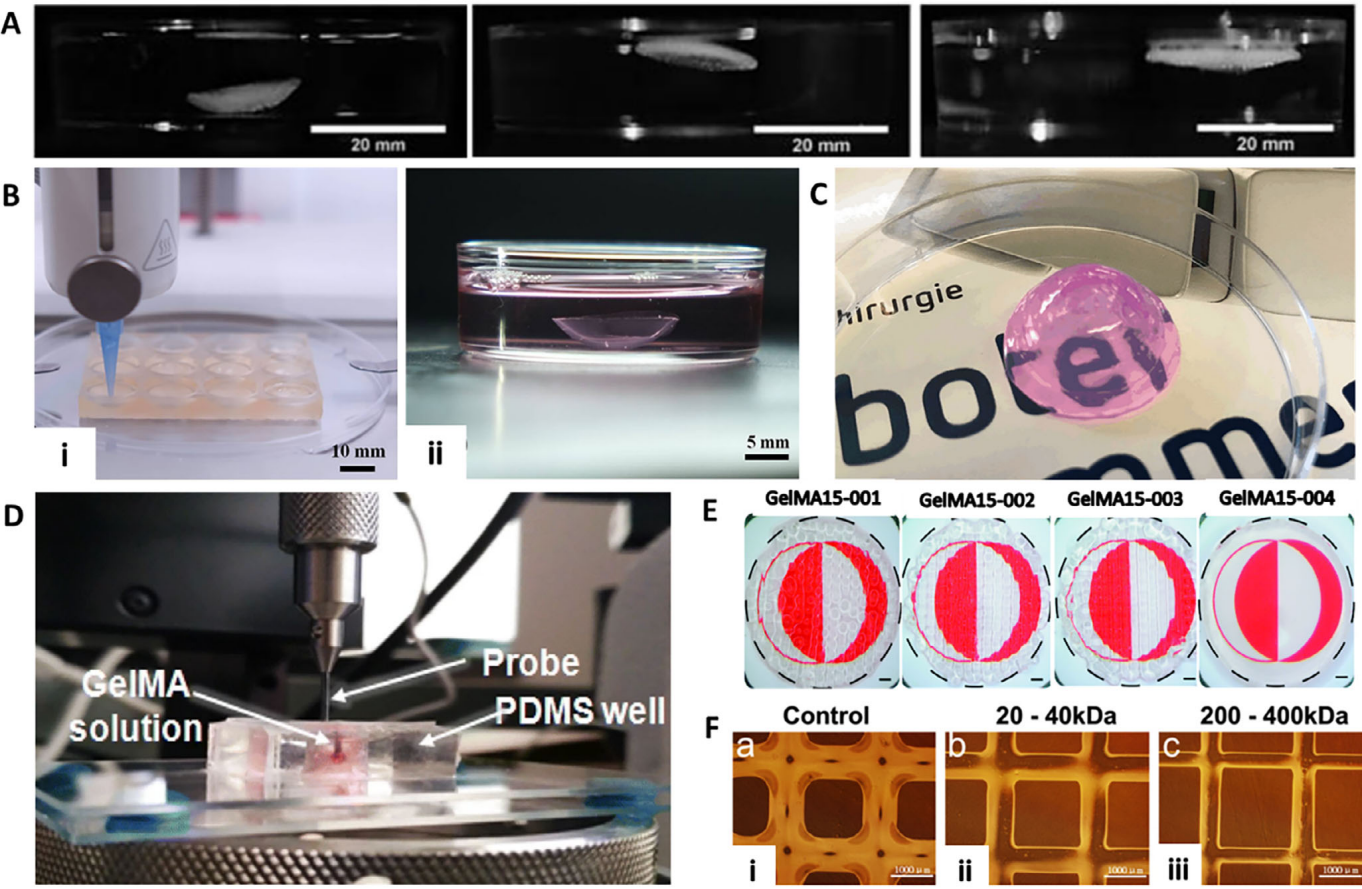
Example of collagen‐ and gelatin‐based bioprinted constructs. A)(i) Photographs of bioprinted constructs using (i) 1:2 ratio of 8 mg/ml collagen and alginate; (ii) 1:3 ratio of 8 mg ml^−1^ collagen and alginate, (iii) 1:2 ratio of 6 mg ml^−1^ collagen and alginate (adapted and reproduced with permission from ref. [122] 2018, Elsevier Ltd). B) The human corneal stroma design: (i) bioprinting process and (ii) a bioprinted corneal construct within medium after cross‐linking (adapted and reproduced with permission from ref. [21] 2020, Wiley Periodicals, Inc). C) Bioprinted dome‐shaped cornea made of 0.5% agarose with 0.2% Col I (adapted and reproduced with permission from ref. [86] 2019, Wiley Periodicals, Inc). D) Measuring the drag force with a probe in a bioprinted construct (adapted and reproduced with permission from ref. [59] 2020, Biomedical Engineering Society). E) Representation of transparency of GelMA on Day 0 (scale bar: 1 mm). GelMA15‐001 referred to 0.01 dots per minute spindle spin, 200 mm min^−1^ nozzle speed, with printed 5 layers of GelMA in vitro. GelMA15‐002 referred to 0.02 dots per min spindle spin, 200 mm min^−1^ nozzle speed, with printed three layers of GelMA in vitro. GelMA15‐003 referred to 0.03 dots per min spindle spin, 300 mm min^−1^ nozzle speed with printed three layers of GelMA in vitro (adapted and reproduced with permission from ref. [60] 2020, The Royal Society of Chemistry). F) Bioprinted GelMA constructs (i) without HyA and with (ii) 20‐40 and (iii) 200‐400 kDa HyA (adapted and reproduced with permission from ref. [215] 2022, Elsevier B.V).

Col I at low concentrations possesses poor mechanical characteristics, whereas high concentration of Col I often results in lower transparency.^[^ ^174^ ^]^ Therefore, researchers have utilized collagen with a combination of different hydrogels, such as alginate, gelatin, to increase mechanical stability and maintain transparency instead of using high‐concentration Col I.^[^ ^15^, ^86^, ^212^, ^213^, ^214^ ^]^ For example, Isaacson et al. developed a functional artificial stroma from a 3D digital human corneal model using a bioink comprised of methacrylated collagen and alginate (**Figure** **4**A).^[^ ^122^ ^]^ A bioprinter with a dual extrusion head was used to print the bioink before cross‐linking with calcium chloride (CaCl_2_). The keratocyte viability of 83% was observed up to 7 days post bioprinting. The study revealed the feasibility of using the FRESH technique to bioprint low viscosity bioinks in a support bath, thereby producing structures that were both physiologically precise and supported the proliferation of corneal keratocytes. Furthermore, Kutlehria et al. addressed the demand for in vitro models through the development of a high‐throughput bioprinting method for corneal equivalents along with a support scaffold to preserve the natural curvature^[^ ^21^ ^]^ (Figure 4B). The optimized bioink formulation comprised a solution of sodium alginate, gelatin Type B, and Col I (Fibricol) at a concentration of 10 mg/mL. Human corneal keratocytes (HCKs) were successfully integrated into bioprinted corneas and the cell viability exceeded 95% for two weeks. As another example, Xu et al. utilized GelMA and Col I composition to increase the tensile strength of the artificial cornea.^[^ ^23^ ^]^ They used 0.5% Irgacure 2959 as a photoinitiator with 10% GelMA and 0.6% Col I. A convex biomimetic cornea was prepared for regeneration purposes to observe the behavior of the recipient's cells with the transplanted cornea. Steeper slopes of artificial cornea showed tighter adhesion than their flat counterparts, thus enhancing corneal regeneration. Traditional EBB often fails in the case of maintaining proper thickness at the middle and periphery when a bioink is extruded at room temperature due to the stair‐stepping effect. However, suppressing this effect by increasing the temperature to 27.5 or 30 °C enabled better printability. The constructs showed light transmittance of 86% at a wavelength of 750 λ (maximum wavelength where the material allows most of the light to pass through), along with tensile strength and compressive modulus of 51 and 130 kPa, respectively. As curved and flat surfaces were studied for their ability to heal injured ocular surfaces, curved surfaces showed the required plasticity to wrap up the epithelial lining for better wound covering.^[^ ^23^ ^]^ In another example, Campos et al. reported a bioprinted corneal stromal model with a bioink of agarose‐collagen loaded with corneal stromal keratocytes (CSKs) (Figure 4C).^[^ ^86^ ^]^ The bioink consisted of 0.5% agarose and 0.2% collagen with CSK density of 10^6^ cells per mL. The predesigned model was bioprinted using EBB. The viscosity of the bioink was higher than pure collagen, which allowed tunable properties like faster gelation. After 7 days of in vitro culture, CSKs regained their characteristic dendritic shape with a positive expression of keratocan and lumican, like human corneal tissue.^[^ ^86^ ^]^ In another study, Lemonche et al. compared the behavior of various stem cell subtypes in corneal scaffolds, in which corneal stromal stem cells outperformed other types.^[^ ^105^ ^]^ The bioink was composed of cellulose nanofibrils, collagen, alginate, and stem cells, including MSCs, bone marrow‐derived derived‐MSCs, and adipose derived‐stem cells (ADSCs), with the control group not having collagen. PEDF possesses anti‐angiogenic properties, and it is known that lower PEDF secretion can result in excess corneal vascularization. The constructs without collagen secreted more PEDF into to medium compared to the collagen groups. Collagen‐involved bioprinted cornea showed approximately the same cellular viability as compared to the other group. Among stem cells, stromal MSCs were superior in cell viability and PEDF secretion.

#### 3.3.2 Gelatin

Gelatin, a proteinaceous material derived from collagen, is widely used in bioinks for 3D bioprinting due to its biocompatibility and favorable cell‐interactive properties. However, gelatin‐based bioinks often lack sufficient mechanical stability under physiological conditions, leading to rapid degradation and structural failure of bioprinted constructs.^[^ ^168^, ^216^, ^217^, ^218^ ^]^ Various cross‐linking techniques can be utilized to improve the stability of gelatin‐based bioinks, encompassing chemical and physical cross‐linking. Chemical cross‐linking involves the utilization of cross‐linking agents to interact with functional groups found in gelatin molecules, namely amino or carboxyl groups, resulting in the formation of covalent bonds.^[^ ^219^ ^]^ Frequently used cross‐linking agents for gelatin bioinks include methacrylate, glutaraldehyde, genipin, carbodiimides, and transglutaminase.^[^ ^168^, ^217^, ^218^ ^]^ Physical cross‐linking techniques utilize non‐covalent interactions, such as electrostatic interactions, hydrogen bonding, and physical entanglements, to establish a gel network.^[^ ^220^, ^221^ ^]^ An alternate method to induce gelation is by subjecting the gelatin bioink to a reduced temperature. This cooling process facilitates the self‐assembly of gelatin molecules, forming gel structures via physical interactions. Additional physical cross‐linking techniques encompass UV or visible light‐induced cross‐linking, wherein photoactive moieties or photoinitiators are integrated into the gelatin bioink and are stimulated by exposure to light. For example, GelMA precursors experience radical polymerization with photoinitiators.^[^ ^25^, ^222^ ^]^ The selection of the cross‐linking technique depends on the bioprinting application and the intended characteristics of bioprinted constructs. Chemical cross‐linking frequently offers enhanced mechanical strength and stability to the bioprinted constructs; however, it can potentially raise concerns regarding cytotoxicity as a result of residual cross‐linking agents. In contrast, physical cross‐linking techniques are typically characterized by their less harsh nature and enhanced biocompatibility, albeit potentially resulting in reduced mechanical properties when compared to chemical cross‐linking.^[^ ^223^ ^]^

Arginyl, glycyl, and aspartic acid (RGD) tripeptide density determines the efficacy of cell adhesion.^[^ ^224^ ^]^ Gelatin contains RGD motifs that facilitate cell attachment by interacting with integrin receptors on cell surfaces. When gelatin is cross‐linked using the dehydrothermal (DHT) process, a method involving heat and reduced pressure to strengthen the material without adding chemicals, it maintains the natural expression levels of proteins such as ATPase, N‐cadherin, ZO‐1, and paxillin. These proteins are crucial for forming and maintaining tight junctions and adherens junctions, which are essential for cellular integrity and function in ocular tissues. Therefore, DHT cross‐linking preserves gelatin's biocompatibility and its ability to support cell adhesion and junction formation in eye‐related applications.^[^ ^168^, ^218^, ^225^, ^226^, ^227^ ^]^ Gelatin cross‐linked with ethyl dimethyl aminopropyl carbodiimide (EDC) and N‐hydroxysuccimide (NHS) demonstrates improved elasticity and porosity, as well as enhances the growth of CEnCs without affecting the morphology and functionality of the cultured cell line^.[^ ^5^, ^29^, ^228^ ^]^ Goodarzi et al. fabricated NHS‐linked Col I and gelatin hydrogel and reported that the use of Col I improved optical properties as well as mechanical strength.^[^ ^212^ ^]^

GelMA can rapidly cross‐link by utilizing UV irradiation, a property that has proven advantageous for its extensive application in CTE.^[^ ^25^, ^60^, ^190^, ^215^ ^]^ As an example, SLA bioprinted GelMA with human corneal stromal cells was successfully performed, and cross‐linking kinetics were analyzed, suggesting that cross‐linking time was enough for 5 min since most of the cross‐linking happens in up to 10 min^[^ ^59^ ^]^ (Figure 4D). The bioink was developed by 12.5% GelMA with the addition of 0.02 mM eosin Y and 74 nM 1‐vinyl‐2‐pyrrolidinone, and 0.2% triethanolamine solution for photoactivation. The results demonstrated that 12.5% GelMA could mimic the native stromal tissue in terms of mechanical stiffness and transparency. Additionally, Col I, lumican, and keratan were found to be upregulated during a 14‐day culture that mimics the native corneal physiology. Similarly, Bektas et al. developed a cornea stromal equivalent by formulating a bioink with GelMA, (HCKs and photoinitiator Irgacure 2959 [2‐(hydroxyl)‐4‐(2‐hydroxyethoxy)‐2‐methyl‐propiophenone]^[^ ^60^ ^]^ (Figure 4E). Bioprinted constructs exhibited a structural similarity with the stroma's native composition, featuring parallel structures within each layer and adjacent layers arranged perpendicularly to the previous layer. As a comparison with the study conducted by Mahdavi et al.,^[^ ^59^ ^]^, a higher concentration of GelMA was utilized by Bektas et al.^[^ ^60^ ^]^ The results confirmed that higher cell viability can be achieved in stiffer GelMA since bioprinted constructs consisting of 15% GelMA provided 98% cell viability.

He et al. developed a bioink aiming to address the brittleness typically associated with GelMA using the toughening effect of cross‐linking the PEGDA chain. The bioink consisted of rabbit MSCs along with 5% GelMA and varying concentrations of PEGDA (10, 15, and 20%).^[^ ^61^ ^]^ DLP was used with the addition of a photo‐activated bioink to initiate photopolymerization, and LAP was used as a photoinitiator. PEGDA concentration at 20% showed good printability and tensile strength compared to 10 and 15% PEGDA. The results showed that cell differentiation and spreading were enhanced after 7 days, and the absence of alpha‐smooth muscle actin (α‐SMA) indicated scarless healing. Though this work successfully constructed a corneal model, the long‐term effect on human eyes needs to be explored. In terms of printability, another study showed that GelMA/PEGDA demonstrated good printability using a biopen system (extrusion‐based). However, at higher PEGDA concentrations, above 25%, cell viability decreased, highlighting a trade‐off between structural integrity and cytocompatibility.^[^ ^229^ ^]^ Moreover, Kutlehria et al. have specially designed high‐throughput (6–12 corneas at once) bioprinting of corneal analogues.^[^ ^21^ ^]^ The optimized bioink contained 3.25% (w/v) sodium alginate, calcium chloride, 4% (w/v) gelatin combined with collagen (5 mg mL^−1^) and HCKs to increase transparency and stiffness to hold the printed curvature, which was performed at 24 °C and 14–18 kPa. The bioprinted constructs had enough stiffness to maintain their shape and curvature throughout the bioprinting process without any nozzle clogging. Cell viability was maintained until 14 days, and the growth rate was observed to be highest on Day 5. In another work, Wang et al. assessed the printability of a GelMA and HyA‐based bioink.^[^ ^121^ ^]^ A specific molecular weight (800–1000 kDa) of GelMA‐HyA bioink demonstrated better printability by showing clear edges and no breakage in constructs compared to GelMA without HyA (Figure 4F). The authors designed a path plan, which provided parallel organization of filaments enabling structural integrity. The GelMA‐HyA surface exhibited better cell growth and the expression of corneal stromal cell markers, compared to the GelMA‐only surface.^[^ ^215^ ^]^ Overall, GelMA has appealing properties for CTE since it has bioactivity, however, one of the drawbacks of GelMA is its low viscosity, which leads to poor resolution during bioprinting,^[^ ^196^ ^]^ which can be further improved when combined with HyA.^[^ ^98^ ^]^ On the other hand, higher GelMA concentrations increase the stiffness of the gel after cross‐linking, which can harm cell viability.^[^ ^230^, ^231^ ^]^ Additionally, the sterilization method used for gelatin in cell studies significantly affected the properties of gelatin‐based hydrogels.^[^ ^232^ ^]^ To address these issues and enhance the bioprinting process, further research on GelMA is essential.

#### 3.3.3 Alginate

Alginate is a frequently utilized biomaterial in tissue engineering.^[^ ^233^ ^]^ Although widely used, its application as a bioink for CTE remains an ongoing subject of research. Alginate has the capability of cross‐linking through the utilization of divalent cations, such as calcium. This cross‐linking process provides mechanical strength, enabling the bioink to retain its desired shape. It exhibits a notable water content and favorable permeability to nutrients and oxygen, both of which are crucial for the sustenance and proliferation of cells. However, achieving effective cross‐linking of alginate can be difficult and usually necessitates the use of support baths. Alginate‐based hydrogels have demonstrated compatibility with corneal cell types, supporting high cell viability, typically exceeding 80–95%, normal expression of epithelial and keratocyte‐specific markers when used in bioprinted epithelial and stromal constructs. However, native alginate is often limited by its intrinsic stiffness and lack of bioactivity, which can hinder cell adhesion and proliferation. To address these limitations, chemical modifications such as partial oxidation, incorporation of RGD peptides, or blending with ECM proteins have been employed to enhance cellular interactions and promote tissue integration.^[^ ^234^, ^235^ ^]^ In animal models, alginates show no toxicity or severe immune response. For instance, alginate‐based wound dressings have been shown to benefit from the inclusion of growth factors or therapeutic cells to accelerate epithelial regeneration.^[^ ^236^, ^237^ ^]^ In the context of bioprinting, alginate‐based composite bioinks have demonstrated favorable printability and cross‐linking behavior, enabling the fabrication of corneal constructs with clinically relevant curvature and acceptable transparency. Examples include sodium‐alginate/gelatin/collagen inks laden with human keratocytes^[^ ^21^ ^]^ and alginate‐methacrylated‐collagen blends,^[^ ^122^ ^]^ both stabilized by Ca^2^‐induced gelation. Acellular domes printed on a low‐cost platform achieved ≈7 mm central radius and clear appearance^[^ ^238^ ^]^ while hybrid extrusion‐DLP protocols reproduced patient topography with >85% light transmittance.^[^ ^62^ ^]^ Yet these successes mask persistent weaknesses. Ionically cross‐linked alginate is mechanically brittle, and its susceptibility to ion‐exchange‐driven swelling can compromise long‐term optical clarity. Additionally, the polymer's bioinert backbone limits keratocyte‐matrix signaling, issues only partly mitigated by adding collagen or methacrylate groups. Moreover, Ca^2^ diffusion gradients create non‐uniform stiffness that may distort curvature post‐implantation. Thus, further work is needed on degradability, cell‐interactive chemistries, and gradient‐free cross‐linking to translate initial print fidelity and transparency into long‐term, functional corneal tissue.

Therefore, in CTE, alginate as a bioink is typically enriched with additional components to replicate the structural and functional characteristics of the native cornea.^[^ ^21^, ^122^ ^]^ This strategy facilitates cellular attachment, proliferation, and the regeneration of corneal tissue. Furthermore, the inclusion of various growth factors or ECM, can be used to improve and augment cellular activity and facilitate the development of tissues.

#### 3.3.4 Decellularized ECM (dECM)

dECM is acquired through the elimination of cellular constituents from native tissues, resulting in the preservation of the intricate protein network and other components of ECM. The acellular matrix functions as an optimal substrate for the reconstruction of the cornea.^[^ ^239^, ^240^ ^]^ dECM serves as a bioink and presents numerous benefits for CTE. Primarily, it offers an environment that closely emulates the composition of the native tissue. The biocompatibility of dECM enhances the process of cellular adhesion, proliferation, and differentiation, thereby facilitating the development of engineered corneal tissues. Furthermore, dECM preserves the bioactive signals inherent to the native tissue. The existence of growth factors, cytokines, and signaling molecules within dECM helps in modulating cellular activities and directing the process of tissue regeneration.^[^ ^241^ ^]^ The bioactivity described herein contributes to the advancement of functional tissue constructs. Another valuable attribute of dECM is the provision of structural support. It provides a structural frame for cells to effectively organize, adhere, and align themselves, ultimately developing robust corneal constructs. Furthermore, apart from its natural characteristics, dECM has the potential to demonstrate immunomodulatory effects.^[^ ^242^ ^]^ There is substantial evidence indicating that dECM can function as a bio‐instructive frame that facilitates tissue repair and regeneration in vivo, while also governing and modulating cellular behavior, such as proliferation and differentiation.^[^ ^241^, ^243^ ^]^ The incorporation of this characteristic mitigates the potential for immune rejection after the transplantation of the engineered corneal tissue, consequently facilitating the assimilation of the construct. For instance, Zhang et al. reported a corneal dECM and GelMA‐based hydrogel for corneal stroma bioprinting.^[^ ^25^ ^]^ A complete bioink was formulated with the composition of 10% GelMA, 0.5/1% dECM, 0.5% LAP as photoinitiator, and 0.02% tartrazine as a photo absorber. The bioink was mixed with human corneal fibroblasts at a density of 10^5^ cells/mL and was bioprinted using DLP. The compressive strength of the bioink was ≈74 kPa with elasticity up to 20% strain. The transparency of the bioink was ≈89 and ≈75%, with and without cells, respectively. In another study, Kim et al. developed a versatile ruthenium (Ru)/sodium persulfate (SPS) photoinitiator system for dECM‐based bioinks that enables rapid, visible light‐activated cross‐linking, improving the structural integrity of bioprinted tissues.^[^ ^244^ ^]^ Compatible with both DLP and EBB, this system broadens biofabrication possibilities. When applied to corneal and heart‐derived dECM bioinks, it showed high cytocompatibility and efficiently formed dityrosine bonds, confirmed by reduced tyrosine residues and increased cross‐linking.^[^ ^244^ ^]^ However, the choice of dECM source, whether from different tissues or species, can affect the biological and mechanical properties of the resulting constructs. Constructs made of decellularized cornea (allogeneic or xenogeneic) most closely replicate native stromal architecture and transparency. For example, human cadaver corneas (unsuitable for transplant) can be decellularized efficiently with detergents or salts while preserving collagen lamellae, basement membranes, and optical properties.^[^ ^245^ ^]^ One study found that sodium chloride/nuclease treatment removed all cells but preserved the epithelial basement membrane intact, enabling both epithelial and stromal fibroblast growth in vitro. Likewise, a fast protocol using sodium deoxycholate preserved matrix proteins, GAGs, and transparency.^[^ ^245^ ^]^ In contrast, harsher treatments (i.e., Sodium Dodecyl Sulfate (SDS)) can disrupt collagen. Decellularized porcine corneas treated with 1.5 M NaCl retained normal fibril organization and high light transmission, whereas SDS‐treated corneas showed fibril disarray and poor optics.^[^ ^246^ ^]^ Decellularized porcine and bovine corneas likewise maintain native clarity and stiffness after optimized processing.^[^ ^247^, ^248^ ^]^ In short, cornea‐derived dECM constructs preserve the highly aligned, lamellar collagen network of the stroma, yielding near‐native transparency and mechanics if cells are thoroughly removed.^[^ ^245^, ^246^ ^]^

By contrast, dECM from non‐corneal tissues differs in composition and structure. Human skin (dermis) is rich in type I collagen and is normally opaque. However, a transparent acellular dermal matrix (TADM) can be made by selecting the deepest lamellae of human dermis, yielding a clear film. TADM exhibits light transmission essentially equal to native cornea (after dehydration) with much higher stiffness.^[^ ^249^ ^]^ Although its collagen fibers are thicker and more widely spaced than those in native corneal lamellae, it has demonstrated successful integration in rabbit lamellar keratoplasty models, supporting epithelial coverage, keratocyte repopulation within 3–6 months, and sustained transparency without inducing vascularization.^[^ ^249^ ^]^

Similarly, commercial porcine small‐intestinal submucosa (SIS), a multi‐layer collagenous patch, has been used off‐label to patch large corneal ulcers. In veterinary cases (i.e., dogs, cats), multilayer SIS grafts re‐epithelialized in about 2–4 weeks and restored corneal integrity in ≈85% of the cases.^[^ ^250^ ^]^ These SIS grafts are less transparent than the cornea and often leave some haze, but they provide a bioactive construct that supports host cell ingrowth and stromal repair. Amniotic membrane, a fetal tissue rather than dermis, is also used clinically for ocular surface repair, but it lacks stromal strength and typically requires sutures that compromise optical quality.

A decellularized salmon skin‐based hydrogel microstructure mimics corneal lamellae. These membranes are highly transparent, and after carbodiimide cross‐linking, the material becomes mechanically stronger and more durable. Importantly, human corneal epithelial cells seeded on salmon ECM show strong adhesion, proliferation, and maintenance of epithelial phenotype.^[^ ^251^ ^]^ The decellularized hydrogel thus provides a biocompatible, optically clear substrate with layered collagen similar to the cornea.^[^ ^251^ ^]^

The swim bladder (gas bladder) of fish is a naturally transparent, elastic membrane. After gentle decellularization, the bladder yielded ≈93% light transmittance, slightly higher than human cornea, and very high tensile strength.^[^ ^252^ ^]^ Rabbit corneal and stromal cells adhered to the bladder matrix, and subcutaneous implantation in rats showed lower inflammation and slower degradation than human amniotic controls.^[^ ^252^ ^]^ Thus, the fish swim bladder ECM has cornea‐like clarity and biomechanics, and it supports corneal cell growth in vitro.

A decellularized squid mantle (the musculo‐elastic mantle of cephalopods) offers a transparent scaffold with aligned collagenous fibers. This decellularized squid mantle scaffold (DSMS) retains essential extracellular proteins and fiber alignment. Its extract is non‐toxic to human corneal epithelial cells. In a rabbit corneal implantation model, DSMS integrated well and promoted stromal regeneration without any signs of rejection. Even in rat muscle, DSMS was fully degraded without an adverse response. Squid ECM can provide an amino‐acid‐rich, fiber‐aligned matrix that supports host corneal repair.^[^ ^253^ ^]^

Across studies, in vitro tests show that most dECM constructs support corneal cell viability and phenotype. Decellularized human and porcine corneas allow keratocytes and epithelial cells to attach and proliferate, provided basement membranes are preserved.^[^ ^245^, ^254^ ^]^ Likewise, cultured human limbal stem cells, stromal fibroblasts, and even melanocytes readily repopulate decellularized human corneal constructs in vitro.^[^ ^245^ ^]^ Marine‐ECM hydrogels and films permit strong adhesion of corneal epithelial cells.^[^ ^251^ ^]^

In vivo, animal studies show largely positive outcomes but with source‐dependent differences. Decellularized corneal grafts (especially cross‐linked porcine cornea) in rabbit keratoplasty retained native clarity and gained strength. One study reported cross‐linked porcine dECM maintained transparency similar to the native cornea and significantly increased tensile strength, reducing complications (perforation) in a fungal‐keratitis model.^[^ ^248^ ^]^ Human corneal dECM transplanted ex vivo onto human donor beds became fully epithelialized and repopulated by host cells.^[^ ^245^ ^]^ Transparent acellular dermal matrix grafts in rabbits also epithelialized within a month, became stromally repopulated, and remained clear without neovascularization.^[^ ^249^ ^]^ Most studies agree that decellularization reduces antigenicity. Importantly, all tested dECMs supported corneal cell repopulation in vitro and integration in vivo, though long‐term outcomes (graft survival, function) remain to be fully evaluated. Importantly, another study demonstrated that the sterilization method significantly affected biochemical, mechanical, and immunological properties of the corneal dECM. Electron beam irradiation at 5 kGy and ethylene oxide gas effectively sterilized while preserving structural integrity, printability, and cell viability, unlike autoclaving, which severely degrades collagen, GAGs, and mechanical properties. Notably, electron beam sterilization also enhanced immunomodulatory effects, promoting M2 macrophage polarization and reducing pro‐inflammatory responses.^[^ ^255^ ^]^

Overall, porcine corneal dECM is abundant and structurally similar to human tissue; however, achieving optimal transparency and immune tolerance requires gentle, SDS‐free decellularization methods and effective post‐cross‐linking. Inadequate processing can lead to disorganized collagen fibrils and an increased risk of xenogenic immune responses. Human acellular dermal matrix is plentiful and stays clear after grafting, yet its much higher stiffness risks long‐term biomechanical mismatch. Human corneal dECM remains the benchmark, preserving native lamellae and optics, but donor scarcity and batch‐specific degradation limit scalability.

dECM‐based biomaterials suffer from a lack of standardized preparation protocols, resulting in significant batch‐to‐batch variability. This inconsistency highlights the need for clear protocols to enhance reproducibility and reliability. Given the lack of consensus on a universally accepted decellularization method for corneal tissues, it is essential to establish a standardized, reproducible protocol that ensures complete cellular removal while preserving the native ECM architecture and biofunction. To ensure reproducibility and compliance with decellularization standards, all process parameters, including reagent type, concentration, exposure duration, and temperature, must be rigorously standardized and validated. Each batch should be quantitatively assessed using established biochemical assays, such as DNA content, collagen preservation, and GAG retention, to confirm that it meets predefined decellularization criteria. In the literature, several approaches have been reported for dECM preparation. For example, Shafiq et al. showed that a hypertonic NaCl wash plus nuclease treatment fully removes corneal cells while preserving the epithelial basement membrane and stromal matrix, thereby supporting both fibroblast and epithelial growth.^[^ ^254^ ^]^ In contrast, Kim et al. demonstrated a well‐established SDS/triton decellularization chemistry from cardiac tissue to create a photopolymerizable corneal dECM bioink, harnessing native matrix cues to boost cell bioactivity. However, treatment with SDS alone, while effective at cell removal, has been associated with significant disruption of the basement membrane and degradation of ECM integrity.^[^ ^244^ ^]^ Puistola et al. successfully applied a gentle decellularization protocol (1% sodium deoxycholate + DNase + DNase) to hASC‐derived corneal ECM, achieving near‐complete DNA removal and preserving key proteins. The resulting dECM supported high stromal cell viability and native‐like morphology, indicating strong bioactivity.^[^ ^256^ ^]^ Similarly, physical methods like repeated freeze‐thaw or high‐hydrostatic pressure (HHP) have achieved complete decellularization with preserved collagen lamellae. A study found HHP‐treated corneas retained normal lamellar structure despite full cell removal,^[^ ^257^ ^]^ and another reported >99% DNA removal with collagen/GAG content intact using freeze/thaw (yielding DNA <0.1 µg mg^−1^).^[^ ^258^ ^]^ In practice, a reproducible corneal dECM protocol might combine a gentle osmotic soak, a brief low‐concentration detergent step (0.1–0.5% SDS or Triton with protease inhibitors), followed by rigorous DNase/RNase digestion and extensive washes.^[^ ^254^, ^257^ ^]^ By locking in specific decellularization steps and performing consistent quality checks–such as ensuring residual DNA content remains below 50 ng mg^−1^ and, intact collagen alignment batch to batch variability can be minimized and more reliable corneal dECM constructs can be produced.^[^ ^254^, ^257^ ^]^

Current dECM‐based bioinks could face challenges in mechanical stability and printability, limiting their use in bioprinted constructs. Physical strategies like support baths and sacrificial hydrogels have improved shape fidelity but require complex post‐processing that can damage the construct. Chemical strategies, such as cross‐linkers and blending with rapid cross‐linking systems, offer alternatives but may reduce the bioactivity of the native dECM.^[^ ^259^, ^260^, ^261^ ^]^ To utilize dECM for CTE, it needs to be processed into a hydrogel or bioink with rheological properties optimized for 3D bioprinting, ensuring both printability and bioactivity are preserved.

### 3.4 Sutureless Transplantation for Large‐Scale Corneal Repair Using Bioprinted Constructs

Recent advances in 3D bioprinting have enabled the development of transparent and cell‐compatible corneal constructs designed for sutureless transplantation, a promising direction for large‐scale corneal repair.^[^ ^262^, ^263^ ^]^ These strategies commonly rely on bioadhesive chemistries integrated within printable hydrogels to promote strong tissue adhesion without the need for sutures. For instance, Bhutani et al. fabricated biopolymeric lenticules (>80% transparency) that adhered to the corneal surface via light‐triggered bonding and exhibited adhesive strength (58.67 kPa).^[^ ^264^ ^]^ However, limited ECM mimicry could restrict full biointegration. Therefore, although the approach offers optical and adhesive performance, the mechanical long‐term stability in vivo and degradation control under dynamic ocular conditions remain underexplored. Ghosh et al. bioprinted dopamine‐conjugated silk fibroin onto a decellularized corneal matrix, creating a bilayer patch with high tissue adhesion (≈85 kPa), transparency (>80%), and keratocyte compatibility.^[^ ^262^ ^]^ These results highlight the potential of bioadhesive bioprinting strategies for lamellar corneal graft applications. However, dopamine‐based chemistry concerns about oxidation stability and long‐term implant clarity. Their follow‐up studies using silk‐decelluarized corneal matrix hydrogels showed that structural reinforcement via dityrosine bonding improved print fidelity and strength while maintaining transparency.^[^ ^265^ ^]^ Yet, silk fibroin and thermal gelled decellularized corneal matrix lack hierarchical collagen architecture, which may affect long‐term stromal remodeling and optical anisotropy.

Hand‐held bioprinters can directly deposit bioadhesive hydrogels onto corneal wounds, enabling rapid, suture‐free repair. For example, You et al. reported a hand‐held biopen that delivered a transparent human platelet lysate (hPL)‐based bioadhesive into corneal perforations.^[^ ^266^ ^]^ In a rabbit model, the in situ printed hPL hydrogel successfully sealed 2‐mm corneal perforations and outperformed traditional cyanoacrylate glue. The treated rabbits showed faster wound closure, less pain, and no secondary ulcers. Another strategy is to bioprint adhesive hydrogel patches ex vivo that can be implanted onto the cornea for tissue repair. For example, Ghosh et al. developed a bioprinted transparent corneal patch using a photocurable hydrogel composed of dopamine‐conjugated methacrylated silk fibroin (a mussel‐mimetic adhesive protein) combined with decellularized corneal matrix.^[^ ^262^ ^]^ The dopamine in the silk fibroin provided strong tissue adhesion, enabling the printed hydrogel patch to stick securely to the corneal surface without sutures. Farasatkia & Kharaziha developed a two‐layered corneal film with the top layer made of micropatterned silk‐GelMA that directed stromal cell alignment and an alginate/ascorbic acid bottom for adhesion and therapeutic delivery.^[^ ^267^ ^]^ While it improved cell attachment in vitro, its adhesive durability and optical clarity in vivo remain untested. Zhao et al. introduced an ion‐activated bioadhesive hydrogel, a decellularized corneal ECM‐alginate hydrogel cross‐linked by calcium.^[^ ^268^ ^]^ It sealed 6‐mm rabbit corneal defects and restored transparency with nerve and stromal regeneration. However, the method depended on ion release from the contact lens, which may limit control and reproducibility. Shen et al. developed a decellularized cornea‐HAMA dual‐cross‐linked hydrogel that bonded covalently to host tissue.^[^ ^269^ ^]^ It produced transparent, stable grafts with reduced fibrosis in rabbits. Yet, photocuring and chemical coupling steps could pose challenges for rapid, in situ clinical use.

### 3.5 In Vivo Assessment of 3D Bioprinted Corneas

**Figure 5:**
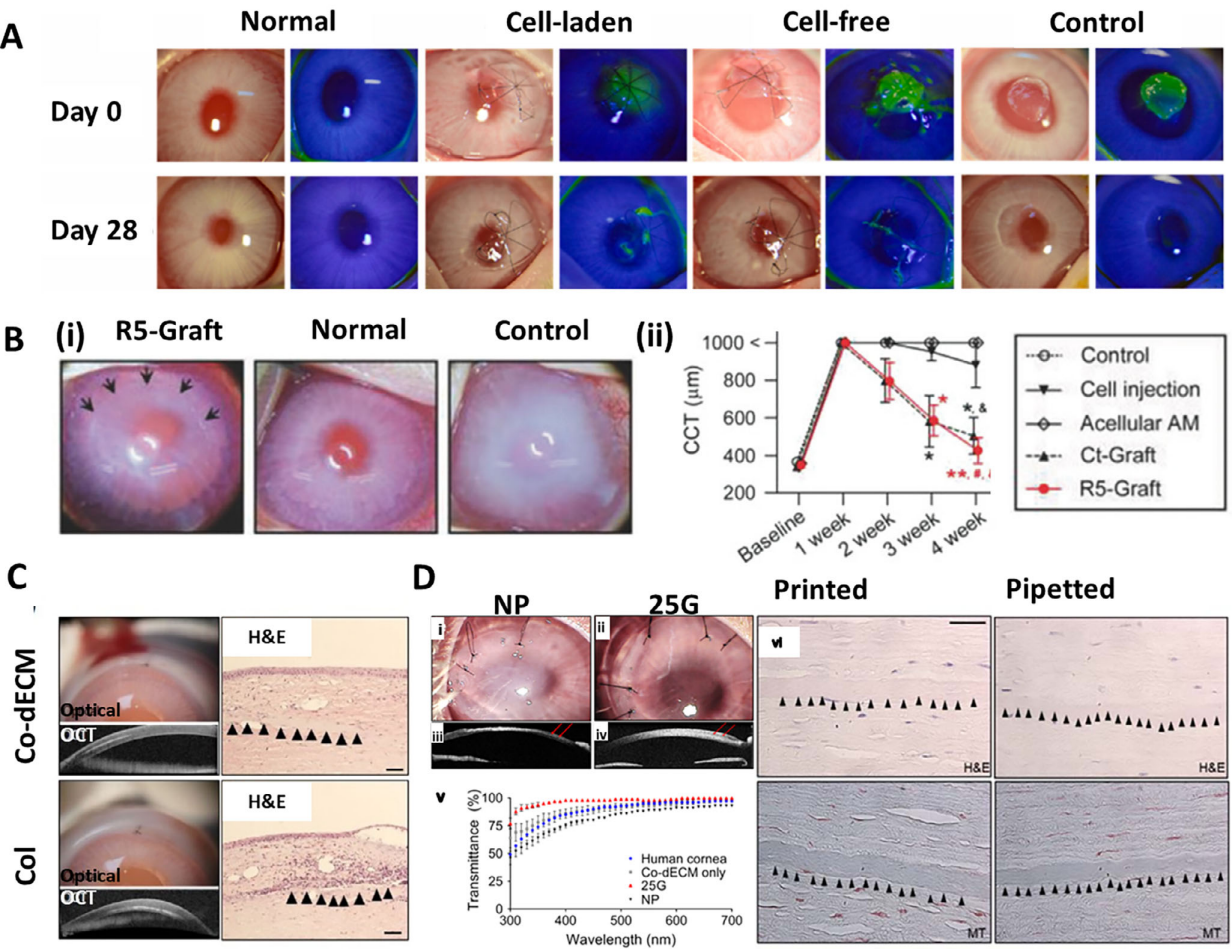
In vivo evaluation of 3D bioprinted corneal substitutes. A) Slit lamp and cobalt blue photographs of 3D bioprinted cornea, which consisted of GelMA and long‐chain PEGDA, after its transplantation into a rabbit. The green area represented a corneal epithelial defect (adapted and reproduced with permission from ref. [61] 2022, Elsevier B.V. on behalf of KeAi Communications Co. Ltd). B) i) Photographs of R5‐Graft after 4 weeks of transplantation, normal rabbit cornea, and Descemet's membrane‐stripped control cornea, which did not receive any grafts. ii) CCT graph of the control, cell injection, acellular AM, Ct‐Graft, and R5‐Graft groups (adapted and reproduced with permission from ref. [270] 2018, WILEY‐VCH Verlag GmbH & Co. KGaA, Weinheim). C) OCT images and histological analysis of the dECM and Col samples transplanted into a rabbit model (scale bar: 50 µm) (adapted and reproduced with permission from ref. [271] 2019, Sage Publications). D) i‐ii) Optical images from lamp examination after the in vivo implantation of a bioprinted cornea with collagen fibrils using EBB. NP referred to a non‐printed sample. 25G referred to needle gauge, iii‐iv) OCT images of bioprinted corneas.v) Visible light transmittance spectra of the human cornea, corneal dECM, 25G, and NP. vi) Histological images of the artificial cornea after transplantation (scale bar: 100 µm) (adapted and reproduced with permission from ref. [136] 2019, IOP Publishing Ltd).

In vivo assessment is crucial in corneal bioprinting and CTE to evaluate the functionality, integration, and long‐term stability of engineered constructs within a physiological environment.^[^ ^61^ ^]^ While in vitro models provide valuable insights into cell behavior and matrix deposition, they cannot fully replicate the complex biochemical and biomechanical cues present in a living system. Animal models, such as rabbits, are commonly used to assess bioprinted corneal grafts, allowing researchers to monitor graft transparency, immune response, neovascularization, and epithelial coverage over time.^[^ ^23^, ^61^ ^]^ Typical evaluation time points range from days to several months post‐implantation, depending on the study objectives. These assessments help determine the feasibility of bioprinted corneal tissues for clinical applications, ensuring that they can restore vision and maintain long‐term corneal function without adverse effects. For instance, He et al. bioprinted GelMA and long‐chain PEGDA using DLP to create a bilayer construct of epithelium and stroma to facilitate corneal regeneration^[^ ^61^ ^]^ (**Figure** **5**A). The bioprinted constructs exhibited excellent light transmittance and desired characteristics for cell attachment, growth, and migration. Cell‐laden hydrogel, cell‐free hydrogel, control (blank, untreated defect), and normal (healthy, unoperated eye) were evaluated in vivo utilizing a rabbit keratoplasty model. The study included 21 rabbits (2.5 kg), with surgeries performed on the right eye, while the left eye served as the normal group. Both hydrogel groups remained transparent for 28 days with no inflammation, neovascularization, or scarring, and the implants remained stable. The results suggested effective stromal regeneration, re‐epithelialization, and sealing of corneal lesions. Fluorescein staining showed progressive epithelial migration, with complete healing by Day 7 in the hydrogel groups, while the control group required 14–21 days, highlighting the hydrogel's therapeutic effect. Throughout the study, the implants remained securely in place, demonstrating successful biointegration. The study emphasized the significance of corneal constructs’ architecture and precise placement of cells for successful corneal regeneration.

In another study, Bektas et al. developed GelMA‐based construct replicating the stromal structure as well as attaining acceptable transparency, mechanical strength, and cell viability.^[^ ^104^ ^]^ The 3D construct placed in the first rabbit initially caused a fibrous reaction and later led to neovascularization. However, treatment with Maxidex and Eyelea significantly decreased vascularization and facilitated the restoration of a transparent cornea within 15 weeks. To improve the conditions for surgical applications, a GelMA hydrogel with a thinner and smaller diameter was tested on the second rabbit. The hydrogel was transplanted successfully and demonstrated minimal signs of side effects, without the need for suture fixation. Although there was initial vascularization in the cornea and a decrease in clarity, treatment with anti‐VEGF resulted in a notable enhancement in clarity. As another approach for corneal replacement, Kim et al. addressed the utilization of transplanted hCEnCs that have been genetically modified to overexpress ribonuclease 5 (R5)^[^ ^270^ ^]^ (Figure 5B). For in vivo assessment, control (no graft, rabbits without any transplantation), cell injection group (cell transfer to the desired area), acellular amniotic membrane group (transplanted amniotic membrane without cells), and Ct‐Graft (non‐transfected hCEnNCs on amniotic membrane) were studied. To create transplantable corneal endothelial grafts, hCECs suspended in a gelatin‐based bioink are extruded onto lyophilized bovine amniotic membrane. As per the study, overexpressing R5 promoted the proliferation and survival of hCEnCs in culture. Since hCEnCs typically possess a low proliferative potential in their native condition, the modified hCEnCs (R5‐hCEnCs) improved proliferation and survival rates by upregulating the R5 expression. Results revealed that, as compared to Ct‐Grafts, R5‐Grafts possessed higher cell confluency and an increased expression of the Na+‐K+ ATPase pump. Both R5‐Grafts and Ct‐Grafts exhibited enhanced corneal clarity and decreased edema following transplantation into rabbit corneas as compared to the control group, which did not receive any grafts. Increased corneal endothelial marker expression was observed with R5‐Grafts.

Toward more biomimetic approaches, researchers have used a dECM‐based bioink for corneal regeneration. The corneal dECM bioink benefits from features like native cornea regarding collagen and GAG content and transparency^[^ ^271^ ^]^ (Figure 5C). The study focused on assessing the capacity of human turbinate‐derived mesenchymal stem cells (hTMSCs) to undergo differentiation into a keratocyte lineage. Comparing dECM to natural cornea, quantitative measurement demonstrated that DNA was removed while collagen and GAG content were preserved. To assess the dECM bioink's biocompatibility, in vivo tests were carried out on mice and rabbits via corneal pocket transplantation in rabbits and subcutaneous implantation in mice. Due to thin collagen fibrils and close interfibrillar spacing, the dECM bioink demonstrated outstanding transparency. Furthermore, the expression of keratocyte‐specific markers in transplanted samples revealed that dECM stimulated tissue development in vivo. dECM indicated a notably greater region of keratocyte expression than the collagen gel. With its corneal‐specific characteristics and design flexibility provided by bioprinting, the dECM bioink obtained from decellularized cornea holds promise for corneal regeneration. In another study that utilized dECM as a bioink, Kim et al. developed a transparent artificial corneal structure for transplantation^[^ ^136^ ^]^ (Figure 5D). By exposing shear stress to the dECM bioink derived from the corneal stroma, a corneal construct with aligned collagen fibrils was created. The outcomes demonstrated that the bioprinted cornea had a high capacity for cellular alignment and tissue‐specific collagen fibril structural organization. Four weeks after implantation, bioprinted collagen fibrils imitated the natural human cornea's lattice structure. The study highlighted the value of managing collagen organization in the development of corneal tissue and the adaptability of aligned collagen fibrils. The results indicated that the collagen fibrils' arrangement caused by shear enhanced the cellular activities. In comparison to the control group, the engineered artificial cornea indicated better transparency and efficacy. **Table** **1** provides a comprehensive review of 3D bioprinted corneal substitutes, highlighting biomaterials, gel cross‐linking methods, cell types, and the significance of the listed studies.

**Table 1:** 3D Bioprinted cornea substitute.

Study	Purpose	Material	Gel Cross‐linker	Cell Type	Significance
Mahdavi et al.^[^ ^59^ ^]^	3D bioprinting of corneal stroma equivalent	GelMA	Eosin Y: Triethanolamine: 1‐vinyl‐2‐pyrrolidinone	Human Corneal Stromal Cells	Samples with 12.5% GelMA resembled native corneal like stroma with ≈82% cell viability at Day 1.
Bektas et al.^[^ ^60^ ^]^	3D bioprinting of GelMA hydrogels for cornea stroma	GelMA	Irgacure 2959 photoinitiator [2‐shydroxyl)‐4‐(2‐hydroxyethoxy)‐2‐methylpropiophenone]	Human Corneal Keratocytes	Cell viability was 98% on Day 21. Transparency of cell ‐loaded and cell‐free gels were measured over 80%.
Song et al.^[^ ^26^ ^]^	3D bioprinting with a high‐concentration, transparent collagen I bioink	Collagen I	Riboflavin	Human Epithelial Cell Human Corneal Stromal Cells	Stromal cells had over 90% viability after 3 weeks. Optimum bioink consisted of Col I, CaCl_2_, and riboflavin. Engineered corneas reached about 96% transparency.
Gibney et al.^[^ ^175^ ^]^	High‐resolution corneal tissue printing	Recombinant human Col‐III	EDC: NHS	Acellular	Reached transparency of ≥87% and elastic modulus of 506±173 kPa.
Sorkio et al.^[^ ^165^ ^]^	Laser‐ assisted 3D bioprinting of epithelial and stromal cornea	Human Col‐I Recombinant human laminin	No further cross‐linker	Human embryonic Stem cell derived limbal epithelial stem cells (hESC‐LESC), and Human adipose tissue derived stem cells (hASCs)	Bioprinted cells developed into a stratified epithelium, exhibiting apical expression of CK3 and basal expression of progenitor markers. hASCs aligned horizontally, mimicking their arrangement in native tissue.
Isaacson et al.^[^ ^122^ ^]^	3D bioprinting of corneal stroma equivalent	Methacrylated Col I Sodium alginate	CaCl_2_	Human corneal stromal cells	Keratocyte viability was >90% at Day 1 and 83% at Day 7 following the printing. Achieved bioprinting with low viscous bioink and showed the importance of successfully remodeled matrix for clinical suitability.
Kutlehria et al.^[^ ^21^ ^]^	High‐ throughput 3D printing of cornea stroma	Sodium Alginate: Gelatin typeB: type I collagen solution‐Fibricol	CaCl_2_	Human Corneal Keratocytes	The cells demonstrated >95% viability for 2 weeks. The printed mold allowed bioprinting of 6–12 corneas as a high‐throughput fabrication.
Xu et al.^[^ ^23^ ^]^	3D bioprinted convex implants for corneal tissue regeneration	GelMA: Collagen	Irgacure 2959 photoinitiator [2‐(hydroxyl)‐4‐(2‐hydroxyethoxy)‐2‐methylpropiophenone]	Rabbit corneal epithelial cells	Extrusion temperature increase prevented the step effect, which resulted in thicker periphery and thinner center. Slope of convex shape affected cells morphology and orientation by increasing their growth rate. It increased regeneration of the collagen fibers compared to control groups.
Campos et al.^[^ ^86^ ^]^	3D printing of cornea stroma model	Collagen: Agarose	No further cross‐linker	Human Corneal Stromal Cells	The cells maintained their viability and keratocyte phenotype following 7 days culture.
He et al.^[^ ^61^ ^]^	3D printing of epithelium/ stroma bilayer hydrogel implant for regeneration	GelMA: PEGDA	Lithium phenyl‐2,4,6‐trimethylbenzoylphosphinate (LAP) + photoabsorber (orange food color)	L929 mouse fibroblast cells Rabbit corneal epithelial cells Rabbit adipose‐derived mesenchymal stem cells	The implant promoted corneal regeneration by enabling re‐epithelialization and stromal regeneration. Copolymerization eliminated the brittleness of GelMA and enhanced the gel's toughness.
Mörö et al.^[^ ^114^ ^]^	Bioink for 3D printing cornea stromal model	Hyaluronic acid	Hydrazone	hASC hASC‐derived corneal stromal keratocytes Human pluripotent stem cell derived neurons	Bioink demonstrated appropriate shear thinning property, shape fidelity, printability, and regeneration property with cells.
Kim^[^ ^200^ ^]^	Evaluating the *ex vivo* function of a 3D bioprinted corneal endothelium overexpressing ribonuclease 5	Gelatin Lyophilized bovine AM	Gel‐linker (Gel4Cell®, Bioink Solutions, Inc., Daegu, South Korea)	CEnC	It maintained >90% cell viability. R5 overexpression enhanced cell proliferation by >25% and reduced apoptosis by >30%, leading to improved endothelial barrier function. The engineered endothelium demonstrated >80% transparency.
Zhang et al.^[^ ^25^ ^]^	Developing cornea using a dECM‐GelMA bioink and DLP bioprinting	Corneal dECM GelMA	Irgacure 2959 used in bioink without cells; LAP used in cell‐loaded bioink)	Human corneal fibroblasts	The corneal scaffold with >90% optical transmittance. In vitro, the bioink maintained >95% cell viability and promoted the expression of key corneal proteins. In vivo, the scaffold supported epithelial regeneration, maintained matrix alignment, and restored corneal clarity by >80%.
Wang et al.^[^ ^215^ ^]^	Engineering corneal scaffolds that mimic native cornea structure for addressing donor shortages in corneal blindness treatment.	GelMA: Hyaluronic acid	Igracure 2959 photoinitiator [2‐(hydroxyl)‐4‐(2‐hydroxyethoxy)‐2‐methylpropiophenone]	Corneal stromal cells (rabbit‐derived)	Demonstrated that GelMA‐HA scaffolds could be bioprinted and modified to enhance the behavior of corneal stromal cells. After 60 days of remodeling, decellularized scaffolds exhibited improved optical properties and guided ECM organization, essential for corneal function.
Lemonche et al.^[^ ^105^ ^]^	3D bioprinting for laser‐assisted intrastromal keratoplasty	Nanocellulose Alginate Collagen	CaCl_2_	Adipose‐derived MSCs Bone marrow‐derived MSCs Corneal stroma‐derived MSCs	The study showed the first‐time corneal construct utilizing three MSC types. Following 14 days of implantation, cells maintained their viability.

### 3.6 Challenges in 3D Bioprinting of the Cornea

3D Bioprinting for corneal tissue faces several challenges due to the specialized structure and function of the cornea. The challenges can be categorized in terms of bioprinting corneal cells, bioink limitations, achieving complex corneal architecture and specific corneal characteristics, and post‐bioprinting challenges.

#### 3.6.1 Challenges in 3D bioprinting of Corneal Cells

hCECs are vital in creating the outermost layer of the cornea, acting as a crucial defense against external elements and preserving the cornea's clarity. The superficial hCECs, with their flat and polygonal shape, are tightly bound by desmosomes and tight junctions, which establish a permeability barrier that blocks foreign substances and tears from entering the spaces between cells.^[^ ^34^ ^]^ In bioprinting, it is essential to precisely incorporate these cells into a bioprinted construct to maintain their barrier function and achieve seamless integration with surrounding tissues. However, obtaining these cells, usually derived from limbal stem cells or corneal tissue explants,^[^ ^34^ ^]^ and preserving their stemness while preventing dedifferentiation during culture and bioprinting, presents significant challenges.^[^ ^34^ ^]^

Further, it is essential to accurately position the keratocytes within a bioprinted construct to replicate the corneal architecture and function. Challenges in this process include maintaining keratocytes in a quiescent state during bioprinting, as activation or differentiation can lead to abnormal ECM production or disorganized fiber arrangement.^[^ ^272^ ^]^ Additionally, ensuring that these cells effectively produce ECM post‐bioprinting requires meticulous control of the bioprinting environment and culture conditions to support their phenotypic stability and functional performance.

Furthermore, CEnCs inclusion in 3D bioprinted corneal constructs is essential to replicate the cornea's natural functions accurately. However, several challenges complicate this process: obtaining and culturing CEnCs is difficult due to their limited availability and specialized needs; their low proliferative capacity makes it hard to achieve the necessary cell density; and precise placement is crucial to ensure proper function.^[^ ^273^ ^]^ Maintaining the correct cellular arrangement and tight junction formation is crucial to ensure that cells perform their intended role in preventing corneal edema. The “pump” function of CEnCs involves active transport mechanisms, such as the Na+/K+ ATPase pump, which helps regulate corneal hydration by removing excess sodium ions.^[^ ^34^ ^]^ This function is sensitive to the cell's physiological state and environment. Changes in the bioprinting process or post‐bioprinting conditions can affect cells’ ability to perform this function, potentially leading to fluid imbalance and loss of corneal transparency. Maintaining specialized functions of CEnCs, including their barrier and pump activities, requires careful control of the bioprinting and post‐bioprinting environments, adding significant complexity to the bioprinting process.

#### 3.6.2 Challenges Associated with Bioinks

One of the key challenges in 3D bioprinting of an artificial corneal tissue is the development of suitable bioinks. Many commonly used hydrogels fall short as ideal corneal bioinks because they fail to balance the multiple critical properties required for functional grafts. These bioinks must provide a supportive microenvironment that mimics the native corneal ECM to sustain cell differentiation, maintenance, and long‐term viability. Without an appropriate biochemical and mechanical environment, differentiated corneal cells may fail to function properly. At the same time, the bioink must possess suitable rheological properties for EBB or alternative techniques. Optical transparency and mechanical strength are often prioritized to fulfill the fundamental requirements for the cornea. Moreover, permeability is crucial for nutrient and oxygen diffusion, yet many hydrogels hinder adequate transport. Similarly, degradation rates must align with tissue remodeling, but some materials degrade too quickly or persist too long, disrupting integration. Swelling behavior also plays a significant role; excessive swelling can alter corneal thickness and impair vision, while insufficient hydration may compromise cell viability. A more holistic approach is needed, considering the complex interplay between these factors.

**Figure 6:**
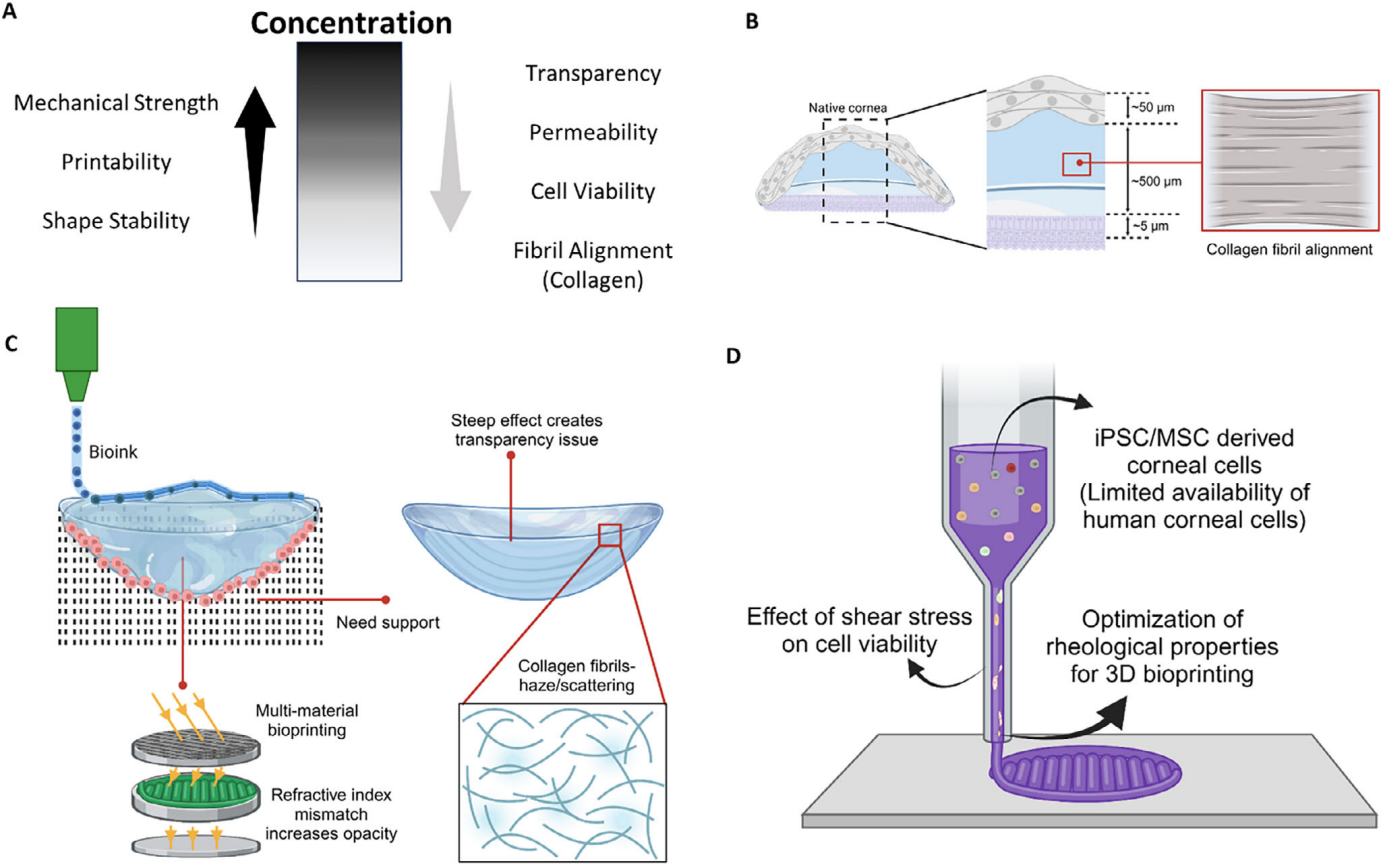
Schematic representation of key challenges in corneal tissue bioprinting. A) Effects of concentration on different physical and biological properties of corneal grafts. B) Structural complexities of the cornea, including three‐layered architecture and fibrillar alignment. C) Multimaterial bioprinting to mimic three layers of the cornea. Steeping effects from bioprinting and the refractive index mismatch due to multiple materials can lead to poor transparency. D) Challenges associated with bioprinting corneal tissue include sourcing corneal cells, shear effects on embedded cells, and rheological optimization of bioinks for seamless bioprinting. *Created with BioRender.com*.

Biomaterials used must closely mimic the corneal ECM. Hydrogels, such as GelMA and collagen, are commonly used,^[^ ^60^, ^274^ ^]^ but no single material can perfectly replicate the properties of the native cornea. Corneal transparency is a defining feature of the tissue, and bioinks used in corneal tissue engineering must maintain or improve upon this property. As seen in **Table** **2**, collagen shows a significant loss of transparency at concentrations greater than 40 mg mL^−1^ due to the increased fibrillar density. This is a critical issue for applications where the cornea's optical properties must be maintained. Also, gelatin, particularly at lower concentrations, tends to offer transparency, though its transparency decreases with higher concentrations (**Figure** **6**A). This can be a problem when gelatin is used at high concentrations for structural stability, as it may compromise the visual properties of engineered corneal tissues. Interestingly, dECM shows better transparency compared to collagen in similar conditions.^[^ ^271^ ^]^ This is likely due to the closer mimicry of the native ECM's structure, allowing for better light transmission. However, dECM bioinks can still face challenges regarding consistency and reproducibility in terms of transparency, as the ECM's composition can vary based on tissue source and processing methods.

**Table 2:** Biomaterials and their key characteristics for corneal tissue engineering.

Cornea Property	Biomaterials								
	Collagen	dECM	Alginate	Gelatin	Collagen + Alginate	GelMA + Methylcellulose	GelMA + Agarose	Corneal dECM + GelMA	GelMA +PEGDA
Transparency	Lower transparency at concentration >40 mgmL^−1^ due to high fibrillar density Thicker gels (>2 mm) resulted in less transparency^[^ ^375^ ^]^	Higher transparency than collagen in the same conditions (dECM: ≈75%; collagen: ≈50–80% in visible spectrum)^[^ ^271^ ^]^	≈88.13% transparency^[^ ^376^, ^377^ ^]^	Transparency decreases with concentration (≈ 87.53, 83.43, 76.72, and 66.35% in visible spectrum at 7, 10, 15, and 30% GelMA, respectively)^[^ ^378^ ^]^	High optical clarity (↑ vs. pure collagen)^[^ ^21^ ^]^	≈78% at 700 nm (comparable to native ≈80%)^[^ ^379^ ^]^	Matches native corneal transparency^[^ ^380^ ^]^	High transmittance (tunable; exceeds collagen)^[^ ^381^ ^]^	Increased PEGDA concentration, decreased transparency as 90.7%, 86.3%, 82.5% light transmittance at 600 nm^[^ ^61^ ^]^
Alignment	Highly aligned for <10 mgmL^−1[^ ^382^, ^383^, ^384^ ^]^	–	–	–	–	–	–	Preserves ECM alignment (in vivo)^[^ ^381^ ^]^	Printing orthogonal alignment was achieved^[^ ^61^ ^]^
Strength	Increases with concentration^[^ ^385^ ^]^	Lower than collagen (elastic modulus of the 0.3% collagen: ≈1.4 kPa; dECM: 0.6 – 1.5 kPa, depending on tissue type and decellularization technique)^[^ ^386^ ^]^	Depends on alginate concentration, ion concentration, and ion type^[^ ^387^, ^388^, ^389^ ^]^	Increases with concentration and cross‐linking degree^[^ ^390^, ^391^ ^]^	≈20 kPa^[^ ^21^ ^]^	‐ (UV‐cross‐linked GelMA network)^[^ ^379^ ^]^	G′ > G″ (viscoelastic solid behavior)^[^ ^380^ ^]^	Tunable stiffness (via GelMA cross‐link density)^[^ ^381^ ^]^	compressive modulus 100.7 kPa^[^ ^61^ ^]^
Cell Viability	Decreases with concentration due to a decrease in porosity^[^ ^392^ ^]^ >85% viability up to 40 mgmL^−1[^ ^393^ ^]^	High^[^ ^394^ ^]^	Insufficient biological functionality due to its bio‐inert property^[^ ^235^ ^]^	Decreases with concentration^[^ ^395^ ^]^	92% (day 1), 83% (day 7)^[^ ^21^ ^]^	High (promotes keratocyte adhesion and proliferation)^[^ ^379^ ^]^	High (enhanced proliferation; keratocyte phenotype maintained)^[^ ^380^ ^]^	High (maintains fibroblast viability and ECM markers)^[^ ^381^ ^]^	reach 90% over 5 days^[^ ^61^ ^]^
Printability	High printability at concentration >10 mgmL^−1[^ ^396^, ^397^ ^]^ Support bath is needed for lower concentrations^[^ ^398^ ^]^	Poor printability due to poor mechanical properties and low viscosity^[^ ^399^ ^]^ Support bath is needed for lower concentrations^[^ ^400^ ^]^	Cross‐linking mechanism is not suitable for direct extrusion printing <6 wt.%^[^ ^401^ ^]^	High printability at concentration > 6% w/v^[^ ^402^, ^403^ ^]^	Good extrusion (FRESH support)^[^ ^21^ ^]^	Printed at room temperature (Methylcellulose provides viscosity)^[^ ^379^ ^]^	‐ Extrusion printing (pneumatic)^[^ ^380^ ^]^	DLP 3D printing (photocurable GelMA)^[^ ^381^ ^]^	hydrogels containing photoabsorber exhibited a clear border and a smooth surface^[^ ^61^ ^]^

#### 3.6.3 Challenges to Replicate Corneal Architecture

Replicating the intricate structure of the cornea by achieving a precise spatial arrangement of the corneal layers and mimicking the native organization of collagen fibrils in the stroma (crucial for transparency and mechanical strength) remains difficult. The complexity of mimicking the cornea's natural curvature and thickness is further challenging, which consists of multiple layers of varying thicknesses, remains a significant challenge in bioprinting. The epithelial, stromal, and endothelial layers differ in thickness, ≈50 µm for the epithelium, 500 µm for the stroma, and 5–10 µm for the endothelium, which necessitates the use of different bioprinting techniques tailored to each layer (Figure 6B). EBB has a resolution limitation for corneal application with minimum feature sizes typically ranging from 200 to 1000 µm, making it difficult to fabricate the thin endothelial and epithelial layers, as their fine structural details fall below the resolution threshold of EBB.^[^ ^275^ ^]^ As an alternative approach, DBB, which offers sub‐200 µm resolution and precise control over cell density, may be a more suitable approach for fabricating these thinner layers.^[^ ^276^ ^]^ Additionally, in EBB the stair/step effect can often be observed as uneven or stepped surfaces on curved constructs, resulting from the discrete layer‐by‐layer deposition process. Strategies developed to eliminate the stair‐step effect in EBB could also contribute to achieving a smoother, more continuous dome‐shaped geometry. For instance, a study addressed the stair‐step artifact by tailoring a temperature‐sensitive GelMA‐collagen bioink so that, when bioprinted within an optimal thermal range, it remained fluid long enough to self‐level and fuse successive layers.^[^ ^23^ ^]^ The optimal thermal conditions for printing were achieved by maintaining the nozzle temperature between 27.5 and 30 °C, while keeping the build platform temperature at ≈20 °C. Holding the bioink at this temperature delayed its sol‐gel transition by several seconds after extrusion, allowing surface tension and gravity to smooth out each filament before rapid gelation locks in the continuous dome profile. This in situ approach removed visible ridges without requiring post‐processing or sacrificial support, preserving surface smoothness and optical clarity. While effective, producing domes that closely mimic native curvature and maintain high transmittance, the method depended on a tight temperature window and precise thermal control, which may limit its robustness and scalability across different bioprinting platforms. The researchers eliminated the stair‐step artifact by harnessing the thin liquid film that naturally adheres to each newly cured layer in their continuous DLP printing process.^[^ ^277^ ^]^ By finely tuning silicone‐hydrogel bioink viscosity and droplet diameter, they confined a uniform film between adjacent layers. Critically, this one‐step strategy suppressed both visible ridges and heat accumulation, enabling >96% optical transmittance in centimeter‐scale lenses, yet it demands a narrow window of viscosities (≈1,591–2,600 mPa·s) and droplet sizes (≈9.5–10.4 mm) to maintain that delicate balance; outside this range, either incomplete filling or excessive covering reintroduces surface irregularities. Thus, while highly effective, its reliance on precise fluidic control may limit adaptability across high‐throughput platforms.

Bioprinting the dome‐shaped cornea requires precise control to maintain its curvature and structural integrity, but soft bioinks often deform. The deformations could be primarily due to their insufficient mechanical support and low yield stress, which causes structural collapse under gravity or bioprinting‐induced forces. Additionally, without adequate cross‐linking or reinforcement, these soft materials may spread uncontrollably, leading to loss of the intended shape and poor layer fidelity. A critical limitation of conventional bioprinting on flat surfaces is that it does not inherently support the fabrication of curved geometries like the cornea, further exacerbating shape distortion. Potential solutions to mitigate these issues include embedded bioprinting, where bioinks are printed within a supporting bath of viscoelastic material, providing temporary mechanical stabilization until cross‐linking occurs (Figure 6C). This method can prevent deformation, maintain layer resolution, and allow for the controlled deposition of soft hydrogels. Another approach is bioprinting directly onto a pre‐designed curved substrate, which can guide the bioink into the correct shape and better replicate the natural curvature of the cornea. However, both techniques have trade‐offs: embedded bioprinting may introduce challenges in extracting the printed structure without damage, while curved substrates require precise alignment and compatibility with the bioink's mechanical properties to ensure proper adhesion and shape retention. Deformation of soft bioinks or gels risks irregularities that affect optical clarity.

Moreover, the orientation and organization of collagen fibrils play a key role in shaping the overall architecture of the cornea. Achieving proper fibril alignment while attempting to replicate the cornea's dome‐shaped architecture remains a significant challenge. Hybrid fabrication approaches are gaining traction for corneal tissue engineering, with recent studies combining electrospun collagen microfibers and DLP‐based photopolymerized hydrogels.^[^ ^278^ ^]^ In such a study, collagen fibers were first electrospun and chemically modified with N‐hydroxysuccinimide acrylate to introduce photosensitive double bonds. These modified fibers were then blended into a GelMA bioink, making the material compatible with DLP printing. Using this approach, the researchers successfully fabricated a dome‐shaped construct that closely mimics the natural curvature of the cornea. The reported approximately ninefold increase in tensile modulus compared to fiber‐free GelMA is striking. It addressed one of the core challenges in corneal tissue engineering: achieving sufficient mechanical strength while maintaining dome shape, optical clarity, and function. While collagen fibers likely act as passive reinforcements, the absence of controlled fiber alignment may cause inefficient and non‐uniform load transfer, which in turn affects the long‐term mechanical stability. If fibers are randomly distributed, stress concentration zones could emerge, potentially leading to anisotropic or unpredictable deformation under physiological strain. Therefore, future efforts could include the crucial step of collagen fiber alignment to address the issues raised by its absence of. Moreover, the success of this system in a 4‐week rabbit model, although promising to study short‐term integration and transparency, remains insufficient for addressing long‐term stromal remodeling. Corneal in vivo studies typically range from 4 to 12 weeks, with early assessments focusing on biocompatibility and epithelialization, mid‐term periods evaluating ECM remodeling and cell behavior, and long‐term durations are crucial for determining functional integration, mechanical stability, and sustained transparency.^[^ ^253^, ^279^ ^]^

Further, corneal stromal keratocytes are highly sensitive to microenvironmental cues, such as fiber orientation. Disorganized fibers lack the topographical guidance necessary for keratocyte alignment, actin organization, and ECM deposition fidelity.^[^ ^280^ ^]^ Without this guidance, there is a risk of aberrant ECM secretion and stromal haze formation over time, a phenomenon not captured in a 3‐day in vitro or 4‐week in vivo experiment. Nevertheless, this study represents a promising step toward next‐generation corneal implants by combining structural reinforcement with curvature fidelity to better replicate the native cornea function and support stromal cell integration. To address alignment challenges in hydrogels, fibers can be strategically positioned within hydrogels using advanced 3D printing techniques that enable in situ fiber alignment with precise spatial control. For embedded fibers in hydrogels in an aligned fashion, researchers have developed co‐extrusion systems that allow simultaneous deposition of hydrogel and fiber segments. For example, Sun et al. introduced a modified FRESH 3D bioprinting strategy in which electrochemically aligned collagen fibers were co‐printed into alginate.^[^ ^281^ ^]^ This technique enabled the creation of fiber‐reinforced constructs with an approximately six–sevenfold improvement in tensile modulus (from ≈57  to ≈383 kPa). The embedded fibers served as continuous reinforcing elements and enabled the formation of planar or helical patterns, offering design flexibility.

Another strategy is bio‐orthogonal cross‐linking that uses highly selective click reactions that form covalent bonds without toxic catalysts, creating hydrogels that gel quickly, remain transparent, and keep encapsulated cells viable. Studies utilized this methodology for more rapid and optically favorable constructs.^[^ ^130^ ^]^ In UNIversal Orthogonal Network (UNION) collagen, this chemistry produced a non‐contractile, in situ forming carrier for corneal stromal stem cells, eliminating suture‐induced trauma.^[^ ^282^ ^]^ Although the cross‐linked network provides impressive mechanical strength, its isotropic nature lacks the lamellar alignment crucial for scar‐free stromal remodeling. One example of a promising bio‐orthogonal approach is the use of a dual‐cross‐linked gelatin/alginate‐carbohydrazide ink, which combines hydrogen bonding with Ca^2^⁺/EDC curing to create extrudable hydrogels.^[^ ^283^ ^]^ These hydrogels exhibit a distinctive J‐shaped stress‐strain profile (≈1 MPa strength, >400% elongation) and high optical transparency. Using a multi‐nozzle setup, the authors fabricated a drug‐loaded bilayer construct that significantly enhanced epithelial and stromal healing in a rabbit keratoplasty model. While this work successfully overcame the inherent brittleness of natural hydrogels, it emphasized bulk strength over structural fidelity. The bioprinted filaments were stacked in parallel without incorporating 90° rotations or geodesic patterns necessary to replicate the cornea's pseudo‐orthogonal lamellae. Studies utilized the approach of orthogonal design. For instance, by 3D printing HA‐modified GelMA in an orthogonal filament grid, the authors guided stromal‐cell alignment and ECM remodeling into a transparent construct after 60 days, yet this orthogonal architecture was only validated in vitro, leaving its in vivo optical and mechanical durability untested.^[^ ^215^ ^]^ Hence, until EBB adopts this path‐aware, curvature‐aware, and feedback‐validated workflow, bioprinted corneal constructs will remain crude laminates. As another approach, a study achieved corneal anisotropy by bioprinting a single construct using two region‐specific bioinks with a soft, transparent dECM for the central zone and a mechanically reinforced dECM/sonicated silk fibroin hybrid for the peripheral region. The hybrid bioink, rich in β‐sheet structures, provides a sixfold higher stiffness than dECM alone, closely mimicking the native mechanical gradient of the cornea. By spatially patterning these materials with matched viscosities and distinct mechanical properties, the printed construct replicates the native cornea's anisotropic architecture.^[^ ^284^ ^]^

Beyond embedded fiber and cross‐linking strategies, hybrid systems that incorporate the flow of collagen through 3D printed microfluidic constructs offer a promising route to guide collagen alignment without relying on external fields. In one such study, 3D‐printed microchannel networks were designed to control the direction of collagen flow during gelation.^[^ ^285^ ^]^ By simply injecting cell‐laden type I collagen through channels oriented in different directions, it was possible to achieve layered fiber alignment, horizontal in one layer, vertical in the next, which offers potential for mimicking the native tissue organization. This simple, elegant method avoids magnetic or electrospinning setups while offering spatial control at the microscale. Although this study was not specifically designed for corneal applications, its ability to achieve multi‐directional collagen alignment using only controlled fluid flow highlights its strong potential in corneal tissue engineering. Despite its promising fiber alignment strategy, the study lacks mechanical and optical characterization, which are essential for validating the construct's physical characteristics. Hence, this approach, if integrated with detailed characterization of mechanical and transparency properties, could become highly valuable for corneal applications. However, the low collagen concentration used in this study (≈2.4 mg mL^−1^) could limit its mechanical integrity and long‐term structural stability, which is needed for a corneal graft. While this low viscosity enables smooth flow through microchannels, it also limits the potential to fabricate complex, 3D corneal constructs. Increasing collagen concentration could improve mechanical performance, but doing so may introduce flow resistance and clogging during microfluidic patterning, posing a technical challenge for scalability.

3D bioprinting has a huge potential to enable the development of corneal constructs that combine patient‐specific dome geometry with controlled collagen fiber alignment and cellular guidance. A recent study showed that modulating extrusion parameters allows spatial control of fiber orientation within bioprinted constructs, effectively directing stromal cell alignment.^[^ ^100^ ^]^ It offers a valuable framework for engineering structurally and functionally relevant corneal tissue.

#### 3.6.4 Dilemma to Achieve Optimal Mechanical Strength

The cornea needs to be mechanically stable to maintain its shape and function, while still allowing for light transmission. Collagen bioinks typically show superior mechanical strength compared to other bioinks, which is why they are often preferred for applications where structural integrity is paramount. However, collagen at higher concentrations can lead to fibrillar aggregation, which might compromise the optical properties. Gelatin, on the other hand, requires denser cross‐linking to achieve similar strength to collagen, as it does not naturally exhibit the same mechanical properties at lower concentrations. While gelatin has the advantage of being biologically active, it requires careful optimization of the cross‐linking process to prevent brittleness and maintain strength while avoiding the loss of transparency. Alginate often shows lower mechanical strength compared to collagen. Cross‐linking agents, like calcium ions, can be used to improve the strength of alginate constructs, but they can also lead to decreased biological functionality by reducing the material's ability to support cellular infiltration and growth. More research is required to achieve optimal mechanical properties in corneal grafts whilst keeping other physiological and biological properties in the acceptable range. Recent advances have employed a range of strategies to enhance the mechanical performance of corneal substitutes without compromising optical or biological functionality. One approach involves increasing the content of native ECM components. For example, incorporating 1% porcine dECM densified the hydrogel network by augmenting collagen/GAG interactions and ionic cross‐links, elevating compressive strength to ≈74 kPa without compromising transparency.^[^ ^25^ ^]^ Another strategy exploits granular architecture. Supramolecular jamming of mono‐disperse polyrotaxane microgels yielded a tightly interlocked phase, where mild heating induces crystallization of α‐cyclodextrin rings along the polymer axle, doubling bulk modulus while retaining injectability and self‐healing capacity.^[^ ^89^ ^]^ Cross‐linking design further amplifies mechanical performance.^[^ ^286^ ^]^ GelMA hydrogels that combine permanent covalent bonds with physical chain entanglement achieve an eight‐fold increase in tensile strength after sequential UV and thermal curing while maintaining flexibility.^[^ ^94^ ^]^ The addition of reversible Schiff‐base linkages further enhances mechanical resilience by enabling rapid recovery after deformation.^[^ ^95^ ^]^ Finally, integrating multiple cross‐linking modalities such as riboflavin‐mediated UV cross‐linking and Schiff‐base chemistry, has been shown to significantly improve burst pressure above 400 mmHg without loss of optical clarity.^[^ ^96^ ^]^

#### 3.6.5 Challenges Associated with Fibril Alignment

In the corneal stroma, collagen fibrils are highly aligned, a feature crucial for both transparency and mechanical strength. Achieving similar alignment in engineered constructs is essential for functional restoration. As shown in Table 2, collagen provides better alignment, especially when bioprinted at concentrations lower than 10 mg mL^−1^, which prevents excessive aggregation of fibrils. This is critical in ensuring that the engineered tissue mimics the natural corneal structure. EBB offers a distinct advantage of shear stress‐induced collagen fibril alignment, which could be leveraged to mimic the native stromal organization. The shear forces generated during EBB can facilitate fibril alignment, potentially mimicking the native structure more effectively than other bioprinting techniques. Nonetheless, while this alignment effect may benefit stromal bioprinting, the overall limitations of EBB in achieving the necessary resolution for the epithelial and endothelial layers highlight the need for multi‐modal bioprinting approaches. A combination of EBB for the stroma and higher‐resolution techniques like DBB or LAB for the thinner layers may be essential to achieving a structurally and functionally biomimetic cornea. Although multiple strategies have been explored to achieve this milestone, including the integration of advanced fabrication and alignment techniques, significant progress is still needed to fully realize this goal. Integrated strategies in corneal biofabrication are increasingly focused on merging microscale fiber orientation with macroscale anatomical precision. The goal is to create constructs that not only replicate native stromal architecture but also conform to each patient's unique corneal curvature and biomechanical properties. Techniques such as EBB, co‐printing with anisotropic fillers or guided self‐assembly during deposition, developed for general construct fabrication, could show strong potential for adaptation to bioprinting platforms, enabling simultaneous control over construct geometry and fibrillar alignment.^[^ ^287^ ^]^ These approaches go beyond static designs by introducing dynamic responsiveness and hierarchical structuring into engineered tissues. Sun et al. modified FRESH successfully, incorporating electrochemically aligned collagen fibers and offering improved mechanical properties and in‐plane organization.^[^ ^281^ ^]^ Yet, the relatively thick fibers used in their setup may compromise optical clarity, raising concerns for corneal use despite strong structural performance. The study demonstrated the potential of EBB for achieving fiber alignment; however, successful clinical translation also requires accurate replication of the cornea's dome shape and its anatomically varying thickness, which are critical for functional integration. Similarly, microfluidic‐based alignment strategies, though efficient in controlling fiber orientation, lack mechanical robustness and face challenges in developing complex 3D shapes. These shortcomings could pose translational challenges, especially for achieving curvature‐specific, load‐bearing constructs needed in corneal applications. Future strategies must prioritize material selection and bioprinting parameters that preserve optical clarity while maintaining mechanical and topographical cues. The convergence of these factors will enable scalable, patient‐specific corneal constructs that support both functional integration and long‐term transparency.

#### 3.6.6 Challenges to Achieve Transparency

The primary function of the cornea is to allow light to pass through to the retina, requiring bioprinted constructs to have a high degree of transparency. However, the bioprinting process and inherent properties of most bioinks often result in micro‐scale irregularities that lead to scattering of light, thus compromising transparency. These irregularities can arise from non‐uniform depositions of bioinks, inconsistent polymerization, or structural discontinuities in bioprinted constructs. For instance, in EBB, the layer‐by‐layer deposition may create step effects, where minor height variations between bioprinted layers cause uneven refraction of light, further compromising corneal transparency (Figure 6C). Additionally, polymer networks within bioinks and encapsulated cells can introduce refractive index mismatches, exacerbating light scattering. Hydrogels often contain inhomogeneous polymer fiber arrangements or microscale porosity, further increasing opacity. While some strategies, such as post‐print cross‐linking modifications, self‐healing bioinks, or mechanical smoothing, have been explored to improve transparency, they may inadvertently impact cell viability, bioactivity, or mechanical integrity. Moreover, while collagen fibril alignment in the corneal stroma is crucial for minimizing light scattering, EBB's shear stress can aid in fibril alignment, potentially improving transparency in the stromal layer. However, ensuring that fibrils remain consistently aligned throughout the construct remains a challenge, as variations in printing speed, nozzle diameter, or cross‐linking kinetics can disrupt this organization. Ensuring the correct alignment of collagen fibers within the stroma to prevent haze or scattering is demanding (Figure 6C). Moreover, achieving and preserving transparency, especially in multi‐material bioprinting of different layers, remains a hurdle by causing light scattering (Figure 6C).

#### 3.6.7 Cell‐Related Challenges

Cell viability is essential in ensuring that bioinks can support the growth and differentiation of corneal cells. Collagen bioinks show higher cell viability at concentrations <20 mg mL^−1^, but higher concentrations (>20 mg mL^−1^) can restrict cell migration and increase stiffness, which is detrimental to cell viability. This limitation is due to the dense fibrillar networks that form at high collagen concentrations, which hinder the diffusion of nutrients and oxygen. Higher concentrations of gelatin may lead to a restrictive environment for cells due to an increase in stiffness. This can be problematic for tissue constructs that require cell infiltration and expansion, as gelatin gels become more rigid as their concentration increases. dECM, being a more natural ECM mimic, generally shows good cell viability, but its efficacy can vary based on the source and processing methods. It is vital to ensure the proper balance between cross‐linking and mechanical stability for the best cell growth.

Cell damage during bioprinting arises from multiple physical and chemical stresses, including shear, thermal, and radiative stress, which vary depending on the bioprinting modality. Nozzle‐based methods like EBB subject cells to shear stress during deposition, with damage severity influenced by nozzle diameter, pressure, bioink viscosity, and impact velocity as highlighted in Figure 6D.^[^ ^288^, ^289^, ^290^ ^]^ Shear stress can deform or rupture membranes and activate apoptotic pathways through Caspase‐3 signaling via intrinsic or extrinsic pathways.^[^ ^291^ ^]^ To enhance printability, higher hydrogel concentrations are often used to optimize the rheological properties of the bioink. However, increasing gel concentration leads to a more viscous matrix, which can impede the diffusion of essential nutrients and oxygen, ultimately compromising cell survival. EBB using high‐viscosity bioinks generates higher pressures and prolonged shear, decreasing viability, though shear‐thinning bioinks can mitigate this decreased cell viability.^[^ ^292^ ^]^ To decrease stress on cells, the nozzle and chamber temperature can be optimized. Beyond thermal stress, the duration of the bioprinting process itself also influences cell health; extended bioprinting times can lead to reduced viability following extrusion. Studies have shown that optimizing the temperature can improve cell survival rates dramatically, increasing viability from 55.5% to as high as 90%.^[^ ^293^ ^]^ However, in a study, researchers investigated the effects of cell extrusion on the fibrin surface. The study demonstrates that bioprinting in separated lines preserved higher cell viability compared to compact patches, minimizing mechanical damage and simulating physiological wound healing.^[^ ^294^ ^]^ Another approach to improve cell viability in EBB involved using pre‐formed spheroids instead of dissociated single cells suspended in viscous hydrogels. In the study, limbal epithelial stem cell spheroids were directly bioprinted onto curved collagen membranes using a custom bioprinter, which allowed gentle, low‐pressure deposition. This method not only reduced the shear stress typically encountered during conventional EBB but also preserved critical cell–cell interactions within spheroids. The bioprinted constructs showed significantly higher viability and proliferative capacity, particularly when combined with Y‐27632, a ROCK inhibitor known to enhance cell survival. This strategy demonstrates that delivering cells in spheroid form, rather than as single‐cell suspensions, can serve as an effective alternative to traditional EBB, particularly when viability is a limiting factor.^[^ ^295^ ^]^

Additionally, other bioprinting methods, such as LAB methods, which utilize UV light for photopolymerization, present further challenges for cell viability. They introduce radiative and thermal stresses, often causing DNA damage, enzyme inactivation, and oxidative stress, particularly under UV exposure or prolonged printing.^[^ ^296^, ^297^ ^]^ Photoinitiator toxicity, especially from Irgacure 2959, significantly impacts viability, while LAP offers relatively better biocompatibility.^[^ ^298^, ^299^ ^]^ These limitations necessitate careful selection of photoinitiators and post‐processing conditions to minimize adverse effects on cell maintenance. Mechanistically, corneal cells respond markedly to substrate stiffness. The corneal keratocytes are normally quiescent in a soft, native ECM. However, pathological stiffening of the stromal microenvironment, such as that observed during fibrosis, can induce keratocyte activation into fibroblastic and myofibroblastic phenotypes, characterized by increased ECM production and contractility. This transition is strongly tied to TGF‐β1‐driven keratocyte‐myofibroblast transformation.^[^ ^79^ ^]^ In vitro studies demonstrate that when corneal keratocytes are cultured on a stiff substrate (e.g., ≈10 kPa polyacrylamide gel) in the presence of TGF‐β1, they take on broad, contractile morphologies with abundant stress fibers and high α‐SMA expression (myofibroblast markers). In contrast, the same cells on cells on a softer matrix with near‐native stiffness (≈1 kPa) remain more dendritic and quiescent.^[^ ^79^ ^]^ Interestingly, the native cornea's stiffness changes in certain scenarios, and this affects biological responses as well. An example is corneal collagen cross‐linking, a clinical procedure that stiffens the cornea (by inducing additional cross‐links in stromal collagen) to halt keratoconus progression. While effective in halting disease progression, corneal collagen cross‐linking significantly increases the corneal elastic modulus, which triggers a transient wound‐healing response. The high stiffness of the cross‐linked anterior stroma causes initial keratocyte apoptosis in that region and stimulates an inflammatory cascade. Clinically, this manifests as transient stromal haze in months after corneal collagen cross‐linking. These observations highlight the dual role of cross‐linking in both therapeutic efficacy and biological response for precise modulation of biomechanical properties in CTE.^[^ ^79^ ^]^

Material selection is compounded by the difficulties in sourcing corneal cells. While human corneal cells are limited in availability, stem cells, including iPSCs and MSCs, offer potential. However, ensuring their differentiation into the correct corneal cell type and maintaining their viability and functionality post‐bioprinting requires further optimization. The limited regenerative capacity of endothelial cells further aggravates these challenges.^[^ ^300^ ^]^ iPSCs present a promising alternative for addressing the limited availability of human corneal cells, but their successful application in bioprinting depends on the development of optimized bioinks.

#### 3.6.8 Post‐Bioprinting Challenges

Post‐bioprinting maturation and integration with the host tissue are required to achieve desired physiological and biological properties, such as proper cell alignment and ECM organization during tissue maturation. Moreover, integrating the bioprinted cornea with the native tissue without triggering immune rejection or fibrosis is a concern. Another issue is ensuring that the bioprinted cornea remains avascular while receiving adequate nutrient and oxygen supply. The native cornea is avascular, and maintaining this characteristic is essential for transparency. However, the bioprinted tissue must still be nourished, which necessitates innovative strategies to enhance nutrient diffusion without inducing vascularization.

Apart from the above‐mentioned challenges in CTE, scalability and regulatory barriers present additional challenges that could be addressed through advances in automation, biomaterials, and bioprinting technologies. Ultimately, bioprinted tissues must sustain transparency, mechanical stability, and resistance to infection over time, which requires extensive long‐term in vivo studies to thoroughly assess their performance in clinical settings.

## 4 Other Emerging Strategies

In parallel to advancements in 3D bioprinting, several innovative techniques have been developed for advancing CTE, such as the utilization of induced pluripotent cells (IPSCs), gene therapy, and cornea‐on‐a‐chip technologies. Each of these methods offers unique advantages and is discussed in brief below:

### 4.1 Induced Pluripotent Stem Cells

The primary focus of genetic research on corneal diseases has been on the identification of gene mutations, with a limited understanding of the fundamental cellular mechanisms.^[^ ^301^ ^]^ Current knowledge depends on working on cell lines and animal models, which fail to accurately replicate human physiological conditions.^[^ ^302^, ^303^ ^]^ Somatic cell reprogramming overcomes these restrictions by enabling the generation of corneal cells and organoids from iPSCs that are patient‐specific. This approach provides a more accurate representation of human corneal diseases, thereby facilitating the detailed study of the underlying molecular mechanisms. In addition, it offers possibilities for drug development, customized corneal cells for transplantation, and genome editing to develop immune‐compatible cells for potential self‐transplantation.^[^ ^304^ ^]^ For instance, Hatou et al. developed a corneal endothelial cell substitute (CECSi) from iPSCs without the need for neural crest cell differentiation.^[^ ^305^ ^]^ The cells demonstrated the presence of characteristic molecular markers of corneal endothelial cells, including N‐cadherin and PITX2. In addition, CECSi cells possessed the capability to undergo cryopreservation, and thawing, as shown by the effectiveness of cell injection therapy in a monkey model of corneal edema. Other studies demonstrate that in vitro keratocyte exposure to serum induces differentiation of fibroblasts and reduces the expression of keratan sulphate proteoglycan (KSPG), which is a marker of corneal keratocytes.^[^ ^306^, ^307^ ^]^ This highlights a potential limitation in vitro models face in mimicking native keratocyte behavior under serum‐rich conditions, which may not reflect the in vivo environment. Therefore, due to the lack of an animal model for keratoconus, iPSCs are the main tools used to mimic and study keratoconus. Joseph et al. acquired iPSCs from both keratoconus patients and individuals without the condition. The study reported a reduction in mRNA expression related to cell proliferation and differentiation pathways in keratoconus iPSCs when compared to those from normal individuals.^[^ ^308^ ^]^ The differentiation procedure involves culturing human iPSCs into embryoid bodies (EBs) in keratocyte differentiation medium for the formation of corneal stromal keratocytes (CSKs) expressing keratocan. Directed differentiation commonly uses growth factors or other small molecules^[^ ^309^ ^]^ to mimic the development of the desired cell type, such as corneal epithelium, corneal keratocytes, and corneal endothelium.^[^ ^310^ ^]^

**Figure 7:**
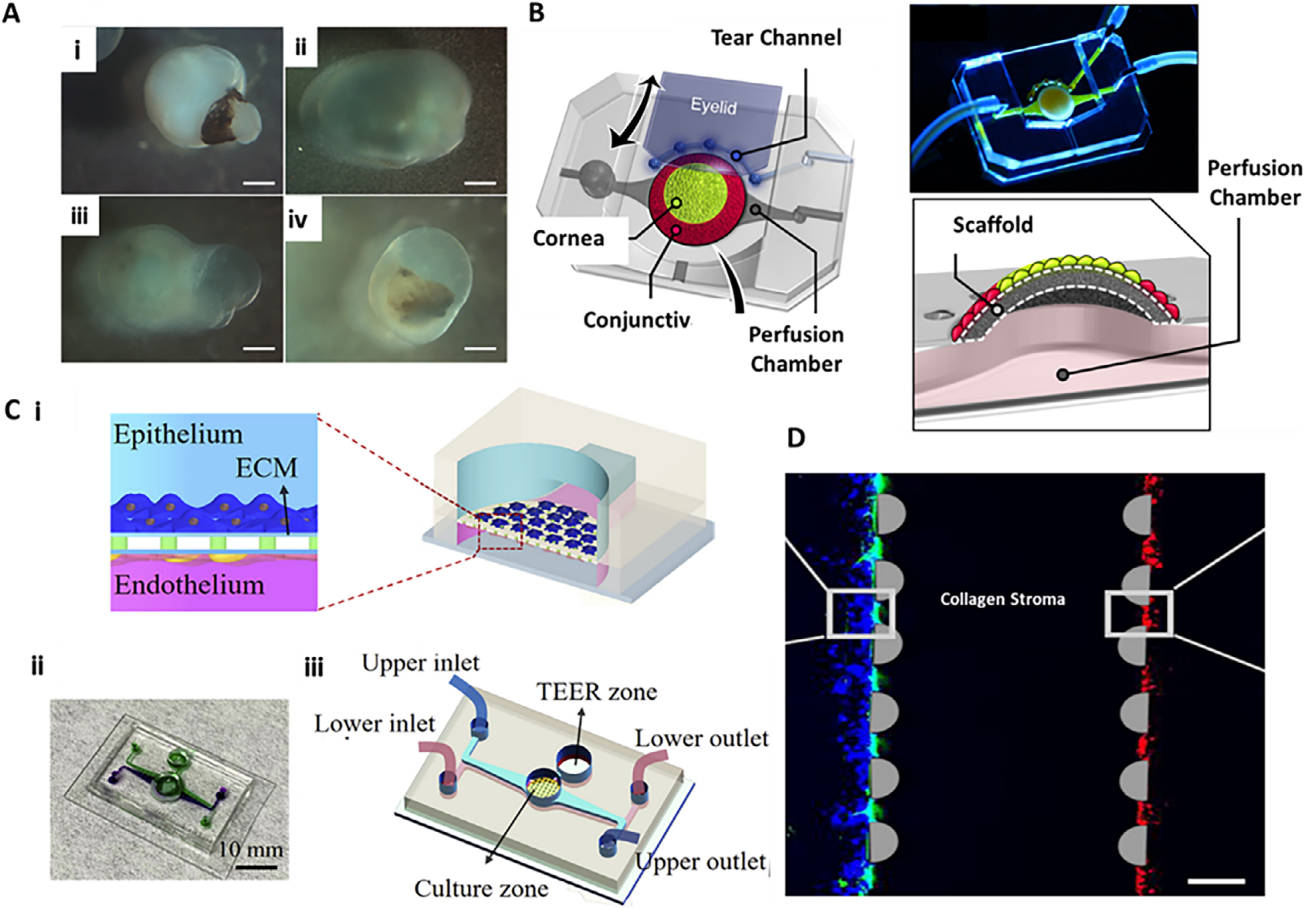
Examples of corneal constructs by emerging techniques. A) (i–iv) Bright field images of organoids. After 3 months in culture: i) translucent organoid, ii‐iv) an organoid with a clear center, iii) an organoid with a pigmented end. Scale bar: 200 µm (adapted and reproduced with permission from ref. [312] 2017, Springer Nature). B) A photograph of a multilayered elastomeric device that mimics the human ocular surface. The microfluidic model emulated the human cornea, conjunctiva, and eyelid (adapted and reproduced with permission from ref. [324] 2019, The Author(s), under exclusive licence to Springer Nature America, Inc). C) i) Schematic illustration of the corneal chip in cross‐section. On the opposing side of an ECM‐coated porous PC membrane, hCECs and hCEnCs were cultured. The membrane was encased between two PDMS layers that have microfluidic channel integration, ii) photographs of a human cornea‐on‐a‐chip; purple and green dye solutions were utilized to denote the upper and lower microfluidic structures, iii) the cornea‐on‐a‐chip, which has two distinct microfluidic channels and a hole in the center, replicates the structure of the human cornea (adapted and reproduced with permission from ref. [325] 2025 The Author(s). Published by Elsevier Inc). D) The 3D projection view of the cornea‐on‐a‐chip assay: while the epithelial cells were represented by blue, the endothelial cells were represented by red. In the middle, collagen gel mimicked the stromal structure. A dense collagen gel between the epithelial layer and stroma emulated the Bowman's membrane (adapted and reproduced with permission from ref. [326] 2020 Bai, Fu, Bazinet, Birsner, and D'Amato).

Recent advancements in 3D culture technologies have enabled the differentiation of iPSCs into organoids.^[^ ^311^ ^]^ For example, Foster et al. focused on growing retinal organoids from iPSCs obtained from human fetal fibroblasts (**Figure** **7**A). The protocol relied on promoting the development of anterior neural tissue using Matrigel, inhibiting Wnt signaling, manually dissecting developing neural vesicles, exposing the organoids to retinoic acid, and temporally limiting Notch signaling.^[^ ^312^ ^]^ Transparent organoids with corneal characteristics and important markers for endothelial, stromal, and epithelial cells were generated using this protocol. Although this method achieves layered tissue‐like organization, the complexity of the multistep protocol and dependence on animal‐derived matrices (matrigel) may limit scalability and clinical translation. In a separate work, Susaimanickam et al. created corneal organoids by inducing the differentiation of both iPSCs and ESCs into eye field primordial clusters. These clusters were then manually extracted and transferred to suspension culture to foster the growth of corneal organoids.^[^ ^313^ ^]^ This approach offers a potentially more developmentally faithful route to corneal tissue modeling. Yet, manual cluster selection introduces variability and raises concerns about reproducibility and throughput. The ability to develop corneal organoids using patient‐specific cells has the potential to be an important tool for understanding diseases of the cornea, identifying diagnostic markers, testing drugs, and developing personalized medicine. Nonetheless, future work must address challenges in functional maturation and long‐term viability if these organoids are to move beyond in vitro tools toward regenerative therapies.

### 4.2 Small Molecules and Gene Therapy

Gene therapy has recently made progress with the introduction of gene editing, which uses cutting‐edge technologies, including zinc finger nucleases (ZNFs), transcription activator‐like effector nucleases (TALENs), and CRISPR/Cas9. For instance, nucleases such as CRISPR/Cas9, provide an opportunity to edit genes, such as TCF4 and COL8A2, which are related to Fuchs' endothelial corneal dystrophy.^[^ ^121^ ^]^ Also, studies reveal that CRISPR/Cas9 shows potential in correcting genetic mutations, including CTG expansions. Challenges include the development of reliable delivery techniques to target corneal endothelial cells. It is essential to ensure safety by addressing potential off‐target effects. The above‐mentioned genetic tools, delivered using efficient carriers, enable rapid and precise manipulation of genetic materials within an organism's genome. Hence, this method shows a potential in treating congenital or inherited corneal diseases while minimizing any side effects.^[^ ^314^, ^315^, ^316^ ^]^

Corneal scarring, fibrosis, and neovascularization are the major causes of blindness worldwide, and currently lack established effective treatments. Corneal neovascularization causes blurred vision as a result of the invasion of fragile blood vessels. The potency of topical AAV serotype five delivery of the decorin gene, a strong inhibitor of TGF‐β and VEGF, offers potential in reducing corneal neovascularization and fibrosis in rats^[^ ^317^ ^]^ and rabbits.^[^ ^318^, ^319^, ^320^ ^]^ The introduction of the Smad7 gene through AAV5 also suppressed corneal fibrosis in rabbits.^[^ ^321^ ^]^ Antiangiogenic gene therapy, studied in live animal models, targets the inhibition of VEGF to block blood vessel formation.^[^ ^322^ ^]^ Gene therapy targeting VEGF could be more effective, considering the possibility of long‐term therapy, as it would eliminate the need for frequent dosing and clinic visits.^[^ ^320^ ^]^

### 4.3 Cornea‐on‐a‐Chip

Currently, in vitro culture and ex vivo studies are being primarily utilized for testing of drugs and evaluation of diseases, many of which have significant failure rates when translated to clinical settings. This is because these testing methods have limitations in replicating the complicated anatomy and physiology of the cornea. Advanced and sophisticated models are required for emulating the dynamic structure of the cornea and enhancing drug testing efficiency by reducing cost and time, and minimizing animal studies. Hence, organ‐on‐a‐chip platforms provide an outstanding testing platform for ocular drugs and provide an opportunity to comprehend the physiology of the cornea. In a study, Bennet et al. designed an integrated cornea‐on‐a‐chip, which emulates the natural characteristics of the human basement membrane, the Bowman's layer, and corneal epithelium to investigate the drug mass transport phenomenon.^[^ ^323^ ^]^ For this reason, hCECs were utilized, which were cultured on the porous polycarbonate membrane. The membrane mimicked the basement membrane and Bowman's layer to analyze permeability.

Eye blinking plays a crucial role in ocular function by providing barrier function through tear fluid distribution, which is closely associated with drug permeation.^[^ ^327^ ^]^ Studies on a rabbit model demonstrated that a high blinking rate could lead to lower interactions with ocular drugs.^[^ ^327^ ^]^ There is still room for understanding how shear stress affects the eye function during eye blinking. Abdalkader et al. reported a multi‐corneal barrier‐on‐a‐chip platform, utilizing epithelial cells to establish an epithelial barrier with bidirectional flow on the apical side and continuous flow on the proximal side.^[^ ^328^ ^]^ The study investigated the epithelial cell morphology, function, and phenotype under the flow within a microfluidic device. According to the results, significant ZO‐1 expression was achieved after seven days. Moreover, 0.6 dyn.s.cm^−2^ shear stress was unable to influence cell adhesion after 24 h. Similarly, a study proposed a miniaturized micro‐platform, which revealed a new biological perspective of blink‐induced mechanical forces^[^ ^324^ ^]^ (Figure 7B). For this model, human keratocyte‐laden constructs were fabricated and integrated with micro‐platforms. The constructs were in the center of the devices at the air–liquid interface (ALI), covered with conjunctival cells. While a perfusion chamber was utilized to provide a steady flow of medium for ALI culture, a tear channel was used to maintain humidity for the ocular surface, which was connected with a biomimetic hydrogel eyelid. Digitally controlled hydrogel eyelid movement reached the tear channel and transferred the tear through the constructs. Overall, the study provided a promising opportunity for further investigation of dry eye disease and drug screening.

In another study, Yu et al. devised a cornea‐on‐a‐chip to investigate corneal epithelial wound healing and extracellular vesicles’ effect on the healing since delayed treatment of wounds could cause blindness (Figure 7C).^[^ ^325^ ^]^ The chip consisted of a collagen‐coated membrane, human epithelial and endothelial cells for imitating stroma, the Bowman's layer, and Descemet's membrane, respectively. The study indicated that extracellular vesicles derived from BM‐MSCs (mesenchymal stem cells derived from bone marrow) enhanced cell migration and decreased inflammation and neovascularization. Similarly, Bai et al. proposed a cornea‐on‐a‐chip device, which provides cocultured primary epithelial and endothelial cells isolated from mice (Figure 7D).^[^ ^326^ ^]^ The cells were cultured on the outer channels of the chip, with collagen matrix at the center, mimicking the stromal structure of the cornea. A collagen matrix formed in the epithelial region of the device by creating a hydrogel lumen, which functioned as the Bowman's membrane. According to the results, the diffusive permeability of the model was similar with and without the presence of an endothelial cell layer. On the other hand, permeability was significantly higher when the epithelial cell clusters did not exist, which indicated that the epithelial layer had a valuable role in modulating the drug diffusion rate.

### 4.4 Artificial Intelligence and Machine Learning Applications in Corneal Bioprinting

Artificial intelligence (AI) and machine learning (ML) are increasingly being integrated into the field of biofabrication, with their role expanding over time. Real‐time monitoring of bioprinting process parameters enables the continuous collection of live data, which can be analyzed by AI models to predict bioprinting outcomes. AI‐based closed‐loop feedback systems for 3D printing can monitor 3D printing process for detecting and correcting the defects, such as warping, over/under‐extrusion, and delamination in EBB^[^ ^329^ ^]^ and droplet properties, such as droplet diameter, circularity, and coalescence in DBB.^[^ ^330^ ^]^ In the future, advanced models are expected to not only forecast printing results but also dynamically adjust printing parameters in situ, thereby enabling real‐time optimization of the fabrication process.^[^ ^331^ ^]^

In corneal tissue engineering, AI has demonstrated significant potential in both diagnostic and prognostic applications. AI models have been employed to predict the onset and progression of corneal diseases, as well as to estimate the likelihood of graft success following transplantation. For example, an ML model was developed to detect kerotoconus with an accuracy of up to 99.3%.^[^ ^332^ ^]^ In another study, an AI model was utilized to automatically identify graft dislocation following Descemet's membrane endothelial keratoplasty, achieving an accuracy of 96%.^[^ ^333^ ^]^ Additionally, AI‐driven microscopic analysis has been applied for the segmentation and evaluation of corneal endothelial layers, enabling objective assessment of corneal health.^[^ ^334^ ^]^ However, the performance and reliability of AI‐based diagnostic systems must be rigorously evaluated and validated by experienced ophthalmologists to ensure clinical accuracy and to mitigate the risk of erroneous or misleading outputs generated by the algorithm.^[^ ^335^ ^]^ Beyond diagnostics, AI holds promise in the design and fabrication of corneal constructs. By analyzing high‐resolution corneal scans, AI can generate anatomically accurate 3D models for use in bioprinting patient‐specific grafts. Furthermore, large language models can be trained to predict the likelihood of graft integration and long‐term stability post‐implantation. While the reliability of such models depends on access to large, high‐quality datasets,^[^ ^332^ ^]^ several studies have demonstrated their robustness and clinical relevance.^[^ ^333^ ^]^

AI offers a powerful framework for enabling precision medicine in the management of corneal disorders. By analyzing patient‐specific datasets, including clinical history, high‐resolution ocular imaging, and individualized physiological parameters, AI‐driven models can be used in the future to generate tailored design specifications for corneal constructs. These specifications may encompass geometric attributes such as thickness, diameter, and curvature, and in more advanced applications, extend to physical properties like stiffness, degradation kinetics, and viscoelastic behavior. Such personalized design has the potential to significantly enhance therapeutic efficacy and patient‐specific clinical outcomes.

## 5 Corneal Implants and the Clinical Perspective

The human cornea is crucial for vision, and therefore, it is necessary to replace this avascular structure in cases of congenital or acquired problems. Toward this, artificial corneas can provide significant benefits in terms of ease, safety, affordability, and rapid application for both surgeons and patients. Until now, corneal transplantation has primarily relied on human donor tissues obtained from deceased individuals. In rare cases, a healthy cornea may be used if the eye is removed for other reasons. Prior to extraction, it is preferred that the donor has not undergone any surgical procedures on the cornea or within the eye. Thus, for its transplantation, the cornea should have a standard healthy structure and not have been directly or indirectly traumatized due to intraocular surgery. Despite undergoing evaluation in eye banks, extracted corneas exhibit non‐standardized structures due to their human origin and inherent variable characteristics, such as cell count and distribution, stromal collagen structure and arrangement, corneal thickness and thickness distribution, and corneal geometric properties.^[^ ^336^, ^337^ ^]^ Additionally, standardized tests are conducted to detect diseases that can be transmitted through corneal transplantation; the presence of unknown pathogens in corneas that pass these tests may still pose risks.^[^ ^338^ ^]^ Due to the immunological nature of the transplanted corneas, the use of locally and systemically immunosuppressive agents may be necessary to prevent rejection by the recipient. The long‐term use of these drugs, both locally and systemically, can lead to unavoidable side effects. However, it is currently not necessary to consider human leukocyte antigen (HLA) compatibility between the recipient and the donor for corneal transplantation.^[^ ^339^ ^]^ Also, it is evident that an artificial cornea, which contains all the native corneal cells and is immunologically inert, would perform better regarding tissue compatibility. Although corneas that have good compatibility can survive for very long periods, it is known that donor corneas have a shorter lifespan, can deteriorate, and may require regrafting.^[^ ^340^, ^341^ ^]^ To overcome this, artificial corneas can have a more predictable, controllable, and restorative lifespan depending on their base materials and cell structure. Additionally, graft replacement could be more accessible due to the ease of obtaining artificial corneas. Despite corneal transplantation being performed in many countries worldwide, access to corneas may be limited due to country‐specific conditions, legal issues, beliefs, societal perspectives, and limitations in finding suitable donors. Therefore, introducing artificial corneas would greatly facilitate the treatment process for patients and surgeons.

Corneal surgical approaches vary based on the depth and location of the pathology. Penetrating keratoplasty (PK) is a full‐thickness transplant that replaces all corneal layers and is typically indicated for advanced keratoconus, deep corneal scarring, corneal dystrophies, and failed prior grafts. In contrast, deep anterior lamellar keratoplasty (DALK) selectively replaces the anterior stromal layers while preserving the host endothelium and Descemet's membrane, making it suitable for keratoconus without endothelial damage, stromal dystrophies, and non‐infectious anterior scarring. For diseases limited to the endothelium, such as Fuchs’ endothelial dystrophy^[^ ^342^ ^]^ or pseudophakic bullous keratopathy, endothelial keratoplasty, including Descemet's Stripping Endothelial Keratoplasty (DSEK) and more selective Descemet's Membrane Endothelial Keratoplasty (DMEK), is preferred. DLEK (Deep Lamellar Endothelial Keratoplasty) is an earlier form of endothelial keratoplasty in which the posterior layers of the cornea, specifically the endothelium and part of the posterior stroma, are removed and replaced with a donor graft containing healthy endothelium and posterior stroma. When conventional keratoplasty is not feasible due to high risk of graft failure (in severe ocular surface disease, autoimmune conditions, multiple failed grafts), keratoprosthesis (KPro) implantation is considered.^[^ ^343^ ^]^

Currently, even though the survival rates of the grafts are good, in some cases, conventional keratoplasty is not recommended due to high risks. Food and Drug Administration (FDA)‐approved corneal implants have undertaken a great mission in meeting these needs. **Table** **3** highlights the status of clinical trials for corneal implants. Boston keratoprosthesis type 1 (BKPro), created at the Massachusetts Eye and Ear Infirmary in 1992,^[^ ^249^ ^]^ includes a front plate (PMMA) with optical characteristics, a back plate, and a donor cornea button.^[^ ^344^ ^]^ In 2003 and 2007, a titanium locking ring and a newer stem were added to reduce manufacturing costs and graft damage during surgery,^[^ ^345^, ^346^ ^]^ respectively. In 2019, the new FDA‐approved device, ‘Lucia keratoprosthesis’ was released as a BKPro model.^[^ ^344^ ^]^ The BKPro I is implanted using a modified penetrating keratoplasty (PK) technique, typically for patients with multiple failed corneal grafts but an otherwise healthy ocular surface. Several studies stated that BKPro type‐I implantation reached a visual acuity more/equal to 20/200 for more than 70% of eyes during 2 years of follow‐up.^[^ ^347^, ^348^, ^349^ ^]^ BKPro Type II is also an FDA‐approved corneal implant like Type I, mostly designated for severe end‐stage ocular surface disease desiccation. The BKPro Type II involves a more invasive procedure where the prosthesis is implanted through a surgically closed eyelid, used in patients with severe ocular surface disease such as Stevens‐Johnson syndrome or ocular cicatricial pemphigoid.^[^ ^350^ ^]^ Due to the poor and temporary integration of BKPro, especially in patients who are not suitable candidates for transplantation, CorNear KPro (CorNeat Vision Ltd, Raanana, Israel) was developed. The CorNeat KPro designed for integration into the subconjunctival space and is implanted using a novel procedure that does not rely on donor tissue, making it suitable for patients with total corneal blindness and in regions with limited tissue access.^[^ ^351^ ^]^ The implant included a central optical element made by PMMA and an external skirt developed by electrospinning carbonated poly‐urethane fibers. For facilitating corneal implantation, the design consisted of a corneal groove for placing on the corneal stump of the patient, three holes for suturing, and skirts were designed to integrate with the conjunctiva, which leads to fibroblasts from Tenon's capsule to the skirts. As a short‐term outcome, the implant achieved an improvement in visual acuity.^[^ ^351^ ^]^ Auro KPro (Aurolab, India) is another keratoprosthesis and the design is like BKPro, which is indicated for opacified corneas. However, it improved the vision only in the central region.^[^ ^352^ ^]^ A different type of corneal prosthesis, known as the osteo‐odonto‐keratoprosthesis (OOKP), initially developed by Strampelli and later modified by Falcinelli (MOOKP), has been shown to yield the most favorable visual outcomes. It has been backed by extensive long‐term follow‐up data, demonstrating its effectiveness in restoring vision for patients suffering from advanced ocular surface disease.^[^ ^353^, ^354^ ^]^ The process of implantation relies on the removal of the lamina of the tooth, drilling a hole near the lamina, and fitting a cylindrical lens into this hole. The lamina is left to grow in the patient's cheeks before transplantation. After modification of the procedure, PMMA is used to anchor the lamina to the cornea.^[^ ^355^ ^]^ As an outcome worldwide, 78% of patients reached a 20/400 or better visual acuity after the follow‐up period.^[^ ^356^ ^]^ Another keratoprosthesis is synthetic and biocoated MIRO CORNEA UR (Miro Vision, South America), which is indicated for bilaterally blind eyes. The flexible haptic is treated with genetically engineered fibronectin for integration into host tissue. After implantation into four corneally blind patients (autoimmune disease), 3 of the implants were adherent and integrated into the haptic for up to 52 months without any inflammation.^[^ ^357^ ^]^

**Table 3:** Status of clinical trials for corneal implants.

Artificial Cornea	Material	Design Features	Indication/ Use	Outcome/Effectiveness	References
Boston keratoprosthesis type 1 (BKPro)	PMMA, Titanium	Front plate (PMMA), back plate, donor cornea button, titanium locking ring, and newer stem.	Severe ocular surface disease, not suitable for transplantation	Visual acuity ≥ 20/200 in >70% of eyes during 2 years follow‐up for Type I; Type II for severe end‐stage ocular surface disease desiccation	[347, 348, 349, 404]
CorNear KPro	PMMA, Carbonated polyurethane	Central optical element, external skirt (electrospun polyurethane fibers), corneal groove, three suturing holes, designed to integrate with the conjunctiva	Severe ocular surface disease, not suitable for transplantation	Improvement in visual acuity, biointegratable, does not require a donor cornea	[351]
Auro KPro	PMMA	Central optical component made of PMMA, support structure with a skirt or flange for stabilization, uses a donor corneal button, fixation through sutures	Opacified corneas	Improved vision, but only in the center region	[186, 352, 353, 354, 356, 405]
Osteo‐odonto‐keratoprosthesis (OOKP)	Tooth lamina, PMMA	Cylindrical lens fitted into a drilled hole in the tooth lamina, lamina grown in the patient's cheek before transplantation, PMMA anchors lamina to the cornea	Advanced ocular surface disease	78% of patients achieved 20/400 or better visual acuity	[353, 354, 356, 405]
MIRO® CORNEA UR	Synthetic material, biocoated with fibronectin	Flexible haptic, genetically engineered fibronectin coating for integration into host tissue	Bilaterally blind eyes (e.g., due to autoimmune disease)	3 out of 4 implants were adherent and integrated for up to 52 months without inflammation	[357]
Presbia Flexivue Microlens™	Hydroxyethyl methacrylate and methyl methacrylate	3 mm diameter lens, surgically implanted into a corneal pocket formed by a femtosecond laser, alters the corneal refractive index	Presbyopia	73.3% of patients achieved good vision after a 3‐year follow‐up	[363, 364]
Raindrop Near Vision Inlay	Permeable hydrogel	2 mm diameter, 32 µm thickness, reshapes corneal curvature, facilitates eye focus	Presbyopia	92% of patients achieved 20/40 near vision or better; however, corneal haze was a common problem	[366, 367]
Kamra inlay	Polyvinylidene fluoride and carbon (PVDF)	Ring‐shaped, aimed at clear vision for middle‐aged patients without cataract	Presbyopia	83.5% of patients achieved 20/40 or better vision at 12 months	[368]

Cornea inlays (keratophakia) are another type of prosthesis positioned in the corneal stroma to correct presbyopia, which is the gradual loss of ability to focus on nearby objects.^[^ ^358^ ^]^ Corneal inlays were introduced by Barraquer to alter corneal refraction.^[^ ^359^ ^]^ Initially, donor corneal tissues and synthetic materials such as PMMA, polypropylene, and silicon oil were used, but these materials often resulted in the extrusion of the implant and stromal necrosis.^[^ ^360^, ^361^ ^]^ To address these issues, water‐impermeable devices were developed. However, these devices prevented nutrient transportation and water flow through the cornea, which led to corneal thinning. For the necessity of water permeability, hydrogel‐based corneal inlays, such as glycerin methacrylate hydrogel, were designed.^[^ ^362^ ^]^ The Presbia Flexivue Microlens (Presbia, Irvine) is a 3 mm diameter lens that is surgically implanted into a corneal pocket formed by a femtosecond laser.^[^ ^363^ ^]^ It has obtained the Conformité Européenne (CE) mark for the European Economic Area, enabling its commercial distribution throughout Europe. However, it has not yet gained approval from the FDA and is not available for commercial use in the US. This inlay operates by altering the refractive index of the cornea, with reports indicating that 73.3% of patients (out of thirty patients) achieved good vision after a 3‐year follow‐up.^[^ ^364^ ^]^

FDA‐approved inlays in the market include the Raindrop Near Vision Inlay (formerly PresbyLens; ReVision Optics, CA) and the Kamra inlay (AcuFocus, CA). Raindrop Near Vision Inlay aimed to provide correct refraction by reshaping the curvature of the cornea and a steeper surface to facilitate eye focus. Devices are placed within a femtosecond laser‐created stromal pocket during keratophakia, a refractive procedure used to correct presbyopia rather than structural corneal disease. The inlay is made up of permeable hydrogel with a 2 mm diameter and 32 µm thickness.^[^ ^365^ ^]^ According to the results of inlay transplantation to eight patients, the corneal shape changed after one year of implantation, with corneal haze as a common problem.^[^ ^366^ ^]^ Moreover, a larger study performed with 373 patients demonstrated that 92% of patients reached 20/40 near vision or better.^[^ ^367^ ^]^ Ring‐shaped Kamra Inlay aimed to provide clear vision for patients between 45 to 60 years old who had no cataract surgery. According to results, 83.5% of 478 patients reached 20/40 or better at 12 months.^[^ ^368^ ^]^

Tissue‐engineered corneas have the potential to tackle keratoprosthesis challenges, aiming for native‐like corneas. 3D Bioprinting, due to its advantages, has been proven successful in various tissues, emerging as a viable option, and is currently under extensive investigation, as exemplified earlier. Nonetheless, there remains a significant road ahead to transition tissue‐engineered corneas from the experimental phase to clinical trials. This advancement requires progress in scaling up 3D bioprinting methodologies, including the utilization of advanced biofabrication techniques and enhanced bioinks, alongside a deeper understanding of corneal anatomy, transparency, physiology, and mechanical characteristics.

## 6 Future Directions for 3D Bioprinting of Cornea

Future research directions for 3D bioprinting of the cornea include a multidisciplinary approach aiming at optimizing various aspects of the bioprinting process. This includes enhancing the biomechanical and biofunctional properties and transparency of bioprinted corneas to ensure better integration and visual clarity post‐transplantation. Continued innovation in bioink development and consideration of regulatory standards will be crucial. The ongoing efforts involve the development of bioink formulations to optimize their biocompatibility and mechanical characteristics. Simultaneously, efforts are directed toward improving their ability to support the growth and differentiation of corneal cells. Integration of corneal endothelium in a bioprinted cornea is very challenging since not all biomaterials support the adhesion and formation of a high‐density monolayer of hCEnCs. Development of stromal bioinks that also allow hCEnC adhesion and monolayer formation is critically needed. Scientists strive to discover innovative biomaterials and enhance their composition to develop bioinks that closely emulate the inherent characteristics of the cornea, thereby fostering efficacious corneal regeneration. Moreover, the integration of bioactive agents within bioprinted corneas exhibits significant potential. Future research attempts will encompass the implementation of regulated release mechanisms for growth factors and bioactive molecules to augment the process of tissue regeneration and optimize the enduring efficacy and robustness of the transplanted corneas. Through the utilization of advanced bioprinting methodologies, researchers strive to advance the development of corneal implants that exhibit a high degree of resemblance to the anatomical characteristics and operational capabilities of the native cornea. In addition, by leveraging patient‐specific data, such as corneal topography and biometric measurements, bioprinting can produce corneal grafts that are tailored to fit the exact curvature and dimensions of the patient's eye. This personalized approach is particularly advantageous for patients with complex ocular conditions, irregular corneal anatomy, or those requiring customized grafts following previous surgeries, where standard corneal grafts may not provide optimal outcomes.

Integration of 3D bioprinting with other techniques has the potential to address several challenges. The precision of bioprinting in controlling the high‐throughput deposition of iPSCs derived embryoid bodies and organoids will allow the fabrication of complex corneal models. Bioprinting of iPSC aggregates renders it a valuable tool for advancing the applications of iPSC aggregates.^[^ ^369^, ^370^ ^]^ Bioprinting with retinal organoids offers promising potential to develop models for personalized medicine and drug testing. 3D Bioprinting also enables a dynamic and efficient platform for real‐time gene modification technologies. Bioprinted constructs possess the ability to encapsulate growth factors, stem cells, and nucleic acids, which could establish a stable environment that releases small molecules. In addition, 3D bioprinted constructs also allow for the manipulation of gene expression or improvement.

To transition from laboratory research to clinical application requires scalability, reproducibility, and regulatory approval pathways to be addressed. Additionally, advancing patient‐specific customization, conducting long‐term safety and efficacy studies, and developing cost‐effective bioprinting technologies are key priorities. To advance the utilization of bioprinted corneas in clinical settings, it is imperative to undertake comprehensive preclinical and clinical validation. Future directions are expected to include the implementation of preclinical investigations aimed at assessing the safety, effectiveness, and enduring consequences of bioprinted corneas in animal models. Conducting extensive clinical trials will be imperative to evaluate the safety and efficacy of these implants in human subjects, establish universally accepted protocols, and collect comprehensive data on their long‐term performance. The convergence of progress in bioink materials, bioprinting methodologies, incorporation of bioactive agents, and thorough examination and verification will play a significant role in the advancement of operative and biocompatible bioprinted corneas. These hold promises for enhancing visual acuity and overall well‐being among individuals requiring corneal transplantation.

### 6.1 Material Design for Clinical Translation

A persistent obstacle in corneal tissue engineering is that lab‐formulated materials rarely account for the anatomical diversity of real patients. A child's cornea, for instance, is thinner and more compliant than that of an elderly keratoconic patient, and population studies show that the average corneal thickness and stiffness vary by nationality and ethnic background, yet most hydrogel recipes still target a single, generic modulus.^[^ ^7^, ^88^, ^371^ ^]^ Closing this gap will require shifting material design from one‐size‐fits‐all chemistries toward tunable platforms whose stiffness, hydration, and optical index can be dialed in case by case. Material selection and composite fabrication strategies play a critical role in determining the performance of artificial corneas. Physical blended systems, such as collagen‐gelatin, gelatin‐alginate, dECM‐in‐hydrogel, rely on noncovalent interactions,^[^ ^372^ ^]^ which can enhance cytocompatibility and transparency by avoiding initiators/cross‐linkers. However, these systems often compromise on mechanical strength, suture handling, and retention, and shape fidelity. In contrast, chemical conjugated materials, such as GelMA, PEGDA‐GelMA, catechol‐HA, and aldehyde‐modified polysaccharides, form covalent networks that improve material toughness and dimensional stability. Nevertheless, they may introduce initiator toxicity, residual byproducts, and chromophores that impair optical transmission. These trade‐offs highlight the importance of tailoring both material composition and fabrication strategy to balance transparency, mechanical integrity, and biocompatibility in artificial corneal constructs.^[^ ^373^ ^]^ Hybrid gels and core‐shell designs, such as constructs with a bioactive core surrounded by a mechanically robust hydrogel shell, offer a promising strategy to decouple optics from mechanics.^[^ ^374^ ^]^ In such a strategy, robust alginate/PEGDA shell maintains construct shape and provides compression recovery, while the collagen/matrigel/GelMA core mimics the ECM, enabling nutrient transport, vascular‐like network formation, and sustained albumin secretion by encapsulated cells.

For cell‐first applications, cross‐linker‐free, physically blended systems are advantageous due to their lower toxicity, better phenotype, and potentially higher transparency. However, these benefits often come at the cost of reduced mechanical strength compared to chemically conjugated hydrogels. Overall, explicitly choosing and combining materials to make blends or chemical conjugates should balance optical clarity and biocompatibility against mechanical robustness and manufacturability.

To address the critical requirement for corneal graft materials that can accommodate patient‐specific variability, arising from factors such as age, ethnicity, and pre‐existing ocular conditions, it is essential to develop a material system composed of raw components derived from standardized, well‐characterized sources to ensure reproducibility. Moreover, the material should possess tunable physical properties, enabling customization through controlled variation of its composition or design parameters to match the biomechanical and structural demands of diverse patient profiles. dECM offers a native blueprint, but batch‐to‐batch variability, and its non‐human sourcing in many studies, limits its reproducibility. In advanced clinical settings, emerging bioprinting technologies may enable surgeons to fabricate and photopolymerize patient‐specific constructs intraoperatively, conforming precisely to the anatomical topography while preserving essential bioactive motifs. However, successful implementation of such procedures necessitates that clinicians receive specialized training in bioprinting techniques and develop a comprehensive understanding of the material systems involved, including their cross‐linking chemistries. To facilitate this, a detailed material reference guide is essential, providing real‐time guidance on the selection and tuning of material formulations to achieve desired in situ properties such as optical transparency, mechanical stiffness, and 3D architectural fidelity tailored to individual patient needs. Future work must therefore pair patient‐derived biomechanical data (age, pathology, and curvature maps) with modular chemistries and real‐time cross‐link‐monitoring to ensure that elasticity, strength, and clarity converge in a single construct. The integration of ophthalmologists’ diagnostic metrics with materials‐by‐design holds the potential to develop bespoke corneal grafts, facilitating the transition from promising prototypes to clinically viable and regulatory‐approved therapies.

These needs highlight the broader direction needed to bridge the translational gap in corneal biomaterials. An iterative, clinician‐informed design that delivers tunable formulations (viscosity, cross‐linking density, curing speed, and degradation kinetics) adaptable to patient‐specific anatomy, and even ethnic or age‐related differences in corneal biomechanics. Only by shifting from generic “off‐the‐shelf” polymers to modular platforms can we enable the seamless transition from bench to bedside and truly meet individualized clinical needs.

## 7 Conclusion

3D Bioprinting has significantly advanced the field of CTE, providing potential solutions for the global shortage of donor corneas and enhancing the success rates of corneal transplantation. It enables the fabrication of corneal constructs that resemble the complex arrangement and composition of the native cornea. Various bioinks, such as collagen‐ and gelatin‐based bioinks have been employed in the bioprinting of corneal constructs. These biomaterials provide physical support, promote cell attachment, and enable the incorporation of bioactive signals that are necessary for the proliferation of cells and the regeneration of tissues. Various bioprinting techniques have been used to develop corneal constructs with precise spatial control and high resolution. Each technique has unique advantages, including the high‐throughput capability and the ability to preserve cell viability and bioprint multiple bioinks simultaneously, which contribute to the versatility and effectiveness of 3D bioprinting in CTE. Bioprinted corneal constructs offer noticeable benefits as compared to conventionally engineered corneas. The precise control over microarchitecture and composition enables the accurate replication of the complex organization of the corneal stroma, which leads to enhanced tissue function and improved biomechanical properties. Furthermore, the patient‐specific customization of bioprinted corneal constructs enhances the integration process after transplantation. Overall, the precision and reproducibility of 3D bioprinting technologies enable the on‐demand development of corneal constructs, meeting the world's expanding need for corneal transplantation.

## Conflict of Interest

I.T.O. has an equity stake in Biolife4D and is a member of the scientific advisory board for Biolife 4D and Healshape. The remaining authors declare no competing interests.
